# Pancancer analysis of DNA methylation-driven genes using MethylMix

**DOI:** 10.1186/s13059-014-0579-8

**Published:** 2015-01-29

**Authors:** Olivier Gevaert, Robert Tibshirani, Sylvia K Plevritis

**Affiliations:** Biomedical Informatics Research, Department of Medicine, Stanford University, 1265 Welch Road, Stanford, CA 94305 USA; Departments of Health Research & Policy, and Statistics, Stanford University, Stanford, CA 94305 USA; Department of Radiology, Stanford University, Stanford, CA 94305 USA

## Abstract

Aberrant DNA methylation is an important mechanism that contributes to oncogenesis. Yet, few algorithms exist that exploit this vast dataset to identify hypo- and hypermethylated genes in cancer. We developed a novel computational algorithm called MethylMix to identify differentially methylated genes that are also predictive of transcription. We apply MethylMix to 12 individual cancer sites, and additionally combine all cancer sites in a pancancer analysis. We discover pancancer hypo- and hypermethylated genes and identify novel methylation-driven subgroups with clinical implications. MethylMix analysis on combined cancer sites reveals 10 pancancer clusters reflecting new similarities across malignantly transformed tissues.

**Electronic supplementary material:**

The online version of this article (doi:10.1186/s13059-014-0579-8) contains supplementary material, which is available to authorized users.

## Background

DNA methylation is being increasingly recognized as an important process underlying oncogenesis [1]. Besides genetic mutations and copy number alterations, differential methylation is an alternative mechanism that is capable of altering the normal state and driving a wide range of cancers [1-3]. Recent studies have identified DNA methylation, including genome-wide DNA methylation, in normal tissues and cancer [1,4-6]. Irizarry *et al.* [5] concluded that DNA methylation is mostly located in CpG shores and conserved between human and mouse. Ruike *et al.* [4] analyzed DNA methylation in breast cancer cell lines and showed that methylation is altered during the epithelial to mesenchymal transition. Hon *et al.* [7] showed that extensive hypomethylation is present in intergenic regions in breast cancer and is mutually exclusive with repressive histone methylation (that is, H27K3me3 and H3K9me3). Berman *et al.* [6] identified focal regions of hypermethylation within long-range regions of hypomethylation using sequencing in colorectal cancer. Collectively, these studies are beginning to reveal a methylation map that is critical to understand epigenetic drivers of cancer.

Many prior studies have identified hypo- or hypermethylation of cancer based on heuristic measures (reviewed in [8]). However, few studies formalize the identification of DNA methylation-driven genes using a model-based approach. We propose a method called MethylMix that aims to derive key methylation-driven genes in cancer based on three key criteria. First, the determination of the degree of methylation should not rely on arbitrary thresholds. Second, the identification of a cancer gene as hypo- or hyper-methylated should be made by comparing its differential methylation state in cancer versus normal tissue. Finally, the identification of genes that are hypo- and hypermethylated in cancer and likely drivers should be selected as having a significant predictive effect on gene expression, thereby implying that their methylation is predictive of transcription and thus functionally relevant.

Here we present and apply MethylMix on over 4,000 tumors across 12 cancer sites from The Cancer Genome Atlas (TCGA). MethylMix is an algorithm that produces transcriptionally predictive and differentially methylated genes in cancer that serve as potential epigenetic driver genes of malignancy and, in this manner, provides a complement to the mutation spectra being derived from DNA sequencing efforts. We applied MethylMix individually on each cancer site to identify the cancer-specific heterogeneity in the methylome; in addition, we created a pancancer methylation map by applying MethylMix on all 12 cancers sites simultaneously.

## Results

### MethylMix: a beta mixture model to identify differential and transcriptionally predictive methylation states

To identify key methylation-driven genes, we developed a model-based method called MethylMix that addresses all three criteria stated above (Figure S1 in Additional file 1). First, MethylMix uses a univariate beta mixture model to identify ‘methylation states’ for each CpG site (or cluster of correlated CpG sites), which is then associated with its nearest gene. Each methylation state is defined by a statistically similar methylation pattern across a large number of patients, removing the need for arbitrary thresholds. Second, MethylMix compares the DNA methylation of cancer with the methylation state in normal tissue to determine if a specific gene is differentially methylated in cancer. Since the normal state of DNA methylation is tissue specific, MethylMix incorporates the DNA methylation of normal tissue obtained from a subset of cancer patients in the same tissue to determine if a specific gene is hyper- or hypomethylated in that specific tissue type. Next, MethylMix produces a new metric called the ‘differential methylation value’ or ‘DM-value’ defined as the difference between the cancer methylation state and the normal methylation state. Finally, MethylMix defines the methylation state of a gene as ‘transcriptionally predictive’ if its gene expression can sufficiently be predicted by methylation of its CpG sites using a linear regression model.

### MethylMix identifies differential and transcriptionally predictive genes in 12 cancers

First we applied MethylMix individually on 12 cancer sites from TCGA: bladder cancer (BLCA), breast cancer (BRCA), colon cancer (COAD), glioblastoma (GBM), head and neck squamous carcinoma (HNSC), clear cell renal carcinoma (KIRC), acute myeloid leukemia (LAML), lung adeno carcinoma (LUAD), lung squamous carcinoma (LUSC), serous ovarian cancer (OV), rectal cancer (READ) and endometrial carcinoma (UCEC), totaling 4,291 patients. Using MethylMix we identified hyper- and hypomethylated genes, and dual genes - genes with two methylation statuses, hypermethylated in one subgroup and hypomethylated in another subgroup of patients - in a particular cancer. This resulted in between 408 and 1,133 genes called differentially and transcriptionally predictive methylated by MethylMix in each cancer (Table 1). For all cancers we identified more hypermethylated genes than hypomethylated genes. For each cancer we also found a significant number of dual genes, suggesting a dependence on the genomic context as these genes can switch from a tumor suppressor role, through hypermethylation, to an oncogene role via hypomethylation, depending on the context. Particularly for AML we identified a large number of dual genes.

**Table 1:** **Overview of the number of samples for each TCGA cancer site and the number of hyper-, hypo- and dual methylated genes as identified by MethylMix**

**TCGA cancer code**	**Number of cancer samples**	**Number of normal samples**	**Number of hypermethylated genes**	**Number of hypomethylated genes**	**Number of dual genes**
BLCA	123	6	443	74	23
BRCA	313	27	798	203	132
COAD	415	71	526	102	72
GBM	402	4	246	140	22
HNSC	310	50	728	101	42
KIRC	500	355	319	251	32
LAML	194	28	470	77	164
LUAD	430	47	576	182	39
LUSC	358	64	605	133	38
OV	584	7	234	229	66
READ	162	12	321	75	37
UCEC	500	34	618	238	77

We compared MethylMix with three previously developed methods to determine differential methylation: IMA [9], COHCAP [10] and minfi [11]. Table S1 in Additional file 2 shows a comparison of the number of hyper- and hypomethylated genes for all methods. IMA and COHCAP identify significantly more hyper- and hypomethylated genes for most cancer sites compared with MethylMix. Minfi is similar to IMA and COHAP but does not identify hypomethylated genes. Genes identified only by IMA, COHCAP or minfi were enriched with genes that are not transcriptionally predictive whereas genes uniquely identified by MethylMix were typically differentially methylated in less than 50% of the samples (Table S2 in Additional file 2). More specifically, when focusing on the transcriptionally predictive genes, MethylMix identifies 94 hyper- and 15 hypomethylated genes with a prevalence of less than 15% compared with between 3 and 5 hypermethylated genes and only 1 hypomethylated gene for IMA, COHCAP and minfi, on average, across all cancers (Table S2B in Additional file 2). For example, IMA does not identify *BRCA1* hypermethylation in breast cancer while MethylMix identifies *BRCA1* hypermethylation in 8% of breast cancer patients.

Next, we investigated for all methods the enrichment of genes with cancer driver genes identified using independent information. More specifically, we identified for each cancer site genes significantly correlated with cancer pathological stage at the gene expression level. For four cancer sites we were able to identify a sufficient number of cancer stage driver genes and showed that genes identified by MethylMix are more enriched with cancer stage driver genes compared with those identified by IMA, COHCAP and minfi (Table S3 in Additional file 2).

### Top ranked hyper- and hypomethylated genes

Next, we ranked the MethylMix genes by the prevalence of their hypo- or hypermethylation state in the 12 cancers separately for hyper- and hypomethylation and excluding dual genes (Tables S4 and S5 in Additional file 2). We identified 266 pancancer hypermethylated genes and 42 pancancer hypomethylated genes with differential methylation in at least five cancer sites. Seven genes are hypermethylated in ten cancer sites: six encoding zinc finger transcription factors (ZNF135, ZNF354C, ZNF415, ZNF542, ZNF671, ZSCAN18) and one encoding a transmembrane protein (TMEM25). The top hypomethylated gene, *MAGEA4*, is hypomethylated in nine cancer sites. We also investigated the gene expression fold change for the top ranked methylation-driven genes between the differential methylation state and the normal state (Tables S6 and S7 in Additional file 2). The top hypermethylated genes were down-regulated 3.3-fold, on average, over each of the corresponding 10 cancer sites. The top hypomethylated gene, *MAGEA4*, was up-regulated 121-fold, on average, for each of the nine cancer sites it is hypomethylated in.

### Hypermethylation suppresses differentiation

We investigated the enrichment of molecular pathways in the hyper- and hypomethylated genes in all 12 cancer sites using enrichment analysis. We specifically looked at the enrichment of stem cell gene sets based on previous reports describing epigenetic stem cell signatures in cancer [12]. We focused on stem cell gene sets that are differentially enriched in the hyper- versus hypomethylated genes and only found gene sets that are exclusively enriched in hypermethylated genes (Table S8 in Additional file 2). These genes sets are related to suppression of genes involved in differentiation, such as genes repressed by co-binding of POU5F1 (also known as OCT4), SOX2 and NANOG [13]; genes affected by knockdown of TCL1A (also known as TCL1) [14]; a polycomb target module and targets of SMAD1 and ZNF281 [15], and genes differentially expressed after RNA interference knockdown of NANOG [16].

### MethylMix identifies both known and novel methylation subtypes

**Figure 1:**
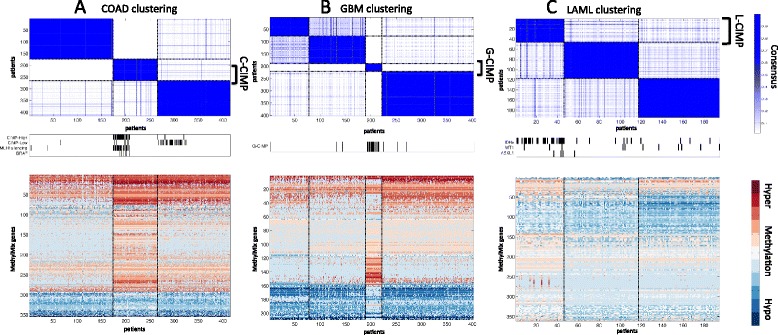
**Consensus clustering** [17] **and methylation profiles for three cancer sites with known CIMP groups. (A)** Colon cancer (COAD); **(B)** glioblastoma (GBM); **(C)** acute myeloid leukemia (LAML). Top panel: visualization of the consensus clustering with blue indicating high consensus and white indicating low consensus. Bottom panel: methylation profile with red indicating hypermethylation, white indicating normal methylation and blue indicating hypomethylation. Middle panels: COAD - CIMP-high, CIMP-low subgroups according to [19], MLH1 hypermethylation and BRAF mutation; GBM - CIMP subgroup; LAML - IDHx mutation in IDH1 or IDH2, mutation in WT1 and AXL1.

We constructed a pipeline to identify methylation-defined patient subgroups with common hyper- and hypomethylation patterns. We used consensus clustering to identify robust subgroups of patients based on DM-values [17]. The best studied methylation subgroups have been described in colorectal cancer, GBM and LAML [18]. We identified similar hypermethylated phenotypes in these cancers. In COAD, we confirmed the C-CIMP or C-CIMP-high subtype using DM-values, and its correlation with *MLH1* silencing and BRAF mutation (Figure 1A) [19]. Next, we confirmed the hypermethylated phenotype in GBM, also known as G-CIMP [20], and the hypermethylated phenotype in LAML, also known as L-CIMP characterized by IDH1 or IDH2 mutations [21] (Figure 1B,C; Figures S2, S3 and S4 in Additional file 1). Additionally, we confirmed a basal enriched methylation subtype in *BRCA* described previously, next to three other methylation subgroups (Figure S5 in Additional file 1) [22].

We also discovered that the DM-value clustering is superior to other clustering approaches. Clustering with the beta values instead of the DM-values resulted in lower intra-cluster and higher inter-cluster consensus (Table S9 in Additional file 2) and identified significantly less coherent CIMP subtypes for COAD and LAML (Table S10 in Additional file 2). Clustering using RPMM, a methylation specific clustering algorithm [23], did not result in discovery of the known CIMP groups (Figure S6 in Additional file 1; Table S11 in Additional file 2). Next, we compared the DM-value clustering with clustering of the matched gene expression data to investigate if the DM-value-derived clusters capture unique subgroups. This resulted in lower quality gene expression clusters characterized by lower intra-cluster and higher inter-cluster consensus compared with clustering DM-values for the majority of cancer sites (Table S12 in Additional file 2). Additionally, comparisons of each gene expression clustering with the corresponding DM-value clustering using the Jaccard coefficient shows low correspondence (Table S12 in Additional file 2). Qualitative analysis of the known CIMP groups for COAD, GBM and LAML show that gene expression clustering identifies clusters enriched with the COAD and GBM CIMP cluster but not the LAML CIMP cluster (Figure S7 in Additional file 1).

**Figure 2:**
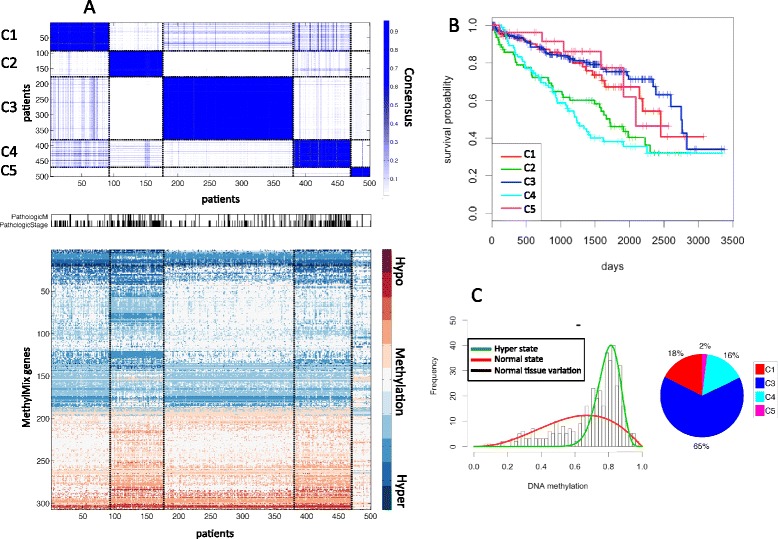
**Clear cell renal carcinoma (KIRC) methylation clustering. (A)** Consensus clustering [17] in five subgroups, correlation with pathologic M stage and binarized pathologic stage (stages 3 and 4 versus stages 1 and 2) and their corresponding methylation profiles with red indicating hypermethylation, white indicating normal methylation and blue indicating hypomethylation. **(B)** Overall survival for the five methylation subgroups. **(C)** MethylMix model for the *CYTIP* gene and distribution of *CYTIP* hypermethylation across the five KIRC subgroups.

In addition to confirming known methylation subgroups, we further identified several subgroups that have previously not been well studied or reported. We identified five methylation clusters for KIRC that have a specific methylation pattern significantly correlated with tumor stage (*P*-value <0.001; Figure 2A) and with survival (*P*-value <0.001; Figure 2B). Two clusters are enriched with high stage tumors and have poor survival. Cluster 5 is correlated with low stage tumors and also has the fewest non-zero DM-values, reflecting a normal-like KIRC tumor. The good prognosis cluster, cluster 3, is characterized by hypermethylation of CYTIP, with 65% of all hypermethylated cases in cluster 3 (Figure 2C) and virtually all samples in cluster 3 are CYTIP hypermethylated (n = 192/204). This observation is consistent with CYTIP’s role in KIRC protecting cancer cells from apoptosis signals and based on its previously shown epigenetic protective effect [24].

**Figure 3:**
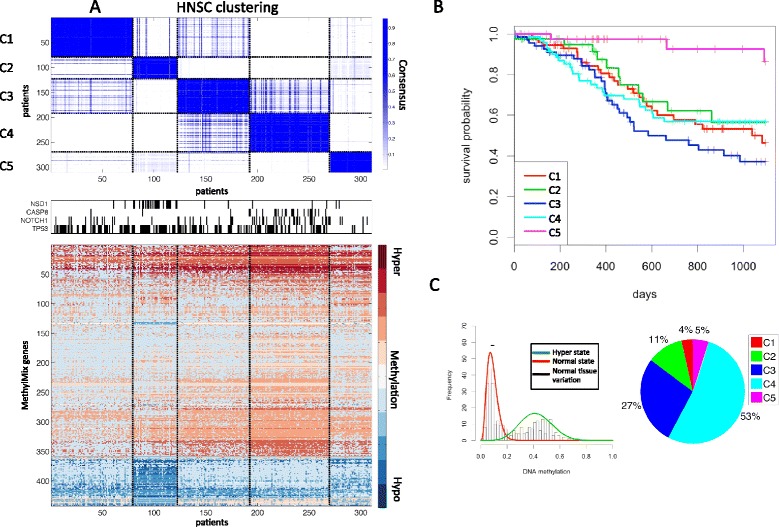
**Head and neck squamous carcinoma (HNSC) methylation clustering. (A)** Consensus clustering [17] in five subgroups, mutation status for four genes (*NSD1*, *CASP8*, *NOTCH1* and *TP53*) and their corresponding methylation profiles with red indicating hypermethylation, white indicating normal methylation and blue indicating hypomethylation. **(B)** Overall survival for the five methylation subgroups. **(C)** MethylMix model for the *BCL2* gene and distribution of *BCL2* hypermethylation across the five HNSC subgroups.

For HNSC we identified five distinct clusters significantly correlated with distinct mutational patters for each cluster (Figure 3A). Interestingly, cluster 2 was significantly associated with mutations in *NSD1* (*P*-value <0.001); more than half of the cluster 2 samples carry a mutation. Next, cluster 4 is enriched with *CASP8* and *NOTCH1* mutations (*P*-value <0.001 and <0.001, respectively), both of which have been strongly implicated in HNSC [25]. This group is also characterized by hypermethylation of *BCL2*, with more than 50% of the hyper-methylated cases being in cluster 4 (Figure 3C), and virtually all cases in cluster 4 having *BCL2* hypermethylated (that is, 75 out of 77 cases). Lastly, cluster 5, which does not have any of the key mutations present in the other clusters, showed a markedly better survival compared with the other groups (*P*-value <0.001) and was enriched with low stage tumors (*P*-value <0.001).

**Figure 4:**
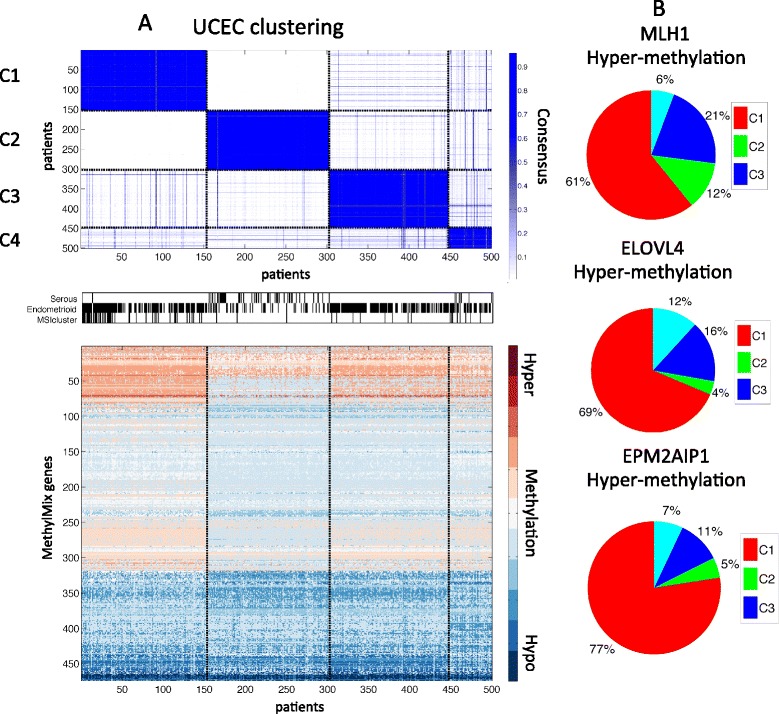
**Endometrial cancer (UCEC) methylation clustering. (A)** Consensus clustering [17] in four subgroups, corresponding histology (that is, serous or endometrioid), microsatellite instability (MSI cluster), and methylation profiles with red indicating hypermethylation, white indicating normal methylation and blue indicating hypomethylation. **(B)** Hypermethylation of *MLH1*, *ELOVL4* and *EPM2AIP1* across the four UCEC subgroups.

For UCEC we identified four methylation clusters that are correlated with histology and microsatellite instability (MSI; Figure 4A). Clusters 1 and 3 are enriched with endometrioid tumors whereas cluster 3 is a mixture of endometrioid and serous tumors (*P*-value <0.001). Cluster 1 is strongly correlated with the TCGA MSI cluster that has been proposed as a CIMP cluster in endometrial carcinoma [26]. This cluster is dominated by hypermethylation (Figure S8 in Additional file 1). Although this CIMP group is associated with *MLH1* hypermethylation, MethylMix identified other hypermethylated genes that characterize this group, such as hypermethylation of *ELOVL4* and *EPM2AIP1*, a gene sharing a promoter with *MLH1* (Figure 4B).

### Pan-cancer DNA methylation landscape

**Figure 5:**
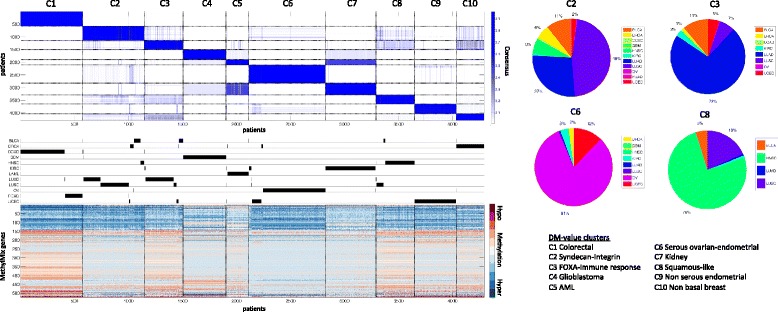
**Pancancer methylation clustering.** Left: consensus clustering [17] in 10 subgroups, corresponding cancer sites and their corresponding methylation profiles with red indicating hypermethylation, white indicating normal methylation and blue indicating hypomethylation. Right: cancer site distribution for four pancancer clusters with mixed cancer site distributions (pancancer clusters C2, C3, C6 and C8).

Next we used MethylMix to define methylation states across all 12 cancers combined. First, we identified the methylation states in all normal samples across all 12 cancer sites to select unimodal transcriptionally predictive genes and eliminate heterogeneity in the normal methylation data (Figure S9 in Additional file 1). This resulted in 1,780 genes with unimodal methylation in normal tissue. Next, we used MethylMix on the cancer samples using only these 1,780 unimodal genes and identified the pancancer DM-values. This resulted in 1,028 transcriptionally predictive genes with differential pancancer methylation states. We used consensus clustering on the corresponding DM-values of these 1,029 genes to identify pancancer clusters (Figure 5). We found 10 pancancer clusters showing significant tissue-specific enrichment (Table S13 in Additional file 2), corresponding tissue-specific correlations with mutations (Figure S10 in Additional file 1) and survival (Figure S11 in Additional file 1).

Six pancancer clusters, namely pancancer clusters 1, 4, 5, 7, 9 and 10, are tissue specific and correspond to colorectal, GBM, LAML, KIRC, UCEC and BRCA pancancer clusters, respectively. The remaining four clusters contain tumors from multiple tissues (Table S13 in Additional file 2). We used enrichment analysis of overexpressed genes to assess the commonalities that are exclusively enriched in each pancancer cluster across tissues.

A striking example is pancancer cluster 2, containing a mixture of LUAD, LUSC, BLCA, HNSC and BRCA (Figure 5). BRCA samples in this cluster are almost exclusively basal breast cancers (27 out of 32 BRCA cases in pancancer cluster 2). The lung cancer samples are not enriched in TCGA expression subtypes [27]; rather, pancancer cluster 2 is exclusively enriched in expression of collagen genes and the associated syndecan 1 and integrin pathways (Table S14 in Additional file 2), defining a syndecan-integrin signaling cluster.

Pancancer cluster 3 contains the remaining LUAD cases and a significant portion of BLCA, LUSC and UCEC. This cluster has striking enrichment of immune response genes and the FOXA1 transcriptional network (Table S14 in Additional file 2), defining a FOXA-immune response cluster. This cluster also has high expression of other parts of the integrin signaling pathway when compared with pancancer cluster 2.

Next, pancancer cluster 6 illustrates that a subset of UCEC tumors has a similar methylation pattern as the OV tumors. These UCEC samples are characterized by high stage, high grade and serous histology compared with the UCEC pancancer cluster 9 (*P*-value <0.001), defining a serous ovarian-endometrial cluster. In addition, the UCEC tumors in cluster 6 significantly overlap with the high copy number TCGA subgroup [26] (*P*-value <0.001).

Pancancer cluster 8 captures a squamous methylation pattern based on its composition of most HNSC tumors and also a subset of LUSC and BLCA tumors. The LUSC tumors are enriched with the classical TCGA subtype (*P*-value <0.001) and the BLCA tumors are enriched for only papillary tumors and are more likely to be low stage (*P*-value 0.003), together they are defining a squamous-like cluster. We also observed a significant correlation between mutations in the lincRNA ADAM6 and pancancer cluster 8 (*P*-value <0.001; Figure S10 in Additional file 1). ADAM6 mutations appear across tissue in pancancer cluster 8, including in HNSC, LUSC and BLCA cases.

Finally, we compared the DM-value pancancer clusters with previously reported pancancer clusters based on mutation, copy number data, gene expression data [28] and a meta-clustering analysis combining all of the above [29]. Comparing the DM-value pancancer clusters with the mutation and copy number pancancer clusters demonstrates the unique aspects of the DM-value pancancer clusters, with very few copy number or mutation pancancer clusters capturing the same phenomenon (Figures S12 and S13 in Additional file 1). The mutation pancancer clusters show significant overlap with the KIRC and OV pancancer clusters (Figure S12 in Additional file 1). The copy number pancancer clusters show overlap with the colorectal, GBM and again the KIRC cluster (Figure S13 in Additional file 1). None of the pancancer mutation and copy number clusters, however, identify any of the four cross-tissue pancancer clusters. Similarly, comparison with the gene expression clustering and the meta-clustering shows that both of these analyses do not capture the four pancancer clusters that we identified with DM-value clustering (Figures S14 and S15 in Additional file 1). First, both these clusterings do not identify the similarities between serous endometrial cancer and serous ovarian cancer. Next, the squamous-like pancancer cluster in both of these clusterings encompasses all squamous cancers, including lung squamous carcinoma, and does not distinguish between the lung squamous carcinoma in the syndecan-integrin signaling cluster and the pure squamous cluster. Similarly, both clusterings do not identify the difference between lung adenocarcinoma in the FOXA-immune cluster and the syndecan-integrin-squamous like lung adenocarcinoma.

## Discussion

This study represents a large analysis of DNA methylation in over 4,000 tumors and across 12 cancer sites from TCGA using a novel computational approach. DNA methylation at CpG sites is an extensively studied epigenetic mechanism driving oncogenesis. Loss or gain of CpG site DNA methylation can result in activation of oncogenes or inactivation of tumor suppressor genes. Therefore, DNA methylation is increasingly being recognized as a critical mechanism responsible for the transition from a normal to a malignant cellular phenotype [30] and a possible driver of therapeutic resistance [31], and we have shown that differentially methylated genes are potential cancer driver genes [2,32].

Our results show the existence of pancancer hypo- and hypermethylated genes. The former are potential pancancer drug targets whereas the latter have diagnostic value as potential pancancer biomarkers. Next, we found distinct methylation-driven subtypes in each cancer site. We identified between three and five clusters in each of the 12 cancer sites studied. This includes both previously studied methylation subtypes, such as the CIMP subtype [18], but also new subgroups. Applying MethylMix to the combined pancancer data set identified 10 methylation subgroups, including four clusters with significant presence of multiple tissues. Our findings emphasize the importance of studying aberrant DNA methylation in cancer and we identified surprising commonalities across cancers arising from different tissues.

Our application of MethylMix on each cancer site individually identified meaningful hypermethylated genes and also hypomethylated genes, which have not been studied extensively before. We identified several genes that are hyper- and hypomethylated in multiple cancers individually, resulting in 266 pancancer hypermethylated genes and 42 pancancer hypomethylated genes. One of the top hypermethylated genes, *TMEM25*, has been implicated in colorectal cancer [33] and is correlated with favorable prognosis in breast cancer, confirming a potential widespread tumor suppressor role for TMEM25 [34]. Similarly, the top hypomethylated gene, *MAGEA4*, is hypomethylated across nine cancers in between 18% and 60% of cases depending on the tissue. MAGEA4 is a cancer/testis antigen and a target for immunotherapy and has been identified to promote growth [35]. MAGEA4 is also a therapeutic target in breast cancer [36] where we observed hypomethylation in 18% of BRCA cases. Moreover, a family member, MAGEA3, is currently the target of a phase three clinical trial for non-small cell lung cancer [37]. This identifies aberrant methylation of *MAGEA4* as a cause of its widespread upregulation and as a potential target for immunotherapy in multiple cancers.

Previous studies on DNA methylation have focused almost exclusively on hypermethylation leading to the identification of the CIMP subgroups in at least three cancer sites, colorectal, LAML and glioma. However, hypermethylation only offers a partial view of DNA methylation and our pancancer application of MethylMix is rooted in an unbiased approach to identify methylation subtypes. This allowed us to identify not just the known CIMP subgroups but also novel subgroups that are defined by both hyper- and hypomethylation patterns. This is illustrated by the new metric that we proposed, the DM-value, reflecting differential DNA methylation with respect to normal DNA methylation status. We used the DM-value as the basis to define methylation subtypes in each of the 12 cancer sites. Our results show that the DM-value is superior to the beta value for determining subgroups (Tables S9 and S10 in Additional file 2) and outperformed a dedicated methylation clustering algorithm (Table S11 in Additional file 2). DM-value clustering also resulted in subgroups not previously described in HNSC, UCEC, and KIRC, with prognostically significant correlations for KIRC and HNSC. Moreover, we identified a subtype of HNSC that is potentially caused by NSD1 mutations. NSD1 is a SET domain histone methyltransferase that demethylates nucleosomal histone H3 lysine 36. Its mutational pattern suggests a loss of function creating aberrant histone methylation, also affecting DNA methylation processes through DNA-histone methylation crosstalk [38], and thereby potentially defining the NSD1 HNSC subtype.

Finally, we applied MethylMix across all tissues simultaneously and identified pancancer clusters. This ‘pancancer map’ illustrates the relationships between the methylation patterns in different tissues and methylation patterns common across tissues. Our map revealed six pancancer clusters that are heavily enriched in one tissue, illustrating that, due to DNA methylation being a tissue mark, aberrant DNA methylation heavily reflects the tissue of origin. Besides these homogeneous clusters, we also identified four mixed pancancer clusters. We identified two mixed clusters primarily enriched with lung cancers: a syndecan-integrin signaling cluster and a FOXA-immune response cluster (that is, pancancer clusters 2 and 3, Figure 5). The former captures a basal phenotype enriched in genes related to the integrin signaling pathway whereas the latter is exclusively enriched in immune response genes. For the remaining two mixed clusters, we discovered a serous ovarian-endometrial subgroup, creating opportunities for similar treatment of these UCEC tumors. This is consistent with the observation by TCGA that serous uterine tumors have similar gene expression patterns as serous ovarian cancers [26]. The final cluster we identified is a squamous-like pancancer cluster containing most of the HNSC cases together with a subset of classical LUSC and papillary BLCA. Both the LUSC and BLCA samples in this cluster had a tendency for better prognosis compared with the remaining samples (data not shown). We also observed an unexpected similarity between LAML cancers and KIRC cancers, although clustering in two different pancancer clusters (that is, pancancer clusters 5 and 7), they have the highest off-diagonal consensus (Figure 4). Comparison with previously reported pancancer clusters based on mutation, copy number data, gene expression and meta-clustering, combining all of the above, show the unique aspects of the methylation-based pancancer map. In addition, none of these other pancancer clusterings identified the mixed tissue pancancer clusters, such as the serous ovarian-endometrial cluster and the FOXA-immune response cluster.

By design MethylMix focuses on identifying *cis*-regulatory effects of DNA methylation on gene expression and does not currently model *trans*-regulatory effects. Further studies are needed to tackle the multiple testing challenge of identifying *trans*-regulatory DNA methylation effects. Additionally, we focused on gene specific hyper- and hypomethylation as opposed to identifying regional or genome scale DNA methylation patterns as shown by other studies [39]. This choice was motivated based on the properties of the TCGA DNA methylation platforms that focus primarily on identifying promoter DNA methylation and warrant gene-specific study complementary to previous work.

## Conclusions

Our analysis is far from complete but as more tumor types are completed by TCGA a more comprehensive picture will emerge identifying more cross-tissue methylation patterns. Identifying commonalities across cancers originating from different tissues can help to move away from a paradigm based on treating cancers based on anatomy to one based on treatment based on common DNA methylation patterns.

## Materials and methods

We developed MethylMix, a novel multi-step model-based algorithm to determine significant hypo- and hypermethylated transcriptionally predictive genes in cancer (Figure S1 in Additional file 1).

### The Cancer Genome Atlas pancancer data

We used level three normalized pancancer data from TCGA for 12 tissues: BLCA, BRCA, COAD, HNSC, LAML, KIRC, LUAD, LUSC, GBM, READ, UCEC and OV [40,41]. We used all available DNA methylation and RNA-Seq gene expression data from TCGA PAN Cancer Freeze 4.7 available through synapse [42].

### Preprocessing DNA methylation data

The DNA methylation data in TCGA was generated using the Illumina Infinium HumanMethylation 27 k or 450 k BeadChip. DNA methylation was quantified using beta values ranging from 0 to 1, with values close to 0 indicating low levels of DNA methylation and close to 1 high levels of DNA methylation. We removed CpG sites with more than 10% missing values in all samples. We used the 15-K Nearest Neighbor (KNN) algorithm to estimate the remaining missing values in the data set [43]. For cancer sites that had both 27 k and 450 k data, the overlapping probes between both data sets were used. For all other data sets, all 27 k or all 450 k probes were used. Due to the size of TCGA, TCGA samples were analyzed in batches and a significant batch effect was observed based on a one-way analysis of variance. We applied Combat to adjust for these effects [44]. This procedure was performed for all primary tumor samples and normal solid tissue. For GBM, four normal samples were obtained from [20,45].

The 27 k and 450 k DNA methylation platforms have multiple CpG sites per gene, thereby requiring a method to collapse the data of multiple CpG sites to assess gene-specific DNA methylation. Because averaging all CpG sites can remove signal from the data, we used a dimensionality reduction method to group correlated probes and reduce redundancy. To accomplish this, we used hierarchical clustering with average linkage in combination with Pearson correlation. This cluster algorithm groups CpG sites based on a minimum correlation and keeps them separate when they do not satisfy this minimum correlation threshold. First, the average linkage hierarchical clustering algorithm was used to cluster all probes of a single gene into CpG clusters. Then we cut off the hierarchical tree at a Pearson correlation threshold of 0.4 to define CpG site clusters and single CpG sites when they do not correlate with other sites, resulting in potentially multiple CpG site clusters representing a single gene.

### Preprocessing gene expression data

RNA-seq gene expression data were available for most primary tumor samples. We log-transformed the RNA-seq counts and replaced infinities with a low value. Missing values were estimated similarly as for the DNA methylation data using 15-KNN [46]. Batch correction was done using Combat [44].

### MethylMix: identifying transcriptionally predictive and differentially methylated genes in cancer

MethylMix identifies transcriptionally predictive and differentially methylated genes in cancer via a three-step algorithm, depicted in Figure 1, and described in detail below.

#### Step 1: identifying the methylation state of each CpG site using univariate beta mixture models

After preprocessing, the methylation data are represented by ratios bounded between 0 and 1 representing the proportion of methylated signal versus total signal. These proportions or beta values are beta-distributed and we applied beta mixture modeling to identify subpopulations of patients with similar DNA methylation levels for each gene using only the cancer methylation data [47]. Next, for each CpG site, we used a stepwise approach to determine the minimum number of mixture components that best fit the patient data. We use the Bayesian Information Criterion (BIC) for model selection and to avoid overfitting:

$$ -2 \times \log \left(\mathrm{L}\right) + \mathrm{k} \times \log \left(\mathrm{N}\right) $$

where k is the number of parameters of the univariate beta mixture model, L is the likelihood and N is the number of data points. BIC is more conservative than the similar Akaike Information Criterion because it penalizes the free parameters more. This process involves iteratively adding a new mixture component if the BIC improves. This procedure is repeated for each CpG site or CpG cluster and results in a parameterized model of a mixture of beta distributions. For a CpG site, each beta mixture represents a subset of patients for whom a particular beta distribution of DNA methylation states are observed.

#### Step 2: defining hyper- and hypomethylated cancer genes relative to normal

To determine if a specific CpG cluster is hypo- or hypermethylated in cancer, we compare its methylation level with the DNA methylation levels of normal tissue samples. We compare the mean of each of the mixture components of each CpG site with the average methylation of its counterpart in the normal samples. We use a Wilcoxon rank sum test to determine a significant difference based on a significant Q-value of 0.05 calculated using *P*-value multiple testing correction with false discovery rate (FDR). In addition, we require a minimum difference of 0.10 based on the platform sensitivity reported in [48].

#### Step 3: identifying transcriptionally predictive methylation

MethylMix requires that the DNA methylation level of a gene has a significant effect on its corresponding gene expression to be considered a methylation-driven gene. We used linear regression to model the expression of each gene in terms of its own DNA methylation. The performance of the model was estimated using the R-square statistic on the unseen data in each cross-validation loop. For subsequent enrichment and clustering analyses we used a *P*-value threshold of 0.001 and an R-square of at least 0.10 and required a negative correlation between methylation and matched gene expression.

### Comparison of MethylMix with IMA, COHCAP and minfi

We compared MethylMix with three previously published methods: IMA [9], COHCAP [10] and minfi [11].

IMA is conceptually based on a statistical test comparing the methylation values of a CpG site between cancer and normal. We used IMA with the default Wilcoxon rank sum test to determine statistical significance with the same thresholds as for MethylMix, namely a *P*-value threshold of 0.01 and a minimum difference of the beta value between cancer and normal of 0.1 based on the platform sensitivity [48].

COHCAP combines several steps of methylation modeling and also includes a differential methylation step. COHCAP discretizes the methylation data based on user-specified thresholds and uses a discrete test. We used COHCAP without the default cutoffs as this resulted in very few genes being called differentially methylated for many cancer sites, and set the methylation and unmethylation cutoffs to 0 and 1, respectively. We used a delta cutoff of 0.1 to be consistent with MethylMix.

Minfi is a pipeline that combines several steps in the analysis of methylation data. This includes a differential methylation step based on an F-test. We again used a Q-value threshold of 0.05 and included a methylation difference filter of 0.1 to be consistent with all other methods. Minfi does not identify differential hypomethylation.

We compared IMA, COHCAP and minfi with MethylMix by investigating their enrichment with cancer driver genes. We used correlation of gene expression with cancer stage to identify potential cancer driver genes using independent information. We used the spearman correlation test to identify genes significantly correlated with cancer stage. We corrected for multiple testing using the FDR [49] and selected only genes with Q-value <0.1. Next, we investigated the intersection between hypermethylated genes and genes negatively correlated with cancer stage (that is, putative tumor suppressor genes), and the intersection between hypomethylated genes and genes positively correlated with cancer stage (that is, putative oncogenes). We compared the numbers for IMA, COHCAP, minfi and MethylMix to identify the potential of each method to find cancer stage driver genes. Next, we only focused on cancer sites for which at least 200 significant genes could be identified. Three cancer sites do not have relevant cancer stage information, GBM (only advanced stage), LAML (different cancer stage classification) and ovarian cancer (only advanced stage).

### Identifying patient subgroups based on differential methylation values

Similar to copy number data analysis, the mixture model feature of MethylMix allows us to generate a ‘differential’ DNA methylation value by clustering each DNA methylation measurement to its nearest mixture component. We represent each sample by its differential methylation value, or DM-value, defined as the difference of the mean of the mixture component it clusters in and the mean of normal DNA methylation. This essentially creates a differentially methylated data set. Next, we clustered the DM-values using consensus clustering for each cancer and compared with known methylation subgroups. This analysis was limited to mixture components that have significantly different DNA methylation compared with normal and thus focused only on aberrantly methylated CpG sites or clusters. We compared clustering of the DM-values with clustering of the beta values to identify the benefit of using methylation states compared with beta values for identifying patient subgroups. We used consensus clustering as a clustering algorithm and also compared with RPMM [23], a dedicated clustering algorithm for DNA methylation data (see the ‘Clustering analysis’ section below).

### Pancancer MethylMix analysis

To create a pancancer methylation map across all tissues, we used the following workflow (Figure S9 in Additional file 1). First, we used MethylMix on the combined methylation data of all normal tissue samples. Next, we selected all genes that only have one beta distribution in the mixture model. These genes are defined as unimodal genes in the normal DNA methylation data. Next we intersected this list with genes that are transcriptionally predictive in the pancancer analysis based on a significant negative correlation between DNA methylation and gene expression in the combined pancancer DNA methylation and gene expression data. Then we applied MethylMix on all the cancer samples using the unimodal transcriptionally predictive genes only. This resulted in a matrix with the pancancer DM-values for all samples. This matrix was further analyzed using consensus clustering (see the ‘Clustering analysis’ section below).

### Clustering analysis

We used consensus clustering [17] to identify robust clusters defined by DM-values within each tissue and across all cancers (see the ‘Pancancer MethylMix analysis’ section above). For the cluster analysis within each tissue we used the following parameters: maximum nr of clusters = 10, number of subsamples = 1,000 with 0.8 the proportion of the subsample and we used the k-means cluster algorithm with Euclidean distance. For clustering of the pancancer DM-values we used the same consensus cluster parameters except we investigated up to 20 clusters. Next, we used PAM analysis to identify the centroids for each cluster [50] and SAM analysis to identify differentially expressed genes for each cluster [51]. For SAM analysis we used the Wilcoxon rank sum test and 100 permutations. SAM differentially expressed genes were analyzed with gene set enrichment analysis (see the ‘Gene set enrichment analysis’ section below).

We compared consensus clustering with a dedicated cluster algorithm for methylation data called RPMM [23]. RPMM is a model-based recursive-partitioning clustering algorithm that specifically models the beta values. We used RPMM on the genes identified by MethylMix corresponding to both differential and transcriptionally predictive genes. In addition we also ran RPMM on the top 25% most varying genes based on their beta value methylation profiles. Using default RPMM parameters resulted in an impractically high number of between 50 and 100 clusters identified for individual cancer sites. Therefore, we restricted the maximum level of the hierarchical tree to three, resulting in a maximum of nine clusters, consistent with our consensus clustering default parameters.

### Gene set enrichment analysis

To evaluate the gene set enrichment of hyper- and hypomethylated gene, we used several databases, namely MSigDB version 3 [52], GeneSetDB version 4 [53], CHEA for CHIP-X gene sets version 2 [54] and manually curated gene sets related to stem cells and immune gene sets. We used a hypergeometric test to check for enrichment of gene sets in gene lists. We corrected for multiple testing using the FDR [49]. We used Fisher’s method to combine *P*-values of the pathway enrichment for all 12 cancers.

We used the gene set enrichment analysis to identify unique enrichment to characterize the pancancer clusters. For each cluster we used SAM to identify the up-regulated genes for each cluster and then used the gene set enrichment procedure to investigate the enrichment of the gene list databases. Next, we only looked at the gene sets that are uniquely enriched in each pancancer cluster with the following cutoffs: *P*-value <0.0001, Q-value <0.05, SAM fold change >1.

### Survival analysis

We used Cox proportional hazards modeling to investigate univariate relationships between DM-values and survival (survival R package v.2.36-10). Hazard ratios were used to report the direction of the survival effect and the Wald test was used to determine significance of Cox models. We used Kaplan-Meier survival curves to visualize survival relationships. We used multiple testing correction with FDR to correct for multiple testing and calculate Q-values [49]. We focused on genes with Q-value <15%.

### Software availability

MethylMix was implemented as an R package [55] and is available at [56] or through bioconductor at [57].

## Additional files

Additional file 1:
**Supplementary figures: compilation of all supplementary figures visualizing supporting data.**
Additional file 2:
**Supplementary tables: compilation of all supplementary tables containing supporting data.**

## Abbreviations

BIC: Bayesian Information Criterion
BLCA: bladder cancer
BRCA: breast cancer
COAD: colon cancer
DM: differential methylation
FDR: false discovery rate
GBM: glioblastoma
HNSC: head and neck squamous carcinoma
KIRC: clear cell renal carcinoma
LAML: acute myeloid leukemia
LUAD: lung adeno carcinoma
LUSC: lung squamous carcinoma
MSI: microsatellite instability
OV: serous ovarian cancer
READ: rectal cancer
TCGA: The Cancer Genome Atlas
UCEC: endometrial carcinoma
