# Supplementary material for: Pancancer analysis of DNA methylation-driven genes using MethylMix
Source: Genome Biol. 2015 Jan 29;16(1):17. doi: 10.1186/s13059-014-0579-8 (PMC4365533; doi:10.1186/s13059-014-0579-8)

Additional File 1

Supplementary Figure legends

Supplementary Figure 1: Workflow of MethylMix. The steps (i), (ii) and (iii) refer to the different steps of MethylMix to transform the raw methylation data into transcriptionally predictive and differentially methylated genes. Panels (A) through (D) illustrate how steps (i) thru (iii) affect the methylation data.

Supplementary Figure 2: PAM model centroids for the colon cancer (COAD) methylation subgroups showing extensive hypermethylation for subgroup 2, the COAD-CIMP methylation subgroup, based on positive weights for the majority of genes. Only the top 100 genes are shown.

Supplementary Figure 3: PAM model centroids for the glioblastoma (GBM) methylation subgroups showing extensive hypermethylation for subgroup 3, the GBM-CIMP methylation subgroup, based on positive weights for the majority of genes. Only the top 100 genes are shown.

Supplementary Figure 4: PAM model centroids for the acute myeloid leukemia (LAML) methylation subgroups showing extensive hypermethylation for subgroup 1, the LAML-CIMP methylation subgroup, based on positive weights for the majority of genes. Only the top 100 genes are shown.

Supplementary Figure 5: Breast cancer (BRCA) methylation clustering. Top panel: consensus clustering in five subgroup, middle panel: correlation with the intrinsic subtypes, bottom panel: corresponding methylation profiles with red=hypermethylation, white=normal methylation and blue=hypomethylation.

Supplementary Figure 6: Comparison between DM-value clustering and beta-value clustering for COAD, LAML and GBM. Top panel: DM-value clusters for each cancer site. Top middle panel: gold standard clinical data for CIMP clusters for each cancer site. Bottom middle panel: RPMM clustering based on the beta-values. CIMP cluster comparison has been highlighted with gray transparent boxes showing heterogeneity for the CIMP clusters in the RPMM clustering. Bottom panel: corresponding methylation profiles with red=hypermethylation, white=normal methylation and blue=hypomethylation.

Supplementary Figure 7: Comparison between DM-value clustering and gene expression clustering for COAD, LAML and GBM. Top panel: DM-value clusters for each cancer site. The CIMP clusters has been indicated at the top. Middle panel: gene expression clustering based. Bottom panel: corresponding methylation profiles with red=hypermethylation, white=normal methylation and blue=hypomethylation.

Supplementary Figure 8: PAM model centroids for the endometrial carcinoma (UCEC) methylation subgroups showing extensive hypermethylation for subgroup 1, a putative UCEC-CIMP methylation subgroup, based on positive weights for the majority of genes. Only the top 100 genes are shown.

Supplementary Figure 9: Workflow of the MethylMix application to pancancer data. First MethylMix is applied on all normal samples combined to identify genes that are unimodal. Next, MethylMix is run on the combined cancer methylation data only on unimodal genes. This results in pancancer methylation states and their associated DM values.

Supplementary Figure 10: Pancancer methylation clustering with association to the top 30 mutated genes after removing 115 long genes (>10kb). Top panel: consensus clustering in ten subgroups, middle panel mutation status for the top 30 mutated genes. Bottom panel: corresponding methylation profiles with red=hypermethylation, white=normal methylation and blue=hypomethylation.

Supplementary Figure 11: Pancancer methylation clustering survival analysis. Overall survival for all pancancer clusters in days.

Supplementary Figure 12: Comparison between the pancancer DM-value methylation clustering and a pancancer mutation clustering. Top panel: consensus clustering in ten subgroups, bottom panel TCGA mutation based pancancer clusters.

Supplementary Figure 13: Comparison between the pancancer DM-value methylation clustering and a pancancer copy number clustering. Top panel: consensus clustering in ten subgroups, bottom panel TCGA copy number based pancancer clusters.

Supplementary Figure 14: Comparison between the pancancer DM-value methylation clustering and a pancancer gene expression clustering. Top panel: consensus clustering in ten subgroups, bottom panel TCGA gene expression clustering based pancancer clusters. The four mixed tissue pancancer clusters are highlighted to show the lack of correspondence between these clusters and the gene expression clustering.

Supplementary Figure 15: Comparison between the pancancer DM-value methylation clustering and a pancancer meta-clustering. Top panel: consensus clustering in ten subgroups, bottom panel TCGA meta clustering based pancancer clusters. The four mixed tissue pancancer clusters are highlighted to show the lack of correspondence between these clusters and the meta clustering.

Supplementary Figure 1

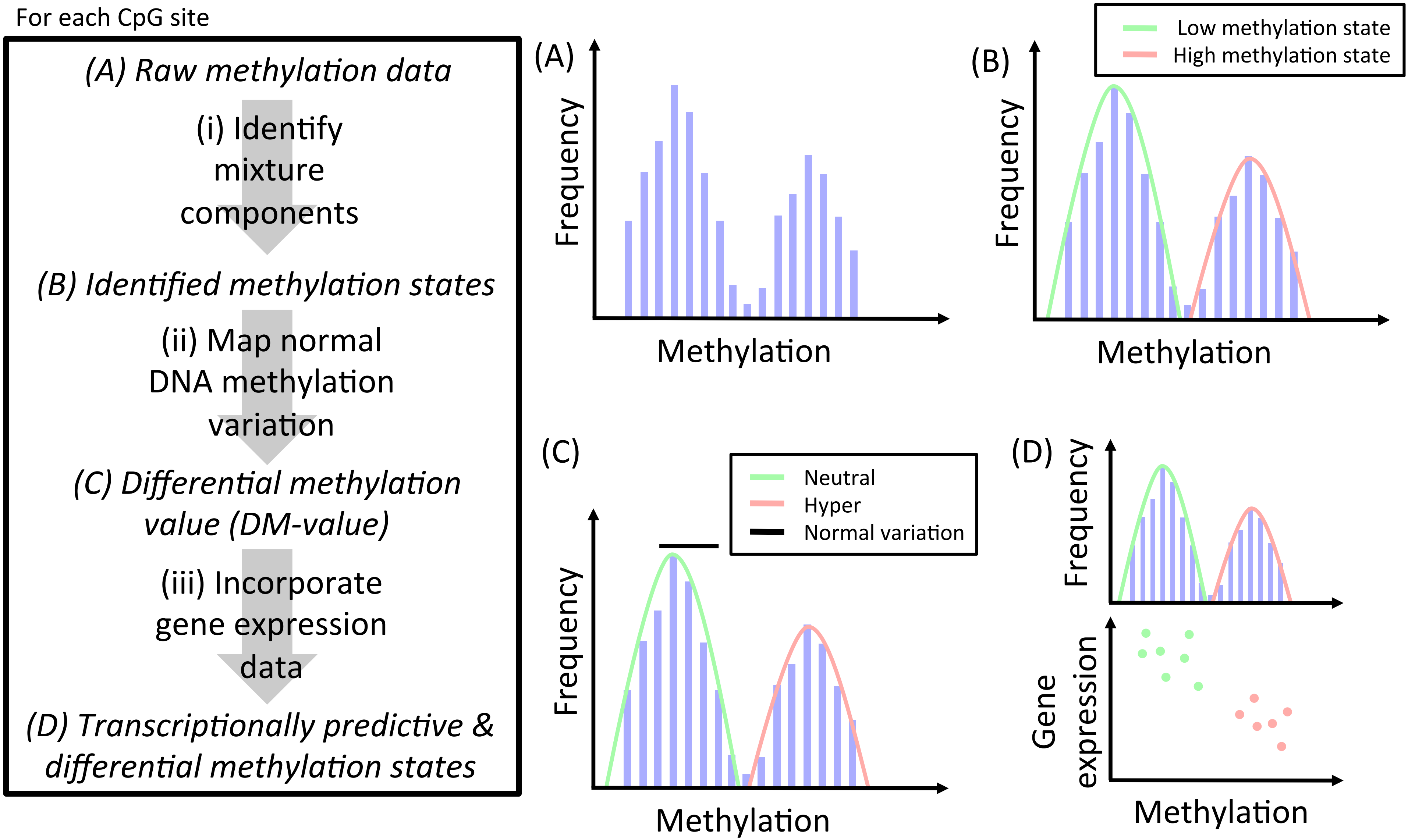

Supplementary Figure 2

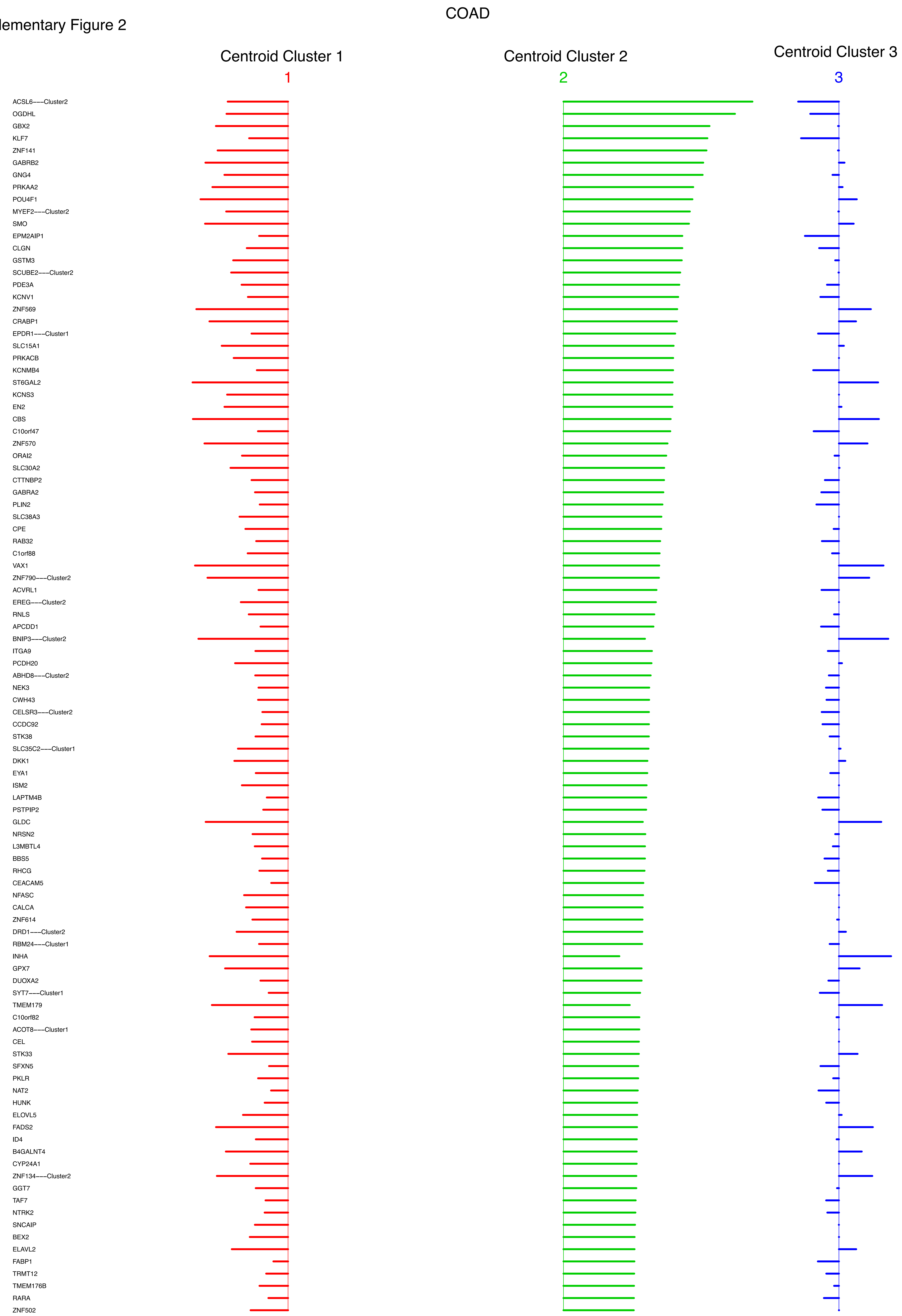

Supplementary Figure 3

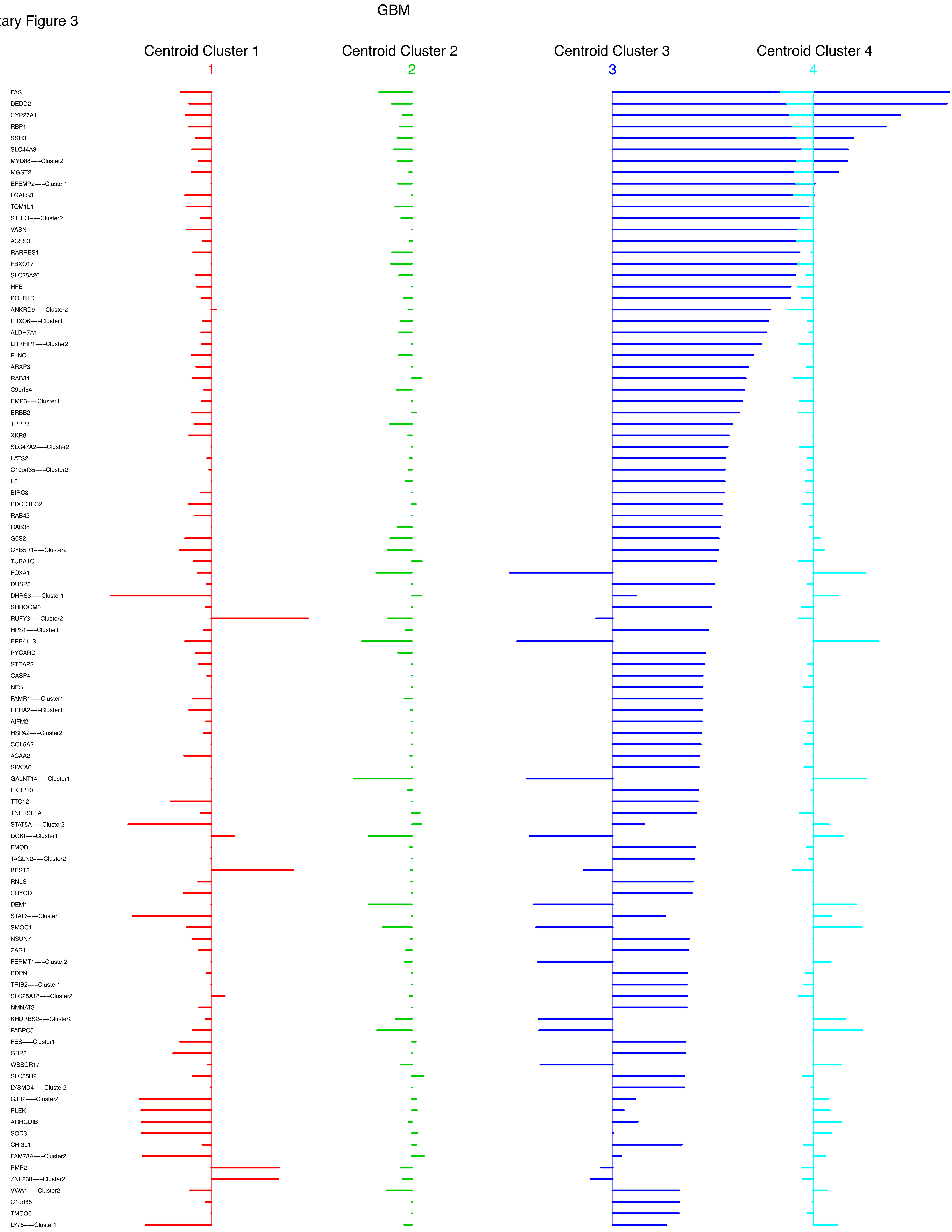

Supplementary Figure 4

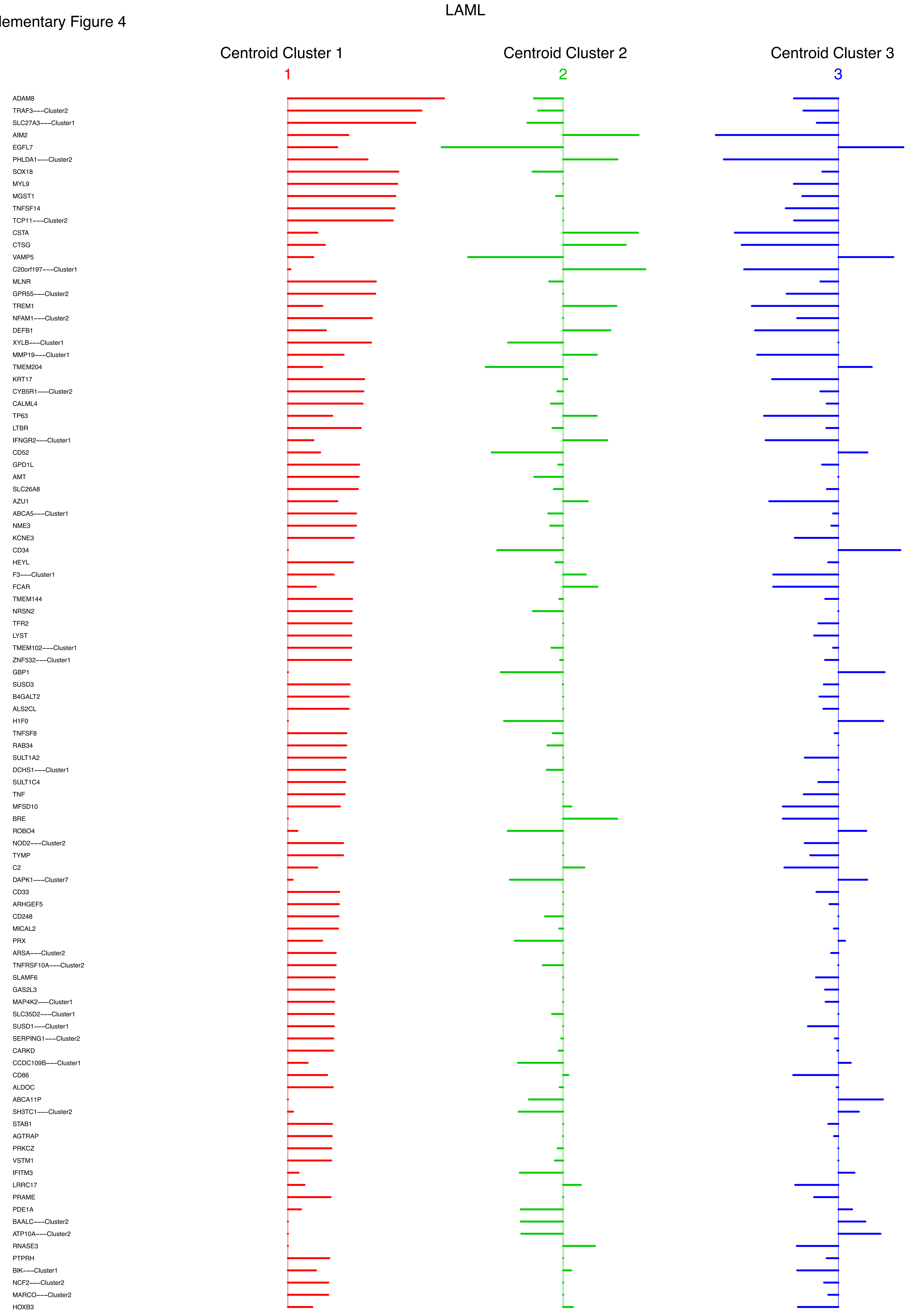

Supplementary Figure 5

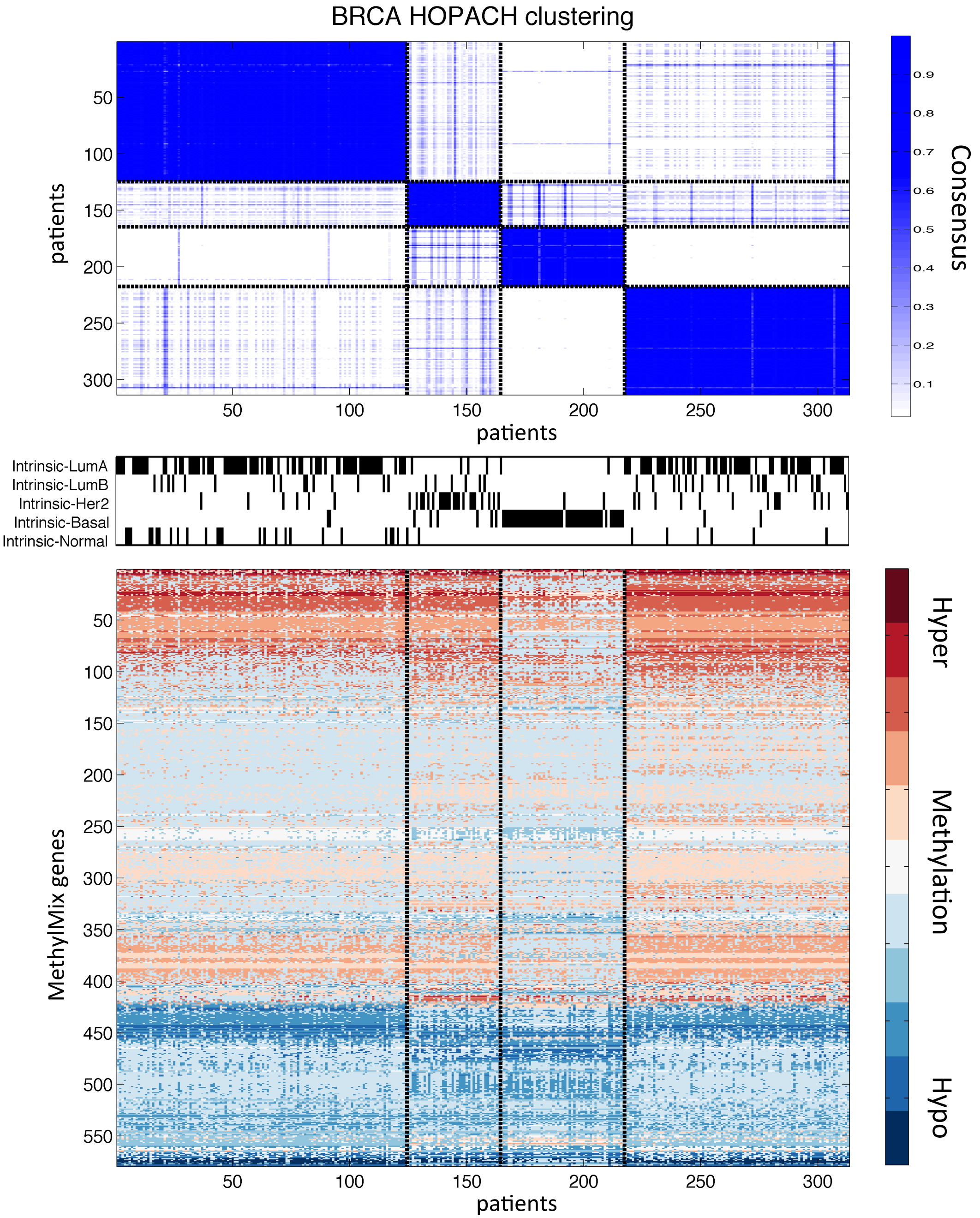

Supplementary Figure 6

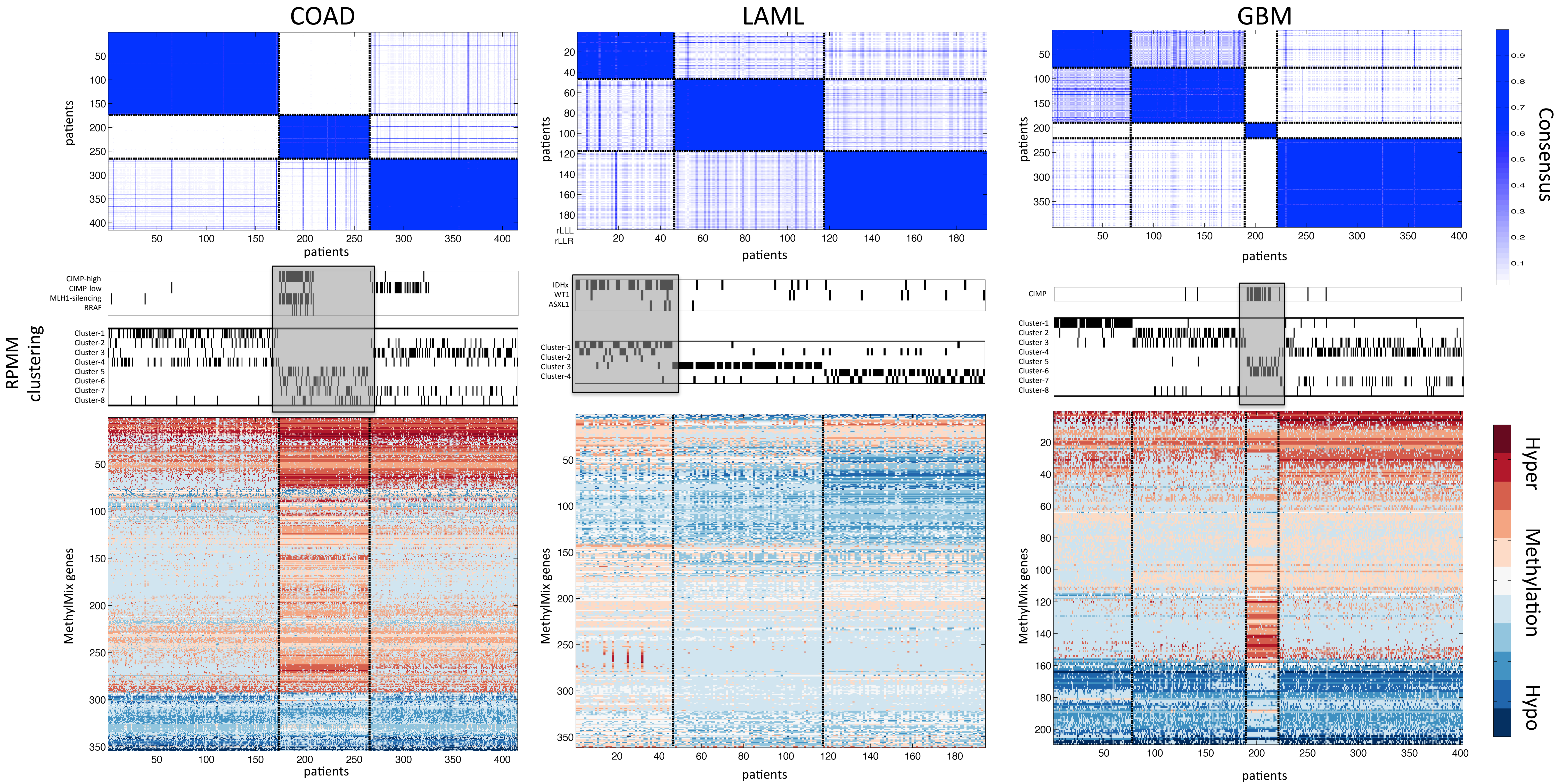

Supplementary Figure 7

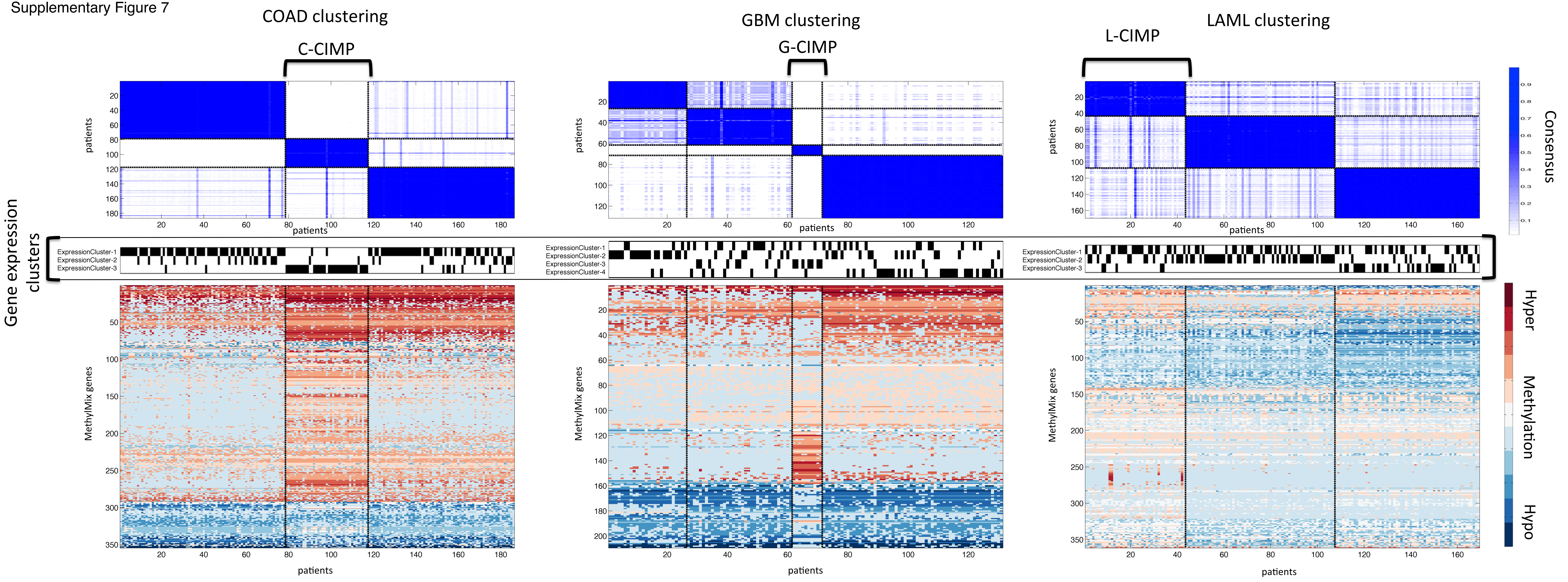

Supplementary Figure 8

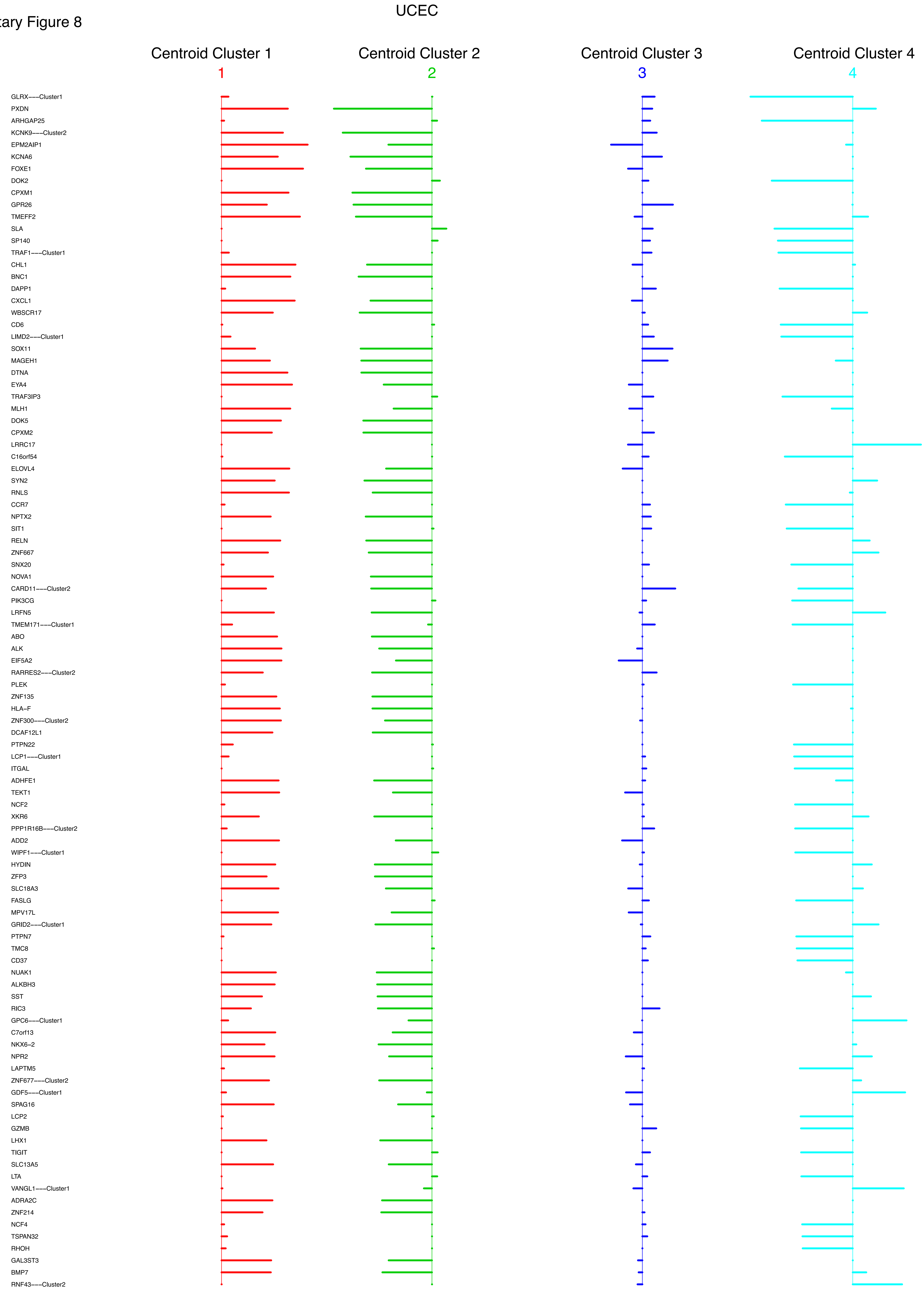

Supplementary Figure 9

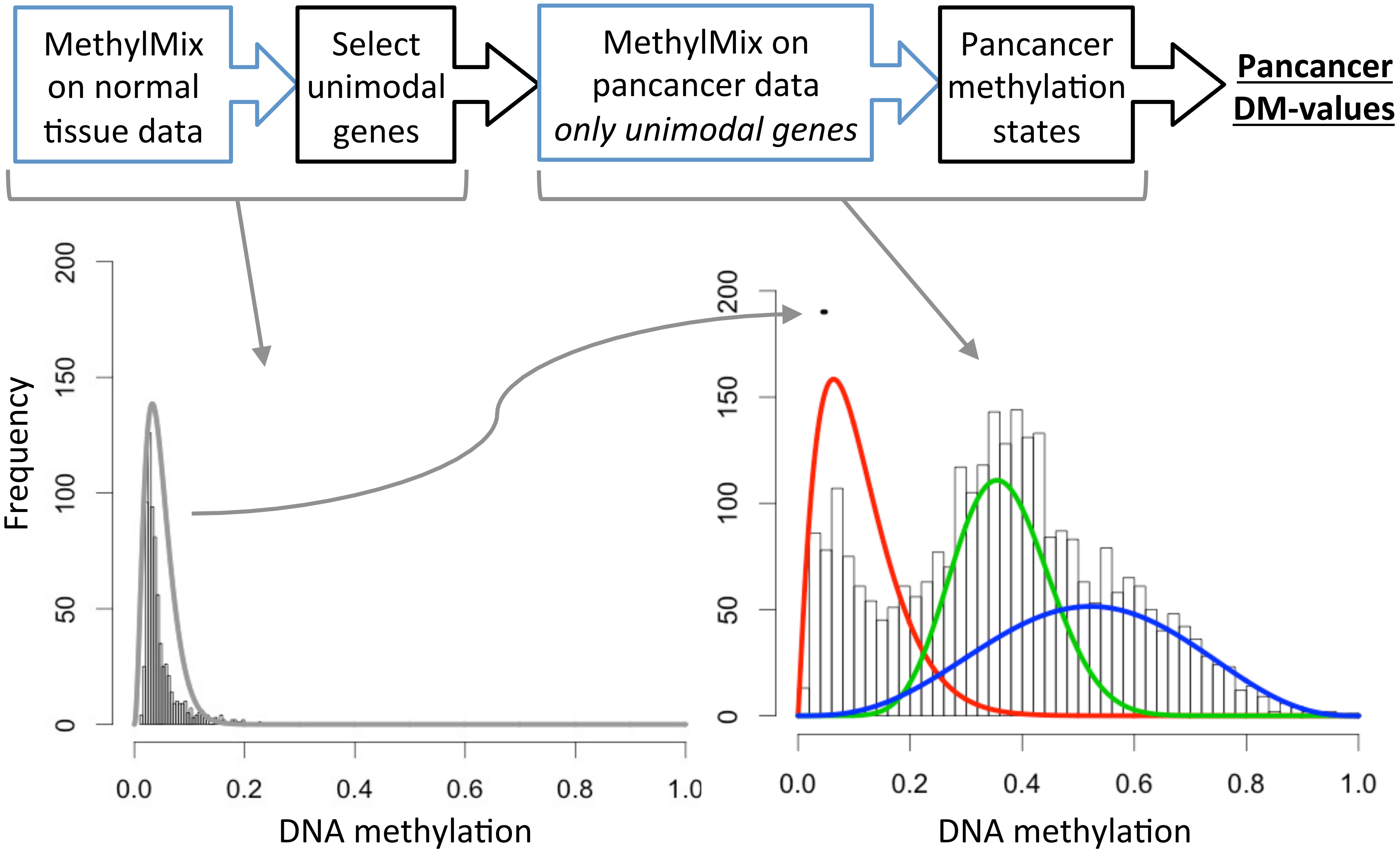

Supplementary Figure 10

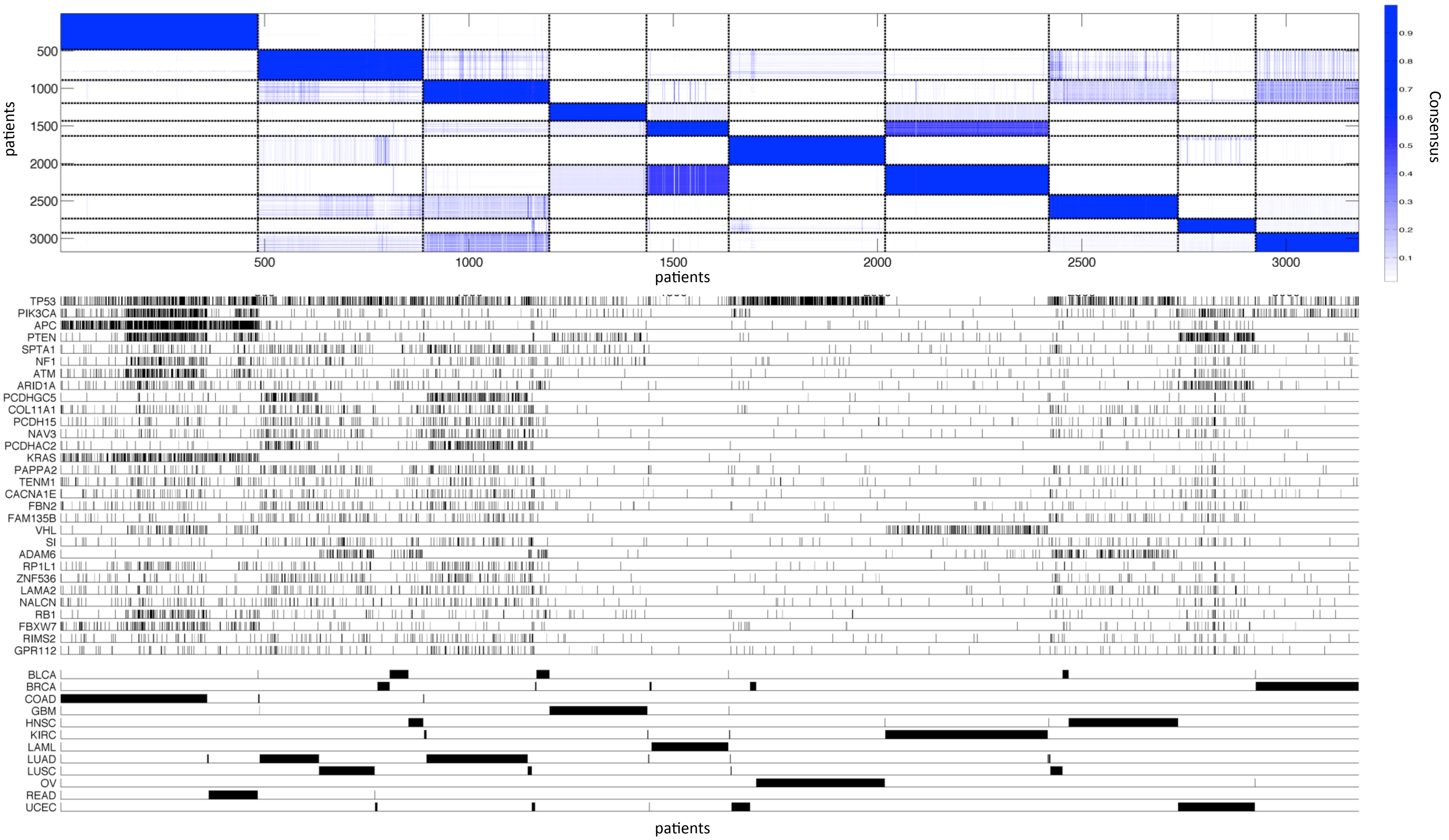

Supplementary Figure 11

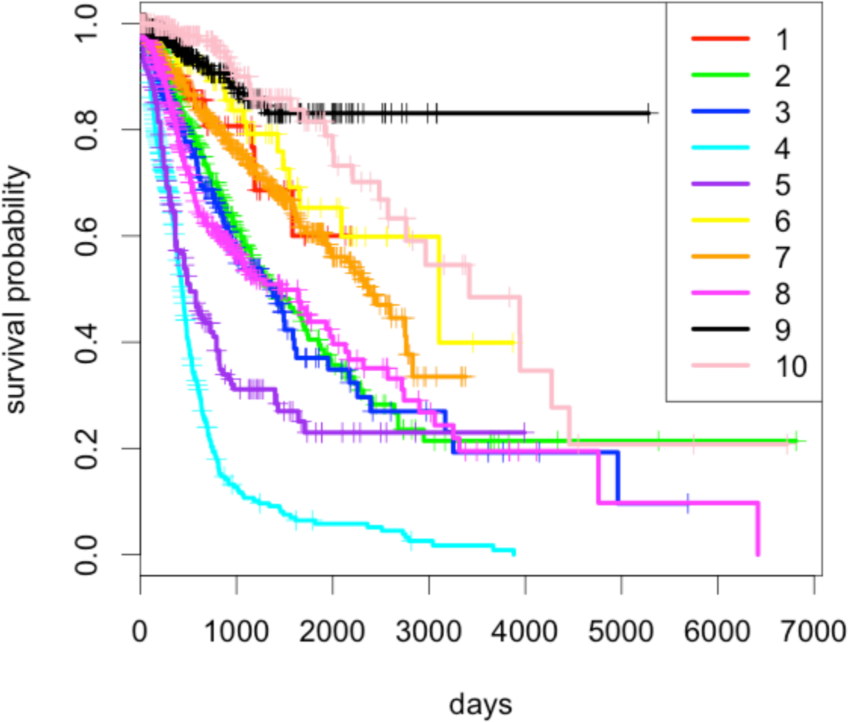

Supplementary Figure 12

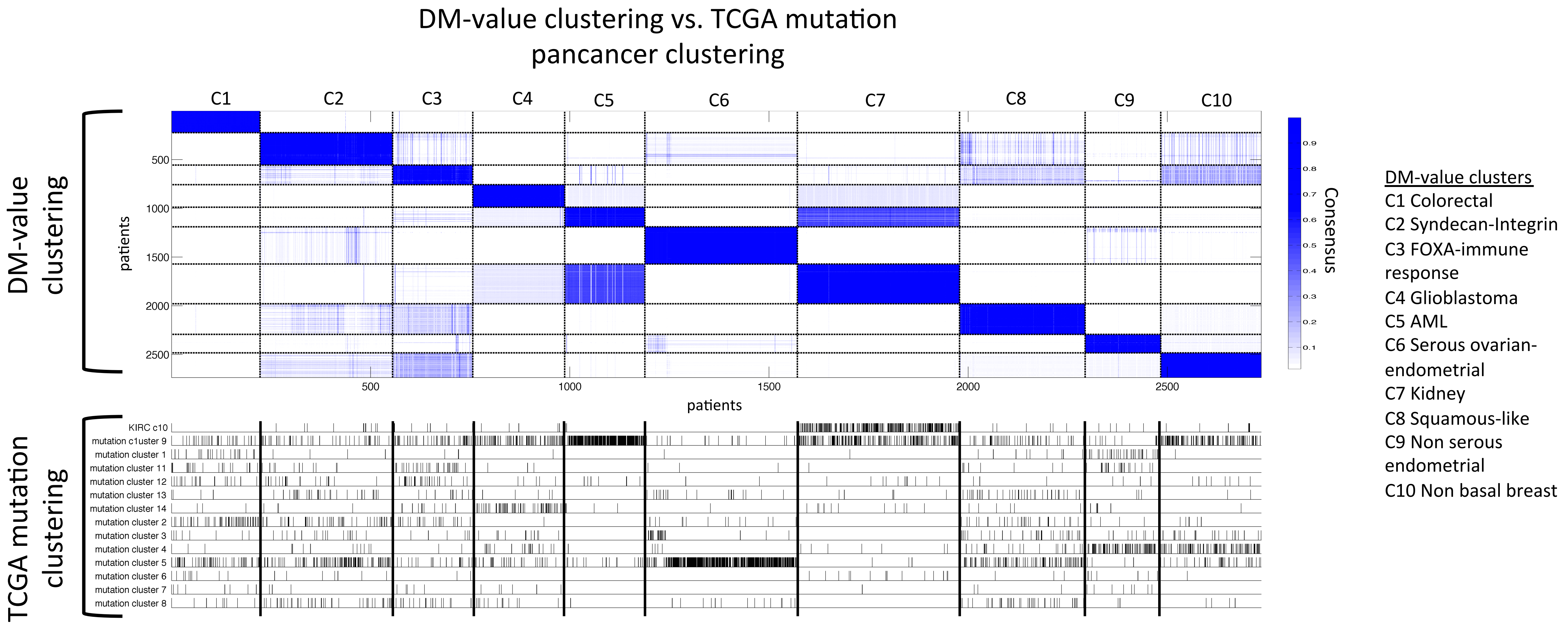

Supplementary Figure 13

DM-value clustering vs. TCGA copy number pancancer clustering

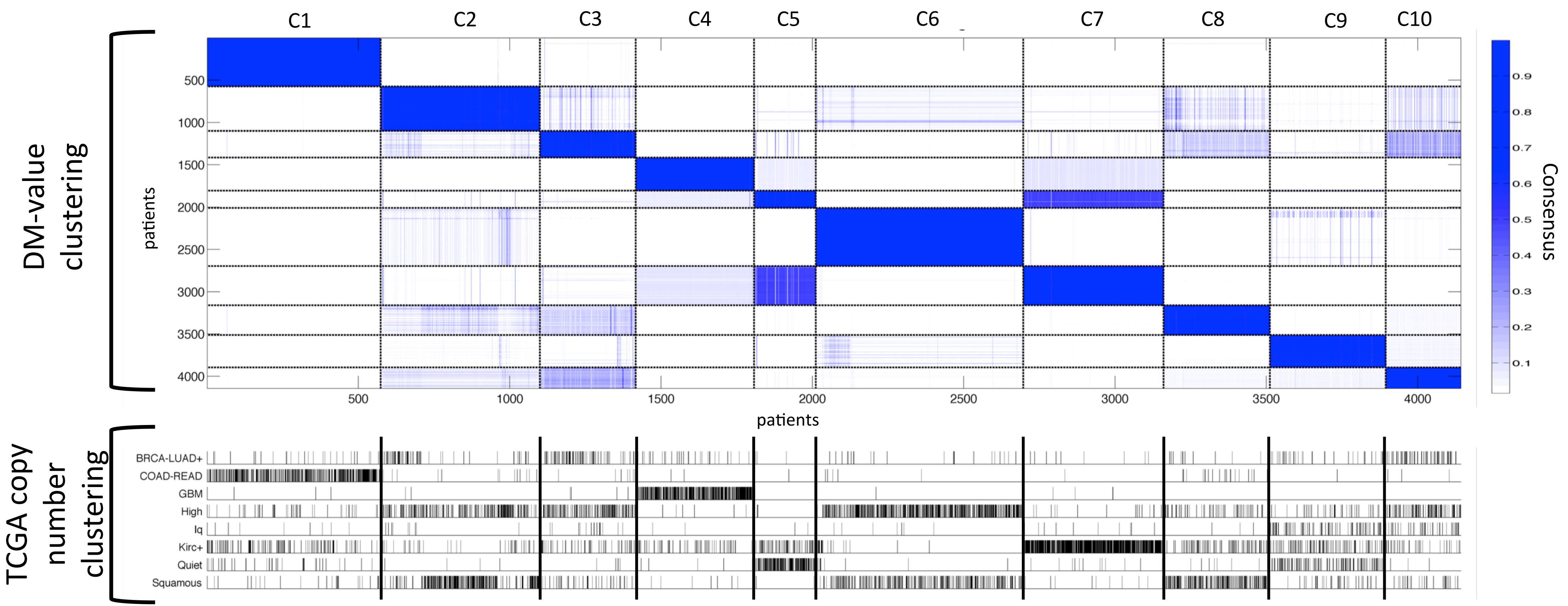

DM-value clusters

C1 Colorectal
C2 Syndecan-Integrin
C3 FOXA-immune response
C4 Glioblastoma
C5 AML
C6 Serous ovarian-endometrial
C7 Kidney
C8 Squamous-like
C9 Non serous endometrial
C10 Non basal breast

Supplementary Figure 14

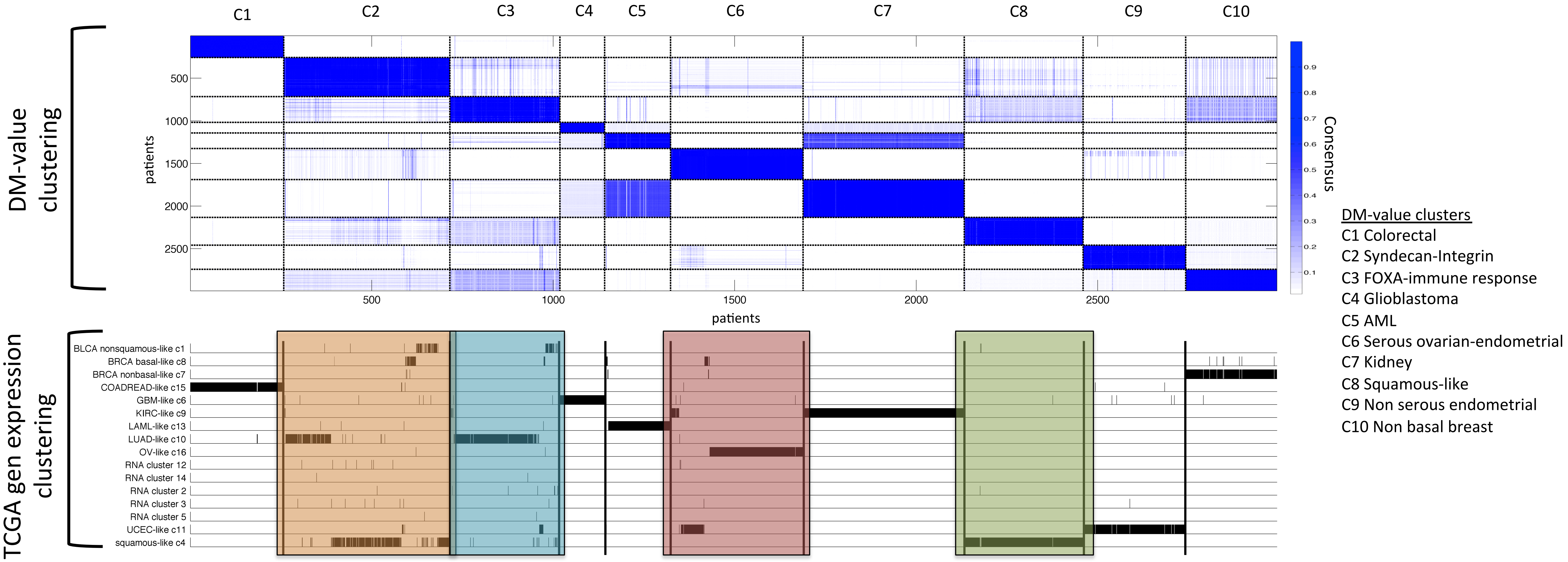

Supplementary Figure 15

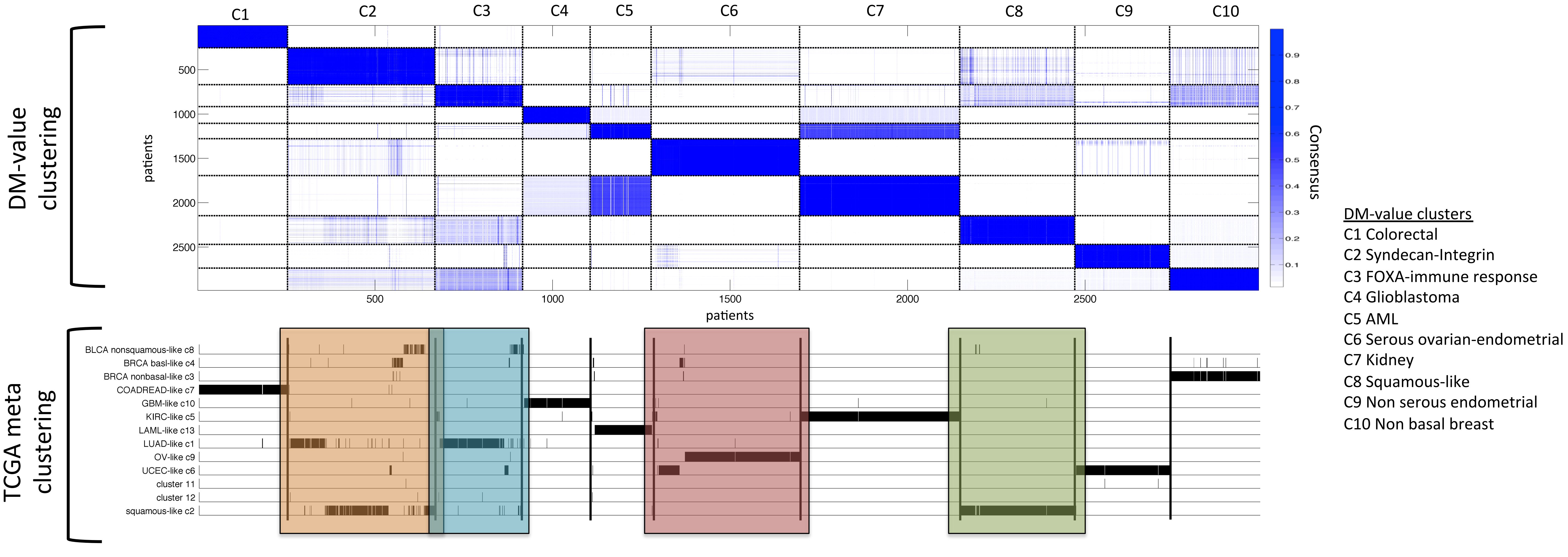

Supplement: Additional file 1: — Supplementary figures: compilation of all supplementary figures visualizing supporting data. [file 13059_2014_579_MOESM1_ESM.pdf]
