# Supplementary material for: Pancancer analysis of DNA methylation-driven genes using MethylMix
Source: Genome Biol. 2015 Jan 29;16(1):17. doi: 10.1186/s13059-014-0579-8 (PMC4365533; doi:10.1186/s13059-014-0579-8)
Supplement: Additional file 2: — Supplementary tables: compilation of all supplementary tables containing supporting data. [file 13059_2014_579_MOESM2_ESM.pdf]

Additional File 2

Supplementary Table Legends

Supplementary Table 1: Comparison of MethylMix with IMA by number of hyper and hypomethylated genes for each method.

Supplementary Table 2: Prevalence of hyper and hypomethylated genes exclusively identified by MethylMix when compared with IMA, and overlap of the genes exclusively identified by IMA with transcriptionally predictive genes when compared with MethylMix.

Supplementary Table 3: Identification of cancer driver genes based on correlation with pathological cancer stage and comparison of the MethylMix and IMA hyper and hypomethylated genes based on intersection with these cancer driver genes.

Supplementary Table 4: Prevalence of the top 100 hypermethylated genes across twelve cancer sites represented as the percentage of samples that have a hypermethylation for each gene.

Supplementary Table 5: Prevalence of the top 100 hypomethylated genes across twelve cancer sites represented as the percentage of samples that have a hypomethylation for each gene.

Supplementary Table 6: Gene expression fold change for the top 100 hypermethylated genes across twelve cancer sites.

Supplementary Table 7: Gene expression fold change for the top 100 hypomethylated genes across twelve cancer sites.

Supplementary Table 8: Enrichment of stem cell pathways across twelve cancer sites for hypermethylated genes.

Supplementary Table 9: Comparison between DM-value clustering and beta-value clustering based on the intra-cluster and inter-cluster consensus, and using the Jaccard coefficient for each cancer site.

Supplementary Table 10: Comparison of the known CIMP subtypes for COAD, LAML and GBM between the DM-value clustering and the beta-value clustering. Enrichment is shown for the gold standard clinical data that defines CIMP subgroups in these three cancer sites and compared for both the DM-value clustering and the beta-value clustering.

Supplementary Table 11: Comparison of the known CIMP subtypes for COAD, LAML and GBM between the DM-value clustering and RPMM clustering. Enrichment is shown for the gold standard clinical data that defines CIMP subgroups in these three cancer sites and compared for both the DM-value clustering and the beta-value clustering.

Supplementary Table 12: Comparison between DM-value clustering and gene expression clustering based on the intra-cluster and inter-cluster consensus, and using the Jaccard coefficient for each cancer site.

Supplementary Table 13: Pancancer cluster composition according to the twelve cancer sites.

Supplementary Table 14: Exclusive enrichment of gene sets for each of the ten pancancer clusters.

Supplementary Table 1

	MethylMix			IMA							
Cancer site code	Nr Hyper	Nr Hypo	Nr Dual	Nr Hyper	Nr Hypo	Nr Hyper Overlap	Nr Hypo Overlap	Overlap with methylmix		Overlap with IMA	
BLCA	443	74	23	290	1739	34	10	8%	14%	12%	1%
BRCA	798	203	132	2223	1395	369	71	46%	35%	17%	5%
COAD	526	102	72	1768	1357	233	39	44%	38%	13%	3%
GBM	246	140	22	958	1512	61	51	25%	36%	6%	3%
HNSC	728	101	42	1278	1288	300	25	41%	25%	23%	2%
KIRC	319	251	32	928	927	102	142	32%	57%	11%	15%
LAML	470	77	164	1887	330	119	17	25%	22%	6%	5%
LUAD	576	182	39	1221	1057	198	58	34%	32%	16%	5%
LUSC	605	133	38	881	1607	196	53	32%	40%	22%	3%
OV	234	229	66	479	2015	51	109	22%	48%	11%	5%
READ	321	75	37	1431	1617	134	35	42%	47%	9%	2%
UCEC	618	238	77	1172	1862	234	113	38%	47%	20%	6%

	COHCAP							
Cancer site code	Nr Hyper	Nr Hypo	Nr Hyper Overlap	Nr Hypo Overlap	Overlap with methylmix		Overlap with COHCAP	
BLCA	306	1939	45	18	10%	24%	15%	1%
BRCA	2199	1430	391	78	49%	38%	18%	5%
COAD	1687	1278	239	39	45%	38%	14%	3%
GBM	897	718	75	20	30%	14%	8%	3%
HNSC	1239	1261	302	25	41%	25%	24%	2%
KIRC	890	891	102	142	32%	57%	11%	16%
LAML	1881	343	123	17	26%	22%	7%	5%
LUAD	1179	1051	200	58	35%	32%	17%	6%
LUSC	866	1589	198	53	33%	40%	23%	3%
OV	701	2362	66	130	28%	57%	9%	6%
READ	1433	1657	148	39	46%	52%	10%	2%
UCEC	1235	1813	266	114	43%	48%	22%	6%

	minfi					
Cancer site code	Nr Hyper	Nr Hypo	Nr Hyper Overlap	Nr Hypo Overlap	Overlap with methylmix	Overlap with minfi
BLCA	1764	0	188	0	42%	11%
BRCA	3559	0	456	0	57%	13%
COAD	2933	0	282	0	54%	10%
GBM	2408	0	87	0	35%	4%
HNSC	2473	0	345	0	47%	14%
KIRC	1765	0	164	0	51%	9%
LAML	2233	0	131	0	28%	6%
LUAD	2220	0	290	0	50%	13%
LUSC	2440	0	317	0	52%	13%
OV	2038	0	91	0	39%	4%
READ	2779	0	199	0	62%	7%
UCEC	3007	0	347	0	56%	12%

Supplementary Table 2

Supplementary Table 2A: Absolute number and proportion of transcriptionally predictive genes of all genes uniquely identified by IMA, COHCAP and minfi

	IMA				COHCAP				Minfi	
**Cancer site**	**Number of Transcriptionally predictive Hyper**	**% Transcriptionally predictive Hyper**	**Number of Transcriptionally predictive Hypo**	**% Transcriptionally predictive Hypo**	**Number of Transcriptionally predictive Hyper**	**% Transcriptionally predictive Hyper**	**Number of Transcriptionally predictive Hypo**	**% Transcriptionally predictive Hypo**	**Number of Transcriptionally predictive Hyper**	**% Transcriptionally predictive Hyper**
BLCA	6	2%	196	12%	33	13%	221	12%	37	2%
BRCA	56	3%	110	9%	64	4%	116	9%	174	6%
COAD	25	2%	57	5%	25	2%	57	5%	72	3%
GBM	20	2%	34	2%	50	6%	23	3%	81	3%
HNSC	9	1%	62	5%	10	1%	62	5%	47	2%
KIRC	12	2%	74	10%	12	2%	74	10%	160	10%
LAML	37	2%	19	6%	54	3%	24	7%	85	4%
LUAD	8	1%	100	10%	8	1%	101	10%	76	4%
LUSC	18	3%	141	9%	18	3%	141	9%	90	4%
OV	14	3%	91	5%	33	5%	107	5%	129	7%
READ	21	2%	108	7%	33	3%	116	7%	91	4%
UCEC	17	2%	98	6%	26	3%	98	6%	153	6%
Average	*20*	*2%*	*91*	*7%*	*31*	*4%*	*95*	*7%*	*100*	*5%*

Supplementary Table 2B: For all transcriptionally predictive genes: prevalence of hyper and hypomethylated genes identified by MethylMix, IMA, COHCAP and minfi

	Hyper				Hypo			
**Cancer site**	**MethylMix <15% prevalence**	**IMA <15% prevalence**	**COHCAP <15% prevalence**	**minfi <15% prevalence**	**MethylMix <15% prevalence**	**IMA <15% prevalence**	**COHCAP <15% prevalence**	**minfi <15% prevalence**
BLCA	73	0	1	1	1	0	0	n/a
BRCA	153	11	11	15	28	1	1	n/a
COAD	92	2	3	4	12	0	0	n/a
GBM	33	2	2	2	2	0	1	n/a
HNSC	133	5	5	6	12	1	1	n/a
KIRC	57	2	2	3	26	4	4	n/a
LAML	181	6	6	8	24	0	0	n/a
LUAD	101	3	3	7	11	0	0	n/a
LUSC	137	2	2	2	15	0	0	n/a
OV	25	0	0	0	18	1	1	n/a
READ	48	1	2	1	3	3	3	n/a
UCEC	98	5	5	8	25	2	2	n/a
Average	*94*	*3*	*4*	*5*	*15*	*1*	*1*	*n/a*

Supplementary Table 3

Supplementary Table 3A Number of cancer stage driver genes identified per cancer site

Tissue code	Nr Samples	Nr cancer stage drivers	Nr cancer stage drivers-oncogenes	Nr cancer stage drivers-suppresor genes
BLCA	92	0	0	0
**BRCA**	**775**	**1128**	**249**	**879**
COAD	190	49	8	41
GBM		GBM is only advanced stage		
HNSC	257	43	25	18
**KIRC**	**470**	**6320**	**3969**	**2351**
LAML		No stage information for LAML		
**LUAD**	**344**	**2353**	**509**	**1844**
LUSC	219	1	0	1
OV		Ovary is only advanced stage		
READ	71	0	0	0
**UCEC**	**331**	**4093**	**2349**	**1744**

Supplementary Table 3B Overlap of cancer stage driver genes with MethylMix and IMA

Tissue code	MethylMix overlap with cancer stage drivers	**MethylMix overlap with cancer stage drivers (%)**	IMA overlap with cancer stage drivers	**IMA overlap with cancer stage drivers (%)**
**BRCA**	108	**8.5%**	194	**5.6%**
**KIRC**	305	**48.1%**	562	**31.6%**
**LUAD**	130	**15.6%**	241	**10.9%**
**UCEC**	152	**15.1%**	335	**11.3%**

Text

Tissue code	COHCAP overlap with cancer stage drivers	**COHCAP overlap with cancer stage drivers (%)**
**BRCA**	202	**5.6%**
**KIRC**	562	**31.6%**
**LUAD**	234	**10.5%**
**UCEC**	342	**11.2%**

Tissue code	minfi overlap with cancer stage drivers	**minfi overlap with cancer stage drivers (%)**
**BRCA**	256	**7.2%**
**KIRC**	311	**17.6%**
**LUAD**	320	**14.4%**
**UCEC**	372	**12.4%**

Supplementary Table 4

Gene	BLCA	BRCA	COAD	GBM	HNSC	KIRC	LAML	LUAD	LUSC	OV	READ	UCEC	SumNonZero
TMEM25	0.2439	0.060703	1	0	0.39032	0.32	0	0.20698	0.1676	0.19007	1	0.094	10
ZNF135	0	0.34185	0.96867	0.33582	0.94194	0.436	0	0.54651	1	0.066781	0.97531	0.532	10
ZNF354C	0.43902	0.46645	0.86988	0	0.62581	1	0.65464	0.48837	0.26816	0	0.7284	1	10
ZNF415	0	0.13099	0.8	0.13184	1	0.328	0.27835	0.44651	0.47486	0	0.70988	0.138	10
ZNF542	0.39024	0.20128	1	0	0.84194	0.33	0.10825	0.39535	0.51955	0	1	0.218	10
ZNF671	1	0.38978	0.91807	0	0.79355	0.262	0	0.36512	0.56145	0.29795	0.9321	0.478	10
ZSCAN18	0.38211	0.26837	1	0.1592	0.60323	0.148	0	0.29302	0.34637	0	0.64198	0.042	10
ALDOC	0.31707	0.58147	0	0	0.30323	0.206	0.41753	0.27907	0.10056	0.38014	0	0.508	9
CD40	0	0.82428	0.78313	0	0.2129	0.214	0.48454	0.34651	0.29888	0	1	0.4	9
HIST1H3G	0.30081	0.5623	0.2	0	0.10645	0	0.34021	0.66977	0.2905	0	0.1358	0.444	9
HOXB4	0.10569	1	0.19036	0	0.69355	0	0.26804	0.8	0.71229	0	0.24691	0.106	9
PKIA	0.23577	0.6262	0.66747	0	0.51613	0	0.18557	0.52558	0.35475	0.42466	1	0	9
SMO	0.47154	0.4377	0.39518	0	0.33226	0	0	0.51395	0.27933	0.29281	0.46914	0.086	9
ZNF471	0	1	0.86506	0	1	1	0.17526	1	1	0	0.87037	0.352	9
ZNF501	0.17886	1	0.17349	0	0.18065	0.358	0.07732	0.15581	0.083799	0	0	0.65	9
ZNF502	0	1	0.28916	0	0.2871	0.214	0	0.43721	0.24022	0.18836	0.08642	0.638	9
ZNF655	0.12195	0.15335	0.55904	0	0.074194	0	0	0.12791	0.2067	0.15753	1	0.14	9
AKR1B1	0	0.63259	0.7494	0	0.032258	0	0.12371	0.40233	0.10615	0	0.74074	0.264	8
CAPS	1	0	0.50843	0	0.65806	0.828	0	0.2907	0.37989	1	0.45062	0	8
CDO1	1	0.80831	1	0	1	0	0	1	1	1	0	1	8
FUZ	0.14634	0.2492	1	0	0.34516	0	0	0.15349	0.10615	0	0.58642	0.14	8
HIST1H2BH	0.30081	0.28115	0.2988	0	0.058065	0	0.097938	0.92326	0.2095	0	0	0.312	8
HOXA9	0.56911	0.75719	0.25542	0	0.92258	0.22	0.19588	0	0	0	0.48765	0.716	8
KLHL3	0	0.47604	0.51807	0	1	0.584	0	0.62093	0.32961	0	1	0.156	8
LTBP3	0	0.16613	0.53253	0	0.5	1	0.45876	0	0	0.2226	1	0.39	8
MGMT	0.15447	0.054313	0.35422	0	0.30968	0	0.1701	0.11628	0.13687	0	0.40741	0	8
MPV17L	0.22764	0.13738	0.38795	0	0.46452	0	0	0.44186	0.18436	0	0.40741	0.362	8
MT1E	0	0.377	0	0	0.48065	0.546	0	0.32093	0.50838	0.13699	0.098765	0.538	8
NUPR1	0	0.38019	1	0	1	0	0	1	1	0.51541	1	1	8
PTPN20B	0	1	1	0	1	0	0.30412	1	1	0	1	1	8
RBP1	0.26016	0.11182	0.65783	0.11194	0.070968	0	0	0	0.053073	0.39041	1	0	8
SPAG16	0	0.42173	0.39518	0	0.23871	0	0.14433	0.07907	0.027933	0	0.098765	0.254	8
TSPYL5	0	1	1	0.9801	1	0.478	1	0	0	0	1	0.982	8
WASF3	0.089431	0.23003	1	0	0.46129	0.316	0	0.34651	0	0	0.52469	0.136	8
XKR6	0	1	0.6241	0	0.2	0.306	0.81443	0.57209	0.2486	0	0	0.496	8
ZFP28	0	0.22684	0.68193	0	0.5	1	0	0.37442	0.19274	0	0.6358	0.096	8
ZNF134	0	1	0.93012	0	0.72581	0.35	0	0.45116	0.3324	0	0.46914	0.228	8
ZNF256	0.14634	0.063898	0.72771	0	0.45161	0	0	0.33953	0.20112	0	0.5679	0.094	8
ZNF300	0	1	0.82169	0	0	1	0	1	0.18156	0.21747	0.71605	0.488	8
ZNF350	0.097561	0	0.44337	0	0.46774	0.126	0	0.12558	0.22626	0	0.17901	0.066	8
ZNF570	0.20325	0.054313	0.51325	0	0.50968	0.146	0	0.22791	0.18994	0	0.41975	0	8

ZNF577	0	1	0.50843	0.45771	0	0.602	0.28866	0.63721	0	0.25685	0	0.316	8
ZNF625	0	0.063898	1	0	0.69355	0.106	0.16495	0.26512	0.42737	0	0.88889	0	8
ALKBH3	0.11382	0.20447	0.12771	0	0	0	0	0.13488	0.072626	0	0.10494	0.444	7
C1orf51	0	0.19489	0.72048	0	1	0.232	0	0	0.29609	0	0.78395	0.168	7
C3orf14	0	0.12141	0.66506	0	0.24516	0.156	0	1	0.12291	0	0	0.494	7
C7orf13	0.35772	0.13099	0.033735	0.88806	0.29355	0	0	0	0.10335	0	0	0.308	7
CMBL	0	0.092652	0.2241	0	0	0	0	0.42558	0.036313	0.45548	0.21605	0.24	7
CRISPLD1	0.39837	1	0	0	0.50645	0	0	0.1907	0.13408	0.2089	0	0.094	7
CWH43	0.17886	0.17572	0.33976	0	1	0.126	0	0	1	0	0	0.148	7
DMC1	0	1	0	0	0.29677	0	0.31443	0.35814	0.4581	0.20719	0	0.494	7
DNALI1	0	0.42173	0	0	1	1	0	0.22791	1	0.47603	0	0.422	7
GPX7	0	0.36102	0.53253	0	0.46452	0.314	0	0.36977	0.31285	0	0	0.084	7
HPDL	0.39024	0.23642	0.11566	0.31343	0.2	0	0	0	0.15642	0	0	0.288	7
HSPA2	1	0	0	1	0.3	0	0	1	1	0	1	1	7
IFFO1	1	1	1	0	0	0	0	1	1	0	1	1	7
L3MBTL4	0	1	0.61205	0	0.33548	0	0.26289	0.18372	0	0	0.5	0.156	7
MAP9	0.38211	0.21725	0	0	0.59677	0.324	0	0.13488	0.20112	0	0	0.046	7
MFSD7	0	0.42812	0.83614	0	0	0.604	0.39175	0.28605	0.60335	0	0.82099	0	7
MRPS21	0.23577	0.34505	0	0	0.19355	0	0	0.15581	0.19553	0.57877	0	0.404	7
MYEF2	0.35772	0	0.3494	0	0.75806	0	0.24742	0.062791	0.19274	0	0.24074	0	7
NPTX2	0	1	0.80482	0	1	0	0	0.81628	1	0	0.77778	1	7
NRSN2	0.34146	0.044728	0.28916	0	0.1129	0	0	0.053488	0	0	0.32716	0.09	7
NSUN7	0	0.095847	0.15422	0.28358	0.26129	0.144	0	0	0.072626	0	0	0.052	7
PCDHB2	0	0.61022	0	0	1	1	0	0	1	0.47432	0.64815	1	7
PCDHB5	0	0.58786	0	0.26866	0.65806	1	0	1	1	0.55822	0	0	7
RARRES3	0.4065	0.11182	0	0	0.26129	0	0	0.15116	0.28212	0.094178	0	0.252	7
RPL39L	1	0.33546	0.63133	1	0.46129	0.238	0	0	0	0	1	0	7
SLC16A5	0.33333	0.36741	0.096386	0	0.11935	0	0	0.20465	0.29609	0	0	0.4	7
TMEM98	0	0.21406	0.096386	0	0.22581	0.076	0	0.13721	0.075419	0	0	0.108	7
TRMT12	0.18699	0	0.33494	0	0.22258	0	0.07732	0	0.064246	0	0.3642	0.186	7
UBXN10	0	0.095847	0.27952	0.83333	0.6	0.168	0	0.29535	0	0	0	0.08	7
VAMP5	0.70732	1	0	0	0.69032	0	0.59794	0.77209	0.74581	0	0	1	7
ZFP3	0	0	0.32289	0	0.3129	0	0.25258	0.28605	0.29888	0	0.08642	0.594	7
ZFP82	0	0	0.85301	0.25622	0.84194	0	0	0.3093	0.43855	0	0.82099	0.28	7
ZNF132	0.34959	1	0	0	1	0	0	0.53488	1	0.22774	0	1	7
ZNF175	0.11382	0.14377	0	0	0.12581	0	0	0.21163	0.18994	0	0.16049	0.194	7
ZNF214	0.1626	0.28435	0	0	0.58387	0	0.5567	0.28372	0.18436	0	0	0.41	7
ZNF329	0.25203	0	0.30602	0	0.44839	0.112	0	0.5	0.35196	0	0.22222	0	7
ZNF597	1	1	0	0	0	0	1	1	1	0	0.93827	1	7
ZNF660	0.55285	0	0.83373	0	0.82581	0	0	0.49302	0.31564	0	0.7963	0.314	7
ZNF667	0	1	0	0	0.95484	1	0.41237	0.65116	0.63687	0	0	0.44	7
ZNF702P	0	0.12141	0.3253	0	0.81935	0.09	0.072165	0.25814	0.45251	0	0	0	7

ZSCAN12	1	0	0	0	0.70323	0.466	0	1	0.45251	0	1	1	7
ABO	0.21138	0.52396	0	0	0.46774	0	0	0.15116	0.28212	0	0	1	6
ACAA2	0	0.36102	0.06988	0.28358	0	0	0	0	0.5419	0.18836	0	0.41	6
ACOX2	0	1	0.20482	0	0	1	0.69072	0	0	1	0	1	6
ACSS3	0	0.25559	0	0	0	0.08	0	0.34651	0.48603	0.14041	0	0.386	6
ADHFE1	0.58537	0.22045	0	0	0.43548	0	0	0.31395	0.35754	0	0	0.574	6
AMT	0	0.89776	1	0	0	1	0.43814	0	0	0.86473	0	1	6
BASP1	0.18699	0	0	0.4801	0.25806	0	0.10825	0.33953	0	0	0	0.116	6
BNIP3	0.30894	0	0.58554	0	0.18065	0.27	0	0.067442	0	0	0.41358	0	6
C20orf151	1	1	0	0	0	0.942	0	1	1	0	0	1	6
C2orf43	0.12195	0.13099	0	0	0.14839	0	0	0	0.1648	0.23116	0	0.55	6
CARD11	0	0	0.66506	0	0.54516	0	0	0.27209	0.29609	0	0.67284	1	6
CBLC	1	1	0	0	0	0	0	1	1	1	0	1	6
CBR1	0.10569	0.48882	0	0	0.077419	0	0.37113	0	0.097765	0	0	0.106	6
CCDC106	0	0	1	0	1	0.16	0	1	0	0	1	0.078	6
CHFR	0	0	1	0	0.31935	0	0.056701	0	0.25698	0	1	0.134	6
CHST10	0.33333	0	0	0	0.3129	0.134	0	0.13953	0.092179	0	0.75309	0	6

Supplementary Table 5

Gene	BLCA	BRCA	COAD	GBM	HNSC	KIRC	LAML	LUAD	LUSC	OV	READ	UCEC	SumNonZero
MAGEA4	0.42276	0.18211	0.36627	0	0.54839	0	0	0.29302	0.59218	0.60274	0.40123	0.35	9
BATF	0.84553	0.90096	0.50843	0.24378	0	0.334	0	0.56512	0	0.42979	0	1	8
DAPP1	0	0.77955	0.45542	0	0	0.268	0	0.69767	1	1	0.41358	0.372	8
FOXRED2	0.39837	0.41214	0.45542	0	0.37419	0	0	0.3	0.52514	0.3476	0	0.466	8
ACY1	0.36585	0	0.6241	0	0.19032	0	0	0.23023	0.31844	0.46233	0.60494	0	7
BST2	0	0	0.31807	1	0.75806	0.92	0	0.47442	0	0	0.30247	0.534	7
CALML4	0	0.18211	0	0	0.19677	0.584	0	0.43953	0.43296	0.26884	0	0.402	7
MAGEA10	0.64228	0.26198	0	0	0.6871	0	0	0.32093	0.38827	0	0.42593	0.464	7
C2	0	0	1	0	0	0.384	0.25773	0	0	0.56164	1	0.186	6
CALML5	0.17073	0.32268	0	0	0.35161	0	0	0.15116	0.32123	0.32021	0	0	6
CES3	0	0.28435	0	0	0.1871	0.26	0	0.36279	0.28212	0	0	1	6
GPR171	0	0.12141	0	0	0.33871	0.19	0	0.2186	0.34358	0.11473	0	0	6
HAL	0.25203	0	0	0	0	0	0	0.49767	0.3324	0.44521	0.18519	0.444	6
IL20RB	0.36585	0.057508	0.21446	0	0	0.33	0	0.3	0	0	0	0.114	6
NAIF1	0	0.57188	0	0	0.4871	0	0.056701	0.35814	0.53911	0.29452	0	0	6
OAS2	0	0	1	0	0	0.316	0	0.77442	0.98324	0.6387	0	0.436	6
PADI3	0	0.19808	0.21205	0	0.29032	0	0	0	0	0.5976	0.41975	0.434	6
PNLDC1	0.33333	0.1246	0	0	0.39355	0	0	0.11628	0.34637	0.25685	0	0	6
C16orf54	0	0.23003	0.048193	0	0	0.202	0	0	0	0	0.061728	0.216	5
C6orf15	0	0.17572	0.3012	0	0	0	0	0	0	0.21404	0.32716	0.124	5
CD3D	0	0.13099	0.096386	0	0	0.37	0	0	0	0.21575	0	0.162	5
CEL	0.6748	0.23003	0.5253	0	0	0	0	0.2093	0	0	0	0.284	5
CFB	0	0	0.61446	0	0	0.406	0	0	0	0.79966	1	0.56	5
CNTD1	0	0	0.11084	0	0.18387	0	0	0.2	0.29888	0.375	0	0	5
ELF5	0	0.69329	0.35422	0	0.25484	0	0	0	0	0.21233	0.43827	0	5
EPHX4	0	0.34185	0	0	0	0	0	0.67442	0.47207	0.59589	0.67284	0	5
F2	0.21138	0	0.6747	0	0.2	0	0	0.28372	0.15922	0	0	0	5
FKBP10	0	0	0.22892	0	0	0.31	0	0.27209	0.49721	0	0.33333	0	5
GBP4	0	0.5016	0	0	0.72581	1	0	0.52558	0	0	0.69753	0	5
GPX2	0.69919	0	0.58072	0	0	0	0	0.55814	0.80726	0	0	0.296	5
IL2RB	0.21951	0.15016	0	0	0	0.2	0	0.43953	0	0	0	0.286	5
LAT	0.22764	0	0.1494	0	0	1	0	0	0	0	0.2284	0.116	5
MAGEC2	0	0.28754	0	0	0.10968	0	0	0	0	0.71918	0.10494	0.156	5
MSLN	0	0.2524	0.85301	0	0.32258	0	0	0.58605	0.12849	0	0	0	5
PAGE2	0.4878	0	0	0	0.23548	0	0	0.47209	0.31285	0	0	0.31	5
PER3	0.20325	0	0	0	0	0	0	0.52791	0.3743	0.42295	0	0.198	5
S100P	0	0.71885	0	0	0.22903	0	0	0	0.39944	0.54623	0	0.206	5
SYCP2	0.19512	1	0	0	0.30323	0	0	0.15814	0.2067	0	0	0	5
TBC1D10C	0	0.28115	0	0.41542	0.12903	0	0	0	0	0.25856	0	0.11	5
TDRD1	0	0	0	0	0.067742	0	0	0.15814	0.10615	0.25856	0.08642	0	5
ZNF556	0	0	0.28916	0	0	0	0.072165	0.15814	0.70112	0	0.21605	0	5

ZNF572	0.45528	0.377	0	0	0.7129	0	0	0.54651	0.82961	0	0	0	5
ABCC2	0	0	0.58072	0	0	0.772	0	0.52791	0.35196	0	0	0	4
ACSL5	0.19512	0	0.39036	0	0	0	0	0.59767	0	0	0.55556	0	4
AGMAT	0	0	0	0	0.74516	0	0.2732	0	0	0.80137	0	0.612	4
BIN2	0	0.1853	0	0.37562	0	0.29	0	0	0	0	0	0.248	4
BRDT	0.21138	0	0	0	1	0	0	0.73256	0.22905	0	0	0	4
C11orf16	0	0	0	0	0.31613	0	0	0.49302	0.20391	0.36986	0	0	4
C11orf9	0	0	0.34217	0	0	0	0	0.26977	0	0.38356	0.24691	0	4
C1orf64	0	0.70927	0	0	0	0	0	0.33256	0	0.35788	0	0.466	4
CCND1	0	0.22045	0	0.10945	0	0	0.30928	0	0	0	0	0.312	4
CCR7	0	0.29393	0	0	0	0.484	0	0	0	0.15411	0	0.168	4
CP	0.26016	0.3099	0	0	0	0	0	0.68605	0	0.88356	0	0	4
CSTA	0	0	0	0	0	0.06	0	0	1	0.58733	0	0.364	4
CTSE	0	0	0	0	0.24516	0.588	0	0	0	0.21575	0	0.286	4
DENND2D	0	0.8722	0	0.54975	0	0.968	0	0.50233	0	0	0	0	4
DOK2	0	0.36102	0	0	0	0.538	0	0	0	0.37671	0	0.144	4
EPS8L3	0	0	0	0	0	0.81	0	0.36977	0.15642	0	0	0.36	4
FUT6	0	0.3131	0.7253	0	0	0	0	0.37674	0	0	0	0.456	4
FXYD4	0	0.29073	0	0	0	0	0	0.85116	0	0.46062	0	0.602	4
GPT	0.37398	0	0	0	0	0	0.015464	0.34186	0	0	0	0.452	4
IFITM1	0	0	0	0	0.86774	0.64	0	0	0	0	0.7716	0.756	4
INSL6	0	0.36422	0	0	0.13548	0	0	0.17209	0	0.25514	0	0	4
KAZALD1	0	0	0	0.43781	0.26129	0	0	0	0	0.80651	0	0.818	4
KCNJ11	0	0	0	0	0	0.268	0	0.32326	0.49162	0	0	0.52	4
KRT80	0	0.28754	0	0	0	0.388	0	0.39302	0	0.31164	0	0	4
LGALS4	0	0	0	0	0.18387	0.834	0	0.30698	0.12291	0	0	0	4
LPIN2	0	0.26837	0	0	0	0	0	0.53721	0	0	0.23457	0.206	4
LY75	0.69919	0	0	0.22139	0	0	0	0	0	1	0	0.38	4
MIA	0	0	0	0	0	0	0	0.26744	0.38268	0.81336	0	0.3	4
MKRN3	0	0	0.46024	0	0	0	0	0.3907	0	0	0.2284	0.54	4
NCF2	0	0	0.53012	0	0	0.34	0	0	0	0.37842	0	0.072	4
NCF4	0	0.15974	0	0.16418	0	0	0	0	0	0.083904	0	0.206	4
NLRP2	0	0	0	0	0.31935	0	0	0.46977	0.63687	0.62158	0	0	4
PPP1R14D	0	0	0	0	0	0.598	0	0.49767	0.14246	0.28425	0	0	4
PTPN22	0	0	0	0.37313	0	0.386	0	0	0	0.38527	0	0.274	4
PTPN7	0	0.095847	0	0	0	0.298	0	0	0	0.2089	0	0.274	4
RAPSN	0	0.17891	0	0	0	0	0	0	0.54749	0.44521	0	0.208	4
RDH5	0	0	0	0.47015	0	0.826	0	0.55349	0.39106	0	0	0	4
RLBP1	0	0	0	0.65672	0.56452	0	0	0.45814	0.59777	0	0	0	4
S100A5	0	0	0	0	0	0	0	0.1186	0.041899	0.41438	0	0.078	4
SERPINF1	0	0	0	0	0.15806	0.072	0	0.18605	0.30168	0	0	0	4
SLC28A2	0	0	0.2747	0	0	0	0	0.36744	0	0.15925	0	0.258	4

SNX20	0	0.23003	0.30843	0	0	0	0	0	0	0.20205	0	0.148	4
TFF1	0	0.7476	0.37349	0	0	0	0	0	0	0	0.2037	0.334	4
TMEM45B	0.46341	0	0.4241	0	0	0	0	0.3	0.12849	0	0	0	4
TRAF1	0.25203	0	0.21687	0	0	0	0	0	0	0.48801	0	0.204	4
UGT1A10	0	0	0	0	0.3871	0.154	0	0.24651	0.35475	0	0	0	4
AGT	0	0	0.30843	0	0	0.792	0	0	0	0	0.34568	0	3
ALOX12	0	0	0	0	0.23871	0	0	0	0.32682	0.27911	0	0	3
ALPPL2	0	0	0.17349	0	0	0	0	0.35814	0	0.52226	0	0	3
ARL14	0	0.26837	0	0	0	0	0	0.53953	0.67598	0	0	0	3
BEST3	0	0.68051	0	0.63184	0	0	0	0	0	0	0	0.426	3
C12orf40	0	0.10863	0	0	0.17419	0	0	0	0.20112	0	0	0	3
CAPN9	0	0	0.22169	0	0	0	0	0	0	0.32534	0.32099	0	3
CARD14	0	0	0.90602	0	0	0.472	0	0.41163	0	0	0	0	3
CASP8	0	0	0	0	0	0.962	0	0.84419	0	0	0	1	3
CCDC109B	0	0.7508	0	0.60199	0	0.704	0	0	0	0	0	0	3
CCL5	0	0.20128	0	0	0	0	0	0	0	0.082192	0	0.166	3
CD5	0	0	0	0	0	0.346	0	0	0	0.40925	0	0.236	3

Supplementary Table 6

Gene	BLCA	BRCA	COAD	GBM	HNSC	KIRC	LAML	LUAD	LUSC	OV	READ	UCEC	SumNonZero
TMEM25	4.9629	2.3214		0	2.5619	2.554	0	2.0315	1.9755	1.712		2.1877	10
ZNF135	0	3.08	4.6306	4.8473	3.8038	2.0882	0	3.4496		2.5269	10.1204	6.2999	10
ZNF354C	8.8522	2.034	5.7491	0	4.0254		5.4091	1.9059	2.7876	0	4.188		10
ZNF415	0	2.3543	5.2411	2.5323		2.0386	3.791	2.6187	3.0018	0	3.9336	1.896	10
ZNF542	5.1227	3.8443		0	3.6423	2.131	2.4358	2.745	3.2964	0		2.3793	10
ZNF671		3.5232	1.4972	0	2.1518	2.0315	0	2.1517	1.93	2.3283	1.8826	4.3041	10
ZSCAN18	3.9264	2.925			2.9995	2.4256	0	2.7589	2.726	0	3.1546	2.5483	10
ALDOC	2.2257	3.666	0	0	3.4461	3.9404	2.1322	1.7433	2.9785	3.583	0	2.7927	9
CD40	0	1.642	1.988	0	1.8215	1.5798	2.1599	1.9194	2.1344	0		1.8948	9
HIST1H3G	9.0048	19.0385	11.6388	0	5.5245	0	14.3751	11.1234	25.2158	0	10.1933	9.9414	9
HOXB4	2.4565		3.2552	0	9.8028	0	31.8709	2.7194	2.4948	0	2.9554	6.7129	9
SMO	3.0344	2.6971	2.0091	0	2.4891	0	0	2.246	2.7464	1.8628	2.0633	2.7471	9
ZNF471	0		5.9848	0			2.9968			0	7.5064	2.771	9
ZNF501	2.1432		2.2014	0	3.0129	1.4343	4.0526	2.8573	4.0161	0	0	4.2386	9
ZNF502	0		2.9616	0	2.6217	1.4899	0	2.5908	5.1255	3.3838	2.2123	6.0021	9
ZNF655	4.8696	2.8059	2.3455	0	1.5122	0	0	1.4747	1.5837	2.2547		2.4927	9
AKR1B1	0	1.5971	1.7356	0	3.3458	0	5.0755	1.9818	2.4404	0	2.4531	3.1501	8
CAPS		0	3.2399	0	2.4731	2.5122	0	4.4824	2.6832		2.5607	0	8
CDO1		3.3158		0		0	0				0		8
FUZ	2.1737	1.548		0	2.8626	0	0	1.8934	2.7837	0	2.6815	3.0091	8
HIST1H2BH	12.7464	9.443	4.1287	0	5.5142	0	50.0786	5.3012	8.8422	0	0	6.5244	8
HOXA9	17.0205	2.5771	3.0735	0	17.2267	2.427	49.4786	0	0	0	4.6334	9.3592	8
KLHL3	0	1.4373	3.1775	0		1.6214	0	1.5259	1.4925	0		3.7792	8
LTBP3	0	1.8914	1.5275	0	2.0994		3.5892	0	0	1.4539		2.0718	8
MGMT	4.8826	3.1205	3.5908	0	3.3857	0	8.258	1.7839	3.1644	0	3.5994	0	8
MPV17L	3.7945	7.4395	5.215	0	6.6867	0	0	3.4483	5.3426	0	6.0448	7.9446	8
MT1E	0	3.4485	0	0	3.2224	2.5976	0	2.3995	3.3405	4.1687	4.3195	4.5042	8
NUPR1	0	1.5026		0		0	0			2.2043			8
PKIA	2.3969	2.2391	5.2298	0	2.313	0	0	2.2372	2.3483	2.14		0	8
PTPN20B	0			0		0	57.9802			0			8
RBP1	2.1801	3.2273	3.4707	17.0159	3.419	0	0	0	5.11	1.7748		0	8
SPAG16	0	3.0094	5.1616	0	3.2156	0	14.4883	2.6784	9.8953	0	2.9413	10.0731	8
TSPYL5	0			9.9698		1.7302		0	0	0		1.7332	8
WASF3	3.5186	3.4534		0	1.9944	1.5247	0	1.9996	0	0	4.2528	3.0359	8
XKR6	0		15.6493	0	4.4505	2.3633	20.6124	3.326	4.2689	0	0	7.6174	8
ZFP28	0	1.6503	4.2715	0	2.6362		0	1.8288	2.9398	0	3.5112	4.71	8
ZNF134	0		2.7276	0	1.5788	1.3756	0	1.332	1.4581	0	3.2632	1.4327	8
ZNF256	3.886	2.2112	6.1406	0	2.9848	0	0	2.8029	3.3827	0	4.7559	3.2415	8
ZNF300	0		5.8085	0	0		0		4.6007	3.668	4.6397	11.0533	8
ZNF350	2.1049	0	3.2113	0	2.7641	1.6719	0	1.6127	2.1557	0	1.704	1.6267	8
ZNF570	4.3263	1.5363	5.8058	0	3.3898	1.7341	0	1.9343	3.4958	0	2.4234	0	8

ZNF577	0		2.9024	1.8252	0	1.9835	1.5593	1.7022	0	1.5655	0	1.6656	8
ZNF625	0	2.5242		0	3.7309	1.768	2.6711	3.1594	2.8082	0	11.5292	0	8
ALKBH3	1.82	2.7251	2.118	0	0	0	0	2.2687	2.9717	0	3.8863	4.9867	7
C1orf51	0	2.6297	2.8115	0		1.8866	0	0	3.2193	0	2.6719	2.2292	7
C3orf14	0	3.2888	5.0399	0	3.0688	2.4196	0		5.4927	0	0	2.9028	7
C7orf13	4.1191	3.6386	2.9218	4.6517	3.7193	0	0	0	2.5444	0	0	5.587	7
CMBL	0	8.0516	2.1478	0	0	0	0	3.4994	4.1604	3.1573	3.8687	2.8975	7
CRISPLD1	3.1782		0	0	3.3963	0	0	4.3067	2.805	3.5034	0	7.1833	7
CWH43	103.7874	84.0701	30.767	0		33.5786	0	0		0	0	13.5473	7
DMC1	0		0	0	2.429	0	3.0052	2.4719	3.4096	5.0873	0	3.2679	7
DNALI1	0	10.5954	0	0			0	5.1224		4.8782	0	4.2038	7
GPX7	0	1.8058	2.1679	0	1.8593	1.7313	0	2.1932	2.8696	0	0	2.4863	7
HPDL	9.9452	3.7085	3.5893	2.5644	6.4528	0	0	0	4.4748	0	0	4.5193	7
HSPA2		0	0		3.0938	0	0			0			7
IFFO1				0	0	0	0			0			7
L3MBTL4	0		2.3812	0	3.1686	0	8.8576	1.9013	0	0	6.0358	3.3668	7
MAP9	4.5812	3.253	0	0	4.0262	2.4787	0	1.8754	1.9976	0	0	5.6257	7
MFSD7	0	4.0222	2.1309	0	0	2.2388	2.1834	2.2014	1.7742	0	1.699	0	7
MRPS21	2.42	1.7729	0	0	1.5458	0	0	1.3818	1.3231	1.2406	0	2.1498	7
MYEF2	4.6376	0	7.2273	0	3.653	0	10.699	6.0717	3.6829	0	8.2468	0	7
NPTX2	0		5.521	0		0	0	3.2108		0	12.7081		7
NRSN2	2.6982	1.788	2.0456	0	1.8939	0	0	2.4846	0	0	1.6171	1.5447	7
NSUN7	0	7.4734	3.3461	6.9397	6.0018	5.2114	0	0	4.313	0	0	10.6486	7
PCDHB2	0	3.3054	0	0			0	0		3.5111	2.0332		7
PCDHB5	0	2.2158	0	8.0894	4.0946		0			4.2873	0	0	7
RARRES3	3.3052	4.953	0	0	3.2983	0	0	2.8392	1.8676	4.305	0	2.9231	7
RPL39L		2.9489	7.1243		5.3457	1.8523	0	0	0	0		0	7
SLC16A5	3.4256	3.1643	2.4808	0	2.2027	0	0	1.9923	2.0844	0	0	4.279	7
TMEM98	0	2.3928	1.5762	0	1.8577	2.7841	0	2.364	3.4339	0	0	3.3787	7
TRMT12	1.3348	0	2.5553	0	1.9083	0	1.4015	0	1.734	0	2.259	3.1485	7
UBXN10	0	5.5727	2.9411	8.7534	3.0688	2.1574	0	2.5846	0	0	0	3.0549	7
VAMP5	2.2211		0	0	1.3952	0	3.3247	1.6445	1.9622	0	0		7
ZFP3	0	0	2.79	0	3.0497	0	2.4865	2.0099	2.8483	0	1.8358	4.5334	7
ZFP82	0	0	3.3156	2.1374	3.5594	0	0	2.6207	3.9837	0	2.7154	4.2694	7
ZNF132	2.121		0	0		0	0	1.9473		1.9468	0		7
ZNF175	3.5821	2.5014	0	0	2.0408	0	0	1.7456	2.2993	0	3.4849	3.4854	7
ZNF214	17.4639	3.7334	0	0	6.4405	0	14.4736	2.8248	2.4616	0	0	10.0023	7
ZNF329	2.482	0	2.4074	0	2.771	1.7537	0	1.5433	1.751	0	2.1119	0	7
ZNF597			0	0	0	0				0	1.3123		7
ZNF660	3.2005	0	7.3516	0	3.1097	0	0	2.3256	3.7359	0	5.4377	5.3546	7
ZNF667	0		0	0	17.5866		17.5626	5.6431	8.2995	0	0	12.3974	7
ZNF702P	0	2.4731	3.3745	0	4.4316	2.711	7.7618	3.17	2.7959	0	0	0	7

ZSCAN12		0	0	0	2.1622	1.4094	0		1.5866	0			7
ABO	12.1759	3.229	0	0	9.2766	0	0	8.2995	6.1454	0	0		6
ACAA2	0	1.5262	2.4382	2.0172	0	0	0	0	2.4457	1.6916	0	3.0734	6
ACOX2	0		4.5663	0	0		10.0859	0	0		0		6
ACSS3	0	3.527	0	0	0	3.346	0	2.0194	2.0866	3.6942	0	3.6573	6
ADHFE1	3.9769	2.8567	0	0	2.9222	0	0	3.0876	2.1009	0	0	5.5478	6
AMT	0	3.078		0	0		1.2875	0	0	5.1651	0		6
BASP1	4.8365	0	0	1.7319	2.4465	0	8.0411	2.8075	0	0	0	2.8478	6
BNIP3	1.81	0	4.0181	0	2.0849	1.6144	0	2.6301	0	0	2.9392	0	6
C20orf151			0	0	0	112.1344	0			0	0		6
C2orf43	1.8723	2.523	0	0	1.9005	0	0	0	1.6643	1.6905	0	4.7652	6
CARD11	0	0	5.528	0	2.2819	0	0	2.4956	2.5445	0	5.9666		6
CBLC			0	0	0	0	0				0		6
CBR1	3.0295	2.7788	0	0	1.7875	0	2.4402	0	2.8963	0	0	3.5091	6
CCDC106	0	0		0		1.5429	0		0	0		1.4992	6
CHFR	0	0		0	2.5724	0	3.3795	0	2.1457	0		1.8213	6
CHST10	2.5499	0	0	0	3.4671	1.7699	0	3.6609	3.3833	0	2.6009	0	6

Supplementary Table 7

Gene	BLCA	BRCA	COAD	GBM	HNSC	KIRC	LAML	LUAD	LUSC	OV	READ	UCEC	SumNonZero
MAGEA4	339.2904	28.2628	16.1365	0	53.7881	0	0	293.1677	194.8752	58.2436	31.0221	72.6299	9
BATF	8.6382	2.8322	3.4132	3.0101	0	2.5442	0	2.0325	0	2.2871	0		8
DAPP1	0	2.8294	3.9399	0	0	1.9963	0	2.4607			4.4443	2.909	8
FOXRED2	1.6466	1.4717	1.4284	0	2.4758	0	0	1.8947	1.6972	1.461	0	1.4329	8
ACY1	2.5349	0	1.832	0	1.6599	0	0	1.6597	1.5257	1.5605	1.8405	0	7
BST2	0	0	5.3227		5.095	3.31	0	2.3388	0	0	4.5068	7.028	7
CALML4	0	1.5116	0	0	1.4362	1.4174	0	1.801	1.7793	1.5809	0	1.7114	7
MAGEA10	111.8364	13.9385	0	0	11.8939	0	0	33.9489	600.8541	0	2.5734	8.891	7
C2	0	0		0	0	2.0901	20.0038	0	0	2.8805		3.0957	6
CALML5	91.2396	94.581	0	0	8.8473	0	0	24.2645	34.3671	16.6145	0	0	6
CES3	0	11.6694	0	0	3.4693	3.7614	0	2.892	4.8953	0	0		6
GPR171	0	5.168	0	0	2.1218	2.3332	0	3.5354	2.1077	3.3475	0	0	6
HAL	4.2208	0	0	0	0	0	0	14.2004	8.3382	19.8681	4.1314	6.9815	6
IL20RB	13.5905	3.0877	2.5678	0	0	31.9998	0	14.3686	0	0	0	1.9477	6
NAIF1	0	1.3336	0	0	1.3123	0	1.4276	1.3548	1.2371	1.1376	0	0	6
OAS2	0	0		0	0	1.4824	0	1.8292		2.5717	0	3.5823	6
PADI3	0	23.4719	6.0035	0	5.344	0	0	0	0	4.9973	4.8961	5.3786	6
PNLDC1	13.409	39.8992	0	0	38.8756	0	0	11.3196	58.4574	6.5522	0	0	6
C16orf54	0	3.741	2.9809	0	0	2.1097	0	0	0	0	7.5785	3.5741	5
C6orf15	0	16.5124	14.1669	0	0	0	0	0	0	31.2748	10.0249	40.27	5
CD3D	0	4.8019	1.6006	0	0	3.12	0	0	0	4.429	0	4.9116	5
CEL	7.7653	5.8187	26.2127	0	0	0	0	4.2113	0	0	0	3.4829	5
CFB	0	0	1.8368	0	0	2.3713	0	0	0	3.5866		3.8466	5
CNTD1	0	0	2.37	0	2.8929	0	0	1.7222	1.5625	1.7657	0	0	5
ELF5	0	29.7985	332.7208	0	12.1468	0	0	0	0	35.4709	247.9404	0	5
EPHX4	0	5.7735	0	0	0	0	0	3.383	3.0819	2.3007	2.3979	0	5
F2	10.1353	0	12.8001	0	13.1122	0	0	68.4565	11.3931	0	0	0	5
FKBP10	0	0	2.3885	0	0	2.3362	0	3.2861	2.2074	0	3.1016	0	5
GBP4	0	2.1297	0	0	2.5704		0	2.1247	0	0	2.2759	0	5
GPX2	368.7755	0	2.1843	0	0	0	0	101.1866	50.8495	0	0	27.7266	5
IL2RB	3.8483	4.9557	0	0	0	2.4404	0	2.5261	0	0	0	2.7921	5
LAT	2.0912	0	1.9168	0	0		0	0	0	0	1.5514	2.3848	5
MAGEC2	0	95.0366	0	0	1570.1837	0	0	0	0	20.0509	6.2029	440.696	5
MSLN	0	23.3751	4.1611	0	31.1843	0	0	23.6879	6.6195	0	0	0	5
PAGE2	10.6896	0	0	0	13.6748	0	0	10.6141	89.5748	0	0	11.3319	5
PER3	2.6034	0	0	0	0	0	0	2.4344	2.7386	1.7568	0	2.3907	5
S100P	0	18.8587	0	0	2.5991	0	0	0	7.8271	2.5586	0	4.0842	5
SYCP2	3.0637		0	0	8.2886	0	0	8.9836	5.2581	0	0	0	5
TBC1D10C	0	3.5335	0	1.9652	1.8357	0	0	0	0	2.5131	0	3.49	5
TDRD1	0	0	0	0	226.8747	0	0	94.6054	14.2354	2.5512	295.9944	0	5
ZNF556	0	0	14.4978	0	0	0	95.5104	38.9975	182.123	0	134.753	0	5

ABCC2	0	0	7.1562	0	0	4.6148	0	7.0383	3.4819	0	0	0	4
ACSL5	2.1436	0	1.5707	0	0	0	0	1.7611	0	0	1.4974	0	4
BIN2	0	3.3275	0	1.7456	0	1.9978	0	0	0	0	0	3.0252	4
BRDT	5.4434	0	0	0		0	0	11.0146	1496.7892	0	0	0	4
C11orf16	0	0	0	0	34.208	0	0	5.5419	12.7614	10.9643	0	0	4
C11orf9	0	0	3.2685	0	0	0	0	1.9969	0	3.5397	2.1006	0	4
C1orf64	0	250.3954	0	0	0	0	0	19.3649	0	2.5766	0	173.4098	4
CCR7	0	4.1064	0	0	0	2.1107	0	0	0	1.8598	0	2.8817	4
CP	36.7606	18.6177	0	0	0	0	0	7.2536	0	12.6125	0	0	4
CSTA	0	0	0	0	0	1.7225	0	0		2.9429	0	1.9527	4
CTSE	0	0	0	0	8.8218	11.3723	0	0	0	7.2579	0	9.0322	4
DENND2D	0	1.9745	0	1.4693	0	1.4254	0	1.3892	0	0	0	0	4
DOK2	0	2.247	0	0	0	1.7935	0	0	0	2.4488	0	2.773	4
EPS8L3	0	0	0	0	0	69.3278	0	150.466	28.8184	0	0	59.4416	4
FUT6	0	10.1949	2.407	0	0	0	0	4.3842	0	0	0	4.4478	4
FXYD4	0	3.9111	0	0	0	0	0	20.9326	0	46.7958	0	9.1557	4
GPT	3.835	0	0	0	0	0	59.9112	4.2995	0	0	0	3.4581	4
IFITM1	0	0	0	0	1.8534	1.6718	0	0	0	0	2.063	2.686	4
INSL6	0	3.6669	0	0	3.3451	0	0	11.3403	0	9.0925	0	0	4
KAZALD1	0	0	0	3.1457	2.0584	0	0	0	0	5.4544	0	4.4643	4
KCNJ11	0	0	0	0	0	1.6247	0	2.7419	2.1873	0	0	1.5588	4
KRT80	0	2.2625	0	0	0	2.4138	0	2.0644	0	2.7437	0	0	4
LGALS4	0	0	0	0	8.2793	9.2205	0	120.2012	4.7111	0	0	0	4
LPIN2	0	1.472	0	0	0	0	0	2.1658	0	0	1.7583	1.6878	4
MIA	0	0	0	0	0	0	0	15.7445	7.632	2.3467	0	6.3813	4
MKRN3	0	0	4.4771	0	0	0	0	20.7977	0	0	8.2138	13.4692	4
NCF2	0	0	1.8975	0	0	1.7056	0	0	0	2.262	0	2.4617	4
NCF4	0	1.6266	0	1.8304	0	0	0	0	0	1.633	0	1.7371	4
NLRP2	0	0	0	0	11.0737	0	0	9.3935	6.5057	76.981	0	0	4
PPP1R14D	0	0	0	0	0	5.4107	0	16.275	24.9266	14.3425	0	0	4
PTPN22	0	0	0	2.1103	0	1.8762	0	0	0	2	0	2.3324	4
PTPN7	0	3.6539	0	0	0	2.9274	0	0	0	2.7115	0	2.5398	4
RAPSN	0	6.9619	0	0	0	0	0	0	2.9307	10.1582	0	4.0258	4
RDH5	0	0	0	1.7611	0	2.3545	0	2.1848	1.3237	0	0	0	4
RLBP1	0	0	0	3.9062	8.5163	0	0	3.9653	6.8596	0	0	0	4
S100A5	0	0	0	0	0	0	0	22.188	8.2585	2.5264	0	8.2772	4
SERPINF1	0	0	0	0	2.4862	3.4746	0	2.3506	2.0499	0	0	0	4
SLC28A2	0	0	8.356	0	0	0	0	42.1183	0	13.7516	0	3.2214	4
SNX20	0	2.0826	2.68	0	0	0	0	0	0	2.3362	0	2.5418	4
TFF1	0	104.9901	5.3629	0	0	0	0	0	0	0	5.704	31.1738	4
TMEM45B	4.1201	0	1.451	0	0	0	0	2.0149	3.5822	0	0	0	4
TRAF1	3.1898	0	1.3836	0	0	0	0	0	0	1.4272	0	1.832	4

UGT1A10	0	0	0	0	57.6587	142.3124	0	42.3302	198.1796	0	0	0	4
AGMAT	0	0	0	0	1.5989	0	2.0819	0	0	1.5507	0	0	3
ALPPL2	0	0	14.434	0	0	0	0	20.2424	0	15.0429	0	0	3
ARL14	0	12.0545	0	0	0	0	0	10.7269	8.0143	0	0	0	3
BEST3	0	18.9104	0	2.5133	0	0	0	0	0	0	0	9.3527	3
C12orf40	0	6.0689	0	0	4.1554	0	0	0	6.0725	0	0	0	3
CAPN9	0	0	8.9107	0	0	0	0	0	0	3.3255	4.5585	0	3
CARD14	0	0	2.2606	0	0	4.368	0	4.5761	0	0	0	0	3
CASP8	0	0	0	0	0	1.5513	0	1.4092	0	0	0		3
CCDC109B	0	1.5034	0	1.8846	0	1.5709	0	0	0	0	0	0	3
CCL5	0	4.0381	0	0	0	0	0	0	0	4.0877	0	2.3578	3
CCND1	0	0	0	3.0421	0	0	2.6178	0	0	0	0	1.8962	3
CD5	0	0	0	0	0	2.7754	0	0	0	2.1563	0	2.6085	3
CD6	0	0	0	1.8126	0	2.6068	0	0	0	0	0	3.1256	3
CDSN	0	0	0	0	0	2.2283	0	0	0	2.4806	2.5858	0	3
CHRNA9	0	29.1054	0	0	0	0	0	118.834	16.6261	0	0	0	3
CHST6	0	2.6204	0	0	0	0	0	2.5236	2.192	0	0	0	3

Supplementary Table 8

Stem cell gene set	BLCA	BRCA	COAD	GBM	HNSC	KIRC	LAML	LUAD	LUSC	OV	READ	UCEC	Fisher Combo	ChiSquare Pvalue	ChiSquare Pvalue-FDR
Boyer_Cobound_Oct4_Sox2_Nanog_unexpressed	0.00630	0.00009	0.00020		0.00000		0.00670	0.00040	0.00000		0.05459		45.494	0	0
Ivanova_Tcl_shRNA	0.00554	0.00000	0.00226		0.00363		0.00004	0.00083	0.00836	0.00030	0.03540	0.00000	75.3577	0	0
Ivanova_Up_Pattern3	0.00066	0.00001		0.00227	0.00683	0.00006	0.00073	0.00001	0.04300	0.00473		0.00006	60.4623	0	0
Kim_Cell200_PrC_Human	0.00000	0.00007	0.00007	0.00267	0.00000	0.03080	0.06200	0.00000	0.00000			0.00001	38.853	0	0
Kim_Cell200_PrC_Module	0.00001	0.00006	0.00002	0.00229	0.00000	0.02790	0.05530	0.00000	0.00000		0.04846	0.00004	32.398	0	0
Kim_Cell200_Smad	0.02900	0.00029	0.00001		0.00048		0.06882	0.00007	0.00002	0.00457	0.00328	0.04374	4.493	0	0
Kim_Cell200_Zfp28	0.00033	0.00061	0.00000		0.00587		0.00070	0.00008	0.00737		0.02500	0.00003	43.52	0	0
Loh_Nanogbind_diffexp	0.02759	0.00470	0.00000	0.00284	0.00530		0.00060	0.00060		0.04908	0.00053		34.564	0	0
Morris_HFSC_dChipUp		0.00003	0.00450	0.00048	0.02942	0.00250	0.00092	0.00762			0.02379	0.00000	27.566	4.44E-06	2.08E-15
Ivanova_Down_Nanog_shRNA_TC	0.00976	0.00940	0.00044		0.00027	0.03744	0.00004	0.00945	0.02085		0.00045	0.03490	20.9804	6.44E-15	2.73E-14
Kim_Zfp28_targets	0.00839	0.00649	0.00001		0.00256		0.00022	0.00054	0.03351		0.02287	0.00725	6.5584	3.96E-14	5.34E-13
Ivanova_Up_Pattern2	0.03829	0.00007	0.00004	0.00298	0.03923		0.04000	0.04235	0.04703	0.04089	0.00943	0.00774	3.3672	4.49E-13	5.58E-13
LauAllPrognosisGenes	0.00009	0.00234	0.00009		0.00860		0.02623	0.00777	0.02645	0.00721	0.00286		3.485	4.89E-13	5.58E-13
Ivanova_Down_Dppa4_shRNA	0.00742	0.00001	0.03004		0.00048			0.00200	0.02749	0.00281	0.02830	0.00338	6.786	2.04E-12	7.00E-12
Mathur_Oct4KO_Down	0.04506	0.00005	0.00073				0.02981	0.00001	0.02491	0.02509	0.00410	0.04838	5.9066	2.90E-12	9.57E-12
Garber_MolecularClassificationSignature		0.00001	0.00001		0.00004	0.03082		0.03649		0.09580	0.03935		98.5877	5.23E-11	6.60E-10
Mikkelsen_Up_partial-iPS-ES	0.00000	0.02900	0.08242		0.00285	0.02820	0.00003		0.08354			0.03809	97.9244	6.78E-11	2.08E-10
Morris_HFSC_GenespringUp		0.00001	0.03303		0.00427			0.00005	0.00565		0.02504	0.00008	97.7962	7.95E-11	2.20E-10
Morris_HFSC_dChip_Down	0.00031	0.00084	0.00367				0.00541	0.05549	0.00864		0.00006	0.04489	97.3689	8.41E-11	2.45E-10
BreastVsBreast4	0.00726	0.00389	0.00765	0.03685	0.00085		0.00041	0.02570	0.06699			0.00229	92.4744	5.59E-10	4.64E-09
Mathur_Nanog4KO_Down		0.00680	0.00008					0.00001	0.00988	0.00024		0.00702	87.25	4.46E-09	2.49E-09
Morris_HFSC_combined_Up		0.00002	0.05525		0.05354			0.00306	0.00720		0.02740	0.00041	85.7677	7.49E-09	7.96E-08
Karsten_Up_Differentiated	0.00020	0.00270			0.00826			0.02977	0.03373	0.00321		0.00002	85.72	9.24E-09	2.37E-08
Mathur_Nanogbound-bound	0.02855		0.00440	0.02608	0.00070		0.03630	0.00009	0.04376	0.00678			82.7283	2.22E-08	5.07E-08
Sato_HSC_enriched_Differentiated_absent_SOURCE	0.05728	0.00270			0.00025		0.00257	0.00504	0.00084			0.03500	80.35	5.79E-08	2.57E-07
Morris_HFSC_combined_Down	0.03230	0.00849	0.00588			0.03963	0.03632			0.00690	0.00081	0.05847	80.094	6.04E-08	2.80E-07
Roepman_LungCancerPrognosisSignature			0.07405		0.04334	0.02224	0.00223		0.02631	0.02900	0.00354	0.00020	78.7939	9.46E-08	9.33E-07
Boyer_ExpressedBound_Oct4_Cell2005		0.00246	0.00745		0.00040		0.00304	0.00551			0.02937	0.00876	78.2658	4.67E-07	2.27E-07
Ivanova_OverExpressionRegulators	0.00633	0.05396	0.00475		0.00344		0.00056	0.00751		0.02887			74.922	3.84E-07	7.42E-07
Fortunel_NPC_3studies		0.00007		0.08495	0.03027	0.03820			0.00492	0.06755		0.00544	72.0375	6.99E-07	2.03E-06
Chen_Sox2_geneassociations			0.03858	0.02330	0.04090			0.00270		0.00002	0.02426	0.03983	69.8554	2.30E-06	4.09E-06
Kosinski_Up_ColonCrypts		0.02828	0.00076		0.07435		0.04737	0.04741		0.00056		0.00256	68.723	3.42E-06	5.97E-06
Lu_LungCancerSurvivalSignature	0.00741	0.00034	0.05405		0.02008			0.00949	0.00266				66.2648	7.93E-06	3.57E-05
Hu_Her2Genes_SuppTable	0.00082		0.03780			0.03586		0.06392		0.00600	0.03638	0.09499	65.583	5.38E-05	9.38E-05
Kim_Cell200_Core_Human			0.00029		0.00780			0.00048	0.04700	0.09500	0.03770		64.8224	2.92E-05	2.30E-05
Morris_HFSC_Genespring_Down	0.04897	0.07959	0.00373			0.02304				0.07400	0.00046	0.02890	64.466	6.22E-05	2.63E-05
Komor_UpMegakaryopoieticDiff		0.00807					0.09240	0.00792		0.06073	0.00573	0.00047	64.0924	6.52E-05	2.63E-05
Schebesta_Pax5_activated	0.02966	0.00269	0.00044		0.00406			0.02304			0.00606		63.7069	8.80E-05	2.94E-05
Kim_Cell200_Core_Module			0.00081		0.00450			0.00026	0.02509	0.00068			63.532	9.93E-05	3.06E-05
VeneziaHSC_Qsig		0.00628	0.00443			0.00257	0.00022		0.02026	0.04338			63.295	2.60E-05	3.26E-05
Ivanova_Down_Oct4_shRNA_TC		0.00534		0.00701	0.04928		0.00274	0.00380				0.03693	6.56	3.90E-05	5.68E-05
Ivanova_Up_Oct4_shRNA_TC		0.00534		0.00701	0.04928		0.00274	0.00380				0.03693	6.56	3.90E-05	5.68E-05
Chitale_EGFRsignature		0.00650				0.00995	0.00634	0.06262	0.00827	0.04406		0.02230	6.583	4.38E-05	6.28E-05
Ben-Porath_Core9genes	0.00067	0.00443			0.02682			0.00406	0.03904			0.00398	59.449	7.65E-05	0.000048
Bracken_Polycomb-depleted_Up		0.00238	0.04824		0.02088		0.03270	0.00200	0.00774				56.796	0.000266	0.0002835
Ivanova_Down_Pattern2	0.04295	0.00885	0.03629		0.00488			0.00094			0.03875		55.56	0.00026288	0.00033908
p9nullvMYC_UNI_DN	0.00939	0.03903			0.00540			0.07858	0.00289		0.03830		52.5883	0.00065532	0.0008332
Kim_birA_targets		0.00002						0.00020	0.00255				5.3287	0.00095632	0.00988
Chen_Smad_geneassociations			0.00046		0.03300			0.04960	0.08073	0.00361			49.8582	0.00476	0.008204
p9nullvMYC_UNI_TOT		0.02238			0.00827			0.02395	0.00076			0.05930	49.887	0.00493	0.008204
Komor_UpErythropoieticDiff					0.00731	0.03440	0.02758	0.00386	0.04250			0.04368	49.3328	0.0072	0.0020688

Ben-Porath_ES-ExprSet	0.03400		0.00688					0.00352	0.02059	0.02075			48.62	0.0024	0.0028224
NKX22_038_Array2	0.03448		0.03867	0.03934	0.00992			0.00359	0.02236				47.7989	0.0026729	0.0030894
Fortunel_ESC_3studies			0.00489		0.02627			0.00273	0.02099		0.03775		46.4809	0.0038735	0.004498
BeerAdenoPrognosisSignature	0.02823		0.00847	0.00666				0.07400			0.03062		44.3668	0.006945	0.0077898
Mikkelsen_Down_iPS-ES				0.03969	0.00535		0.09700		0.00349			0.04071	43.4947	0.0087292	0.00973

Supplementary Table 9

	DM value clustering			Beta value clustering		
	Intra-cluster consensus	Inter-Cluster consensus		Intra-cluster consensus	Inter-Cluster consensus	Jaccard coefficient
BLCA	80%	8%	BLCA	74%	11%	0.34
BRCA	89%	4%	BRCA	84%	6%	0.41
COAD	96%	3%	COAD	88%	6%	0.67
GBM	89%	5%	GBM	84%	7%	0.44
HNSC	86%	5%	HNSC	77%	8%	0.33
KIRC	91%	4%	KIRC	87%	5%	0.29
LAML	89%	8%	LAML	74%	19%	0.30
LUAD	77%	8%	LUAD	80%	8%	0.24
LUSC	75%	9%	LUSC	69%	10%	0.30
OV	93%	4%	OV	93%	4%	0.31
READ	65%	12%	READ	62%	9%	0.20
UCEC	91%	3%	UCEC	87%	6%	0.37

All green values correspond to clusterings where the DM value clustering is better than clustering the beta-values.
This means higher consensus within a each cluster (=intra cluster consensus) and lower consensus for samples clustered in different clusters (=inter cluster consensus)
For both clusterings exactly the same number of clusters where compared for each cancer

The jaccard coefficient is the comparison fo the DM-value clustering and the beta value clustering.

Supplementary Table 10

Colon cancer (COAD)

DM value clustering

		BRAF mutation absent	BRAF mutation Present
CIMP vs. BRAF			
	non-CIMP-cluster	126	0
	CIMP-Cluster	28	**7**
Pvalue	0.00001		
CIMP-cluster vs. CIMP-High		Not CIMP-High	CIMP-High
	non-CIMP-cluster	123	3
	CIMP-Cluster	6	**29**
Pvalue	5.83E-07		
CIMP-cluster vs. MLH1silencing		no MLH1-silencing	MLH1-silencing
	non-CIMP-cluster	124	2
	CIMP-Cluster	10	**25**
Pvalue	8.65E-06		

Beta value clustering

	CIMP vs. BRAF	BRAF mutation absent	BRAF mutation Present
CIMP vs. BRAF			
	non-CIMP-cluster	132	5
	CIMP-Cluster	22	**2**
Pvalue	0.2115		
CIMP-cluster vs. CIMP-High		Not CIMP-High	CIMP-High
	non-CIMP-cluster	126	11
	CIMP-Cluster	3	**21**
Pvalue	8.85E-07		
CIMP-cluster vs. MLH1silencing		no MLH1-silencing	MLH1-silencing
	non-CIMP-cluster	130	7
	CIMP-Cluster	4	**20**
Pvalue	2.91E-06		

Acute Myeloid Leukemia (LAML)

DM value clustering

		IDHx mutation absent	IDHx mutation present
CIMP vs. IDHx			
	non-CIMP-cluster	136	10
	CIMP-Cluster	19	**26**
Pvalue	7.54E-06		

Beta value clustering

		IDHx mutation absent	IDHx mutation present
CIMP vs. IDHx			
	non-CIMP-cluster	136	17
	CIMP-Cluster	19	**19**
Pvalue	3.15E-06		

Glioblastoma multiforme (GBM)

DM value clustering

		Not CIMP	CIMP
	non-CIMP-cluster	207	5
	CIMP-Cluster	0	**18**
Pvalue	8.42E-07		

Beta value clustering

		Not CIMP	CIMP
	non-CIMP-cluster	207	5
	CIMP-Cluster	0	**18**
Pvalue	8.42E-07		

Supplementary Table 11

Colon cancer (COAD)

DM value clustering

CIMP vs. BRAF		BRAF mutation absent	BRAF mutation Present
	non-CIMP-cluster	126	0
	CIMP-Cluster	28	**7**
Pvalue	0.00001		
CIMP-cluster vs. CIMP-High		Not CIMP-High	CIMP-High
	non-CIMP-cluster	123	3
	CIMP-Cluster	6	**29**
Pvalue	5.83E-07		
CIMP-cluster vs. MLH1silencing		no MLH1-silencing	MLH1-silencing
	non-CIMP-cluster	124	2
	CIMP-Cluster	10	**25**
Pvalue	8.65E-06		

RPMM beta value clustering

CIMP vs. BRAF	CIMP vs. BRAF	BRAF mutation absent	BRAF mutation Present
	not Cluster-8	143	4
	Cluster-8*	11	**3**
Pvalue	0.0139		
CIMP-cluster vs. CIMP-High		Not CIMP-High	CIMP-High
	not Cluster-5	129	20
	Cluster-5*	0	**12**
Pvalue	2.74E-06		
CIMP-cluster vs. MLH1silencing		no MLH1-silencing	MLH1-silencing
	not Cluster-5	134	15
	Cluster-5*	0	**12**
Pvalue	1.68E-06		

*The cluster with the highest enrichment of BRAF, CIMP-high and MLH1-silencing was selected
None of the RPMM clusters captures the complete COAD-CIMP-High pattern, BRAF mutation and MLH1-silencing

Acute Myeloid Leukemia (LAML)

DM value clustering

CIMP vs. IDHx		IDHx mutation absent	IDHx mutation present
	non-CIMP-cluster	136	10
	CIMP-Cluster	19	**26**
Pvalue	7.54E-06		

Beta value clustering

CIMP vs. IDHx		IDHx mutation absent	IDHx mutation present
	non-CIMP-cluster	151	14
	CIMP-Cluster	4	**22**
Pvalue	6.47E-06		

Glioblastoma multiforme (GBM)

DM value clustering

		Not CIMP	CIMP
	non-CIMP-cluster	207	5
	CIMP-Cluster	0	**18**
Pvalue	8.42E-07		

Beta value clustering

		Not CIMP	CIMP
	not Cluster-5	207	12
	Cluster-5	0	**11**
Pvalue	8.42E-07		

Supplementary Table 12

	DM value clustering			Gene expression clustering		
	Intra-cluster consensus	Inter-Cluster consensus		Intra-cluster consensus	Inter-Cluster consensus	Jaccard coefficient
BLCA	80%	8%	BLCA	76%	12%	0.23
BRCA	89%	4%	BRCA	94%	4%	0.30
COAD	96%	3%	COAD	92%	9%	0.37
GBM	89%	5%	GBM	72%	13%	0.24
HNSC	86%	5%	HNSC	77%	5%	0.23
KIRC	91%	4%	KIRC	76%	9%	0.25
LAML	89%	8%	LAML	71%	15%	0.28
LUAD	77%	8%	LUAD	81%	9%	0.22
LUSC	75%	9%	LUSC	77%	11%	0.25
OV	93%	4%	OV	71%	14%	0.26
READ	65%	12%	READ	71%	15%	0.21
UCEC	91%	3%	UCEC	82%	7%	0.26

All green values correspond to clusterings where the DM value clustering is better than clustering gene expression values
This means higher consensus within a each cluster (=intra cluster consensus) and lower consensus for samples clustered in different clusters (=inter cluster consensus)
For both clusterings exactly the same number of clusters where compared for each cancer

The jaccard coefficient is the comparison fo the DM-value clustering and the gene expression clustering.

Supplementary Table 13

Pancancer cluster	BLCA	BRCA	COAD	GBM	HNSC	KIRC	LAML	LUAD	LUSC	OV	READ	UCEC
Pancancer cluster C1	2	0	408	0	0	0	0	4	0	0	161	0
Pancancer cluster C2	61	32	4	1	36	3	0	155	263	2	1	11
Pancancer cluster C3	40	4	2	0	0	10	0	261	24	1	0	18
Pancancer cluster C4	0	0	0	397	0	0	0	0	0	0	0	0
Pancancer cluster C5	2	5	0	2	0	2	194	2	2	0	0	1
Pancancer cluster C6	0	15	0	2	1	22	0	2	4	578	0	87
Pancancer cluster C7	0	0	0	0	1	463	0	3	0	1	0	0
Pancancer cluster C8	17	0	0	0	272	0	0	3	65	0	0	0
Pancancer cluster C9	1	0	0	0	0	0	0	0	0	2	0	383
Pancancer cluster C10	0	257	0	0	0	0	0	0	0	0	0	0

Supplementary Table 14

Pancancer cluster 1

Geneset	Pvalue	FDR	NrGeneOverlap	GeneOverlap
REACTOME_GLUCAGON_TYPE_LIGAND_RECEPTORS	2.31E-07	6.67E-06	12	ADCYAP1//GCGR//GHRH//GHRHR//GLP2R//GNG13//GNG4//GNG8//GNGT1//SCT//VIP//VIPR2
Glucagon-type ligand receptors	2.31E-07	1.38E-05	12	ADCYAP1//GCGR//GHRH//GHRHR//GLP2R//GNG13//GNG4//GNG8//GNGT1//SCT//VIP//VIPR2
CARBONATE_DEHYDRATASE_ACTIVITY	3.47E-06	6.68E-05	7	CA1//CA12//CA4//CA6//CA7//CA8//CA9
KEGG_TYROSINE_METABOLISM	4.35E-06	4.54E-05	12	ADH1A//ADH1B//ADH1C//ADH4//ADH6//ALDH3A1//DDC//HGD//TH//TPO//TYR//TYRP1
BIOCARTA_STEM_PATHWAY	1.17E-05	0.0024544	7	CSF2//CSF3//IL11//IL4//IL5//IL6//IL8
LIPID_METABOLIC_PROCESS	1.57E-05	0.00025237	39	AKR1B10//AKR1C2//AKR1C4//APOA5//APOBEC1//APOC3//BAAT//BTNL3//CEL//CPNE6//CYP3A4//CYP3A5//CYP4F2//CYP4F3//CYP4F8//DHRS2//HNF4A//HPGD//HSD3B1//IL4//MTTP//NPC1L1//NR0B2//NR1H4//NR1I2//PLA1A//PLA2G3//PPARGC1A//PTGES//RBP3//SHH//ST8SIA3//SULT2A1//SULT2B1//TTPA//UGT1A1//UGT2B10//UGT2B15//UGT2B7
OXIDOREDUCTASE_ACTIVITY_GO_0016616	2.11E-05	0.0003309	13	ADH1B//ADH4//ADH6//AKR1B10//AKR1C4//AKR7A3//ALDH3A1//DHRS2//GPD1//HPGD//HSD3B1//HSD3B2//RDH8
CHANNEL_REGULATOR_ACTIVITY	2.50E-05	0.0003865	8	CHRNA7//KCNV1//NPY//NPY2R//PDZD3//SGK2//TMPRSS3//TNNI3
REGULATION_OF_BLOOD_PRESSURE	2.50E-05	0.00038154	8	AGT//CALCA//CARTPT//CHGA//GCGR//NPPA//REN//UTS2
REACTOME_REGULATION_OF_INSULIN_LIKE_GROWTH_FACTOR_ACTIVITY_BY_INSULIN_LIKE_GROWTH_FACTOR_BINDING_PROTEINS	3.18E-05	0.00045855	7	F2//IGF2//IGFALS//IGFBP1//KLK3//MMP1//PLG
Regulation of Insulin-like Growth Factor (IGF) Activity by Insulin-lik	3.18E-05	0.0011757	7	F2//IGF2//IGFALS//IGFBP1//KLK3//MMP1//PLG
SERINE_TYPE_PEPTIDASE_ACTIVITY	3.43E-05	0.00049887	11	ACR//F11//F2//F7//KLK1//KLK8//PCSK9//PLG//PRSS1//PRSS3//TMPRSS6
Digestion of dietary carbohydrate	3.74E-05	0.0013367	5	AMY1A//AMY2A//LCT//SI//TREH
SERINE_HYDROLASE_ACTIVITY	4.33E-05	0.00061426	11	ACR//F11//F2//F7//KLK1//KLK8//PCSK9//PLG//PRSS1//PRSS3//TMPRSS6
REACTOME_REGULATION_OF_BETA_CELL_DEVELOPMENT	4.79E-05	0.00066475	19	FGF10//FOXA2//FOXA3//HNF1A//HNF1B//HNF4A//HNF4G//IAPP//INSM1//NEUROD1//NEUROG3//NKX2-2//ONECUT3//PDX1//PKLR//PTF1A//RPL21//RPS28//RPS4Y1
AUXILIARY_TRANSPORT_PROTEIN_ACTIVITY	5.16E-05	0.00069811	8	CHRNA7//KCNV1//NPY//NPY2R//PDZD3//SGK2//TMPRSS3//TNNI3
WOUND_HEALING	5.67E-05	0.00075086	12	APOA5//C4BPB//EREG//F2//F7//GP9//HNF4A//KLK8//KNG1//PF4//PLG//TMPRSS6
OXIDOREDUCTASE_ACTIVITY__ACTING_ON_CH_OH_GROUP_OF_DONORS	6.63E-05	0.00086747	13	ADH1B//ADH4//ADH6//AKR1B10//AKR1C4//AKR7A3//ALDH3A1//DHRS2//GPD1//HPGD//HSD3B1//HSD3B2//RDH8

Pancancer cluster 2

Geneset	Pvalue	FDR	NrGeneOverlap	GeneOverlap
AMINE_TRANSMEMBRANE_TRANSPORTER_ACTIVITY	9.40E-07	1.25E-05	14	OCA2//PDPN//SLC18A3//SLC1A6//SLC1A7//SLC22A3//SLC5A7//SLC6A14//SLC6A2//SLC6A3//SLC6A4//SLC7A10//SLC7A2//SLC7A4
MALE_GONAD_DEVELOPMENT	7.81E-06	9.08E-05	7	ANKRD7//DMRT1//DMRT2//LHB//NR5A1//SOX15//SRD5A2
syndecan_1_pathway	2.38E-05	0.0048862	13	COL10A1//COL11A1//COL17A1//COL1A1//COL2A1//COL4A3//COL4A6//COL7A1//COL8A1//COL9A3//MET//MMP1//MMP7
G_PROTEIN_SIGNALING__ADENYLATE_CYCLASE_ACTIVATING_PATHWAY	5.26E-05	0.00053248	9	ADCYAP1//ADRB3//CALCA//CALCR//CRHR1//DRD1//DRD5//GLP1R//PTHLH
integrin1_pathway	9.46E-05	0.0096978	15	COL11A1//COL1A1//COL2A1//COL4A3//COL4A6//COL7A1//FGA//FGB//FGG//ITGA8//LAMA1//LAMA3//LAMB3//LAMC2//SPP1

Pancancer cluster 3

Geneset	Pvalue	FDR	NrGeneOverlap	GeneOverlap

IMMUNE_SYSTEM_PROCESS	1.54E-10	4.67E-09	51	AZU1//CALCA//CCL18//CCL19//CCL20//CCL21//CCL23//CCL24//CCL25//CCL26//CCR8//CCR9//CD79A//CEACAM8//CHST4//CNR2//CTSE//CTSG//CXCL13//DMBT1//EREG//FCAR//HAMP//IL12B//IL17A//IL2//IL21//IL27//IL29//IL31RA//IL6//IL8//INHA//KIR2DL1//KIR2DL3//LTF//MADCAM1//MNX1//MS4A1//MS4A2//NCR1//OPRK1//PAX5//PF4//SAA1//SFTPD//SPACA3//SPINK5//TBX1//TRAT1//ZBTB16
IMMUNE_RESPONSE	2.74E-09	6.43E-08	39	CCL18//CCL19//CCL20//CCL21//CCL23//CCL24//CCL25//CCL26//CCR8//CCR9//CD79A//CEACAM8//CHST4//CNR2//CTSG//CXCL13//DMBT1//EREG//FCAR//HAMP//IL12B//IL17A//IL2//IL27//IL29//IL6//KIR2DL1//KIR2DL3//LTF//MADCAM1//MNX1//MS4A1//MS4A2//NCR1//OPRK1//PAX5//SFTPD//SPINK5//TRAT1
KEGG_ETHER_LIPID_METABOLISM	1.59E-06	2.08E-05	11	PLA2G10//PLA2G12B//PLA2G1B//PLA2G2A//PLA2G2C//PLA2G2D//PLA2G2F//PLA2G3//PLA2G4E//PLA2G5//PPAP2C
CALCIUM_INDEPENDENT_CELL_CELL_ADHESION	1.99E-06	3.01E-05	9	CLDN1//CLDN10//CLDN18//CLDN2//CLDN3//CLDN4//CLDN6//CLDN8//CLDN9
NEGATIVE_REGULATION_OF_CELL_DIFFERENTIATION	2.01E-05	0.00025058	9	CALCA//CNTN4//EREG//GPR98//INHA//PF4//SHH//TWIST2//ZBTB16
NEGATIVE_REGULATION_OF_CELL_PROLIFERATION	3.06E-05	0.00036623	23	ALOX15B//BNIPL//BTG4//CCL23//CCL3L3//COL4A3//CXCL1//EREG//FABP6//FGFBP1//IL1A//IL29//IL6//IL8//KRT4//MYO16//NOX4//RERG//SFTPD//SSTR1//SSTR3//TBX5//TM4SF4
hnf3apathway	3.47E-05	0.0072061	11	C4BPB//CYP2C18//FOXA1//FOXA2//FOXA3//SCGB1A1//SFTPA1//SFTPA2//SFTPD//SHH//TFF1
REACTOME_TIGHT_JUNCTION_INTERACTIONS	3.73E-05	0.00098973	9	CLDN1//CLDN10//CLDN18//CLDN2//CLDN3//CLDN4//CLDN6//CLDN8//CLDN9
Tight junction interactions	3.73E-05	0.0020041	9	CLDN1//CLDN10//CLDN18//CLDN2//CLDN3//CLDN4//CLDN6//CLDN8//CLDN9
KEGG_LONG_TERM_DEPRESSION	3.97E-05	0.0004384	14	CRH//GRIA1//GRM5//PLA2G10//PLA2G12B//PLA2G1B//PLA2G2A//PLA2G2C//PLA2G2D//PLA2G2F//PLA2G3//PLA2G4E//PLA2G5//PRKG2
PHOSPHOLIPASE_A2_ACTIVITY	4.84E-05	0.0005563	6	PLA2G10//PLA2G1B//PLA2G2A//PLA2G2D//PLA2G3//PLA2G5
TIGHT_JUNCTION	4.99E-05	0.00056219	9	CLDN1//CLDN10//CLDN18//CLDN2//CLDN3//CLDN4//CLDN6//CLDN8//CLDN9
a6b1_a6b4_integrin_pathway	5.42E-05	0.0056244	11	COL17A1//EGF//EGFR//IL1A//LAMA3//LAMB3//LAMC2//MET//MST1R//RXRG//SFN
KEGG_BASAL_CELL_CARCINOMA	6.62E-05	0.00063374	12	BMP2//HHIP//SHH//WNT1//WNT10A//WNT16//WNT2//WNT3A//WNT7A//WNT7B//WNT9A//WNT9B
CELL_MIGRATION	9.50E-05	0.001011	16	ALOX15B//AZU1//CALCA//CNTN4//EGFR//IL12B//IL8//PF4//PRSS3//S100A2//S100P//SAA1//SFTPD//SHH//TBX5//WNT1

Pancancer cluster 4

Geneset	Pvalue	FDR	NrGeneOverlap	GeneOverlap
GABA A receptor activation	8.91E-11	5.96E-09	10	GABRA1//GABRA2//GABRA3//GABRA4//GABRA5//GABRB1//GABRB2//GABRB3//GABRG2//GABRG3
REACTOME_NEURORANSMITTER_RECEPTOR_BINDING_AND_DOWNSTREAM_TRANSMISSION_IN_THE_POSTSYNAPTIC_CELL	8.98E-11	4.93E-09	25	ACTN2//ADCY8//CACNG2//CACNG3//CACNG4//CACNG8//CAMK2A//CAMK2B//GNG13//GNG4//GNG8//GRIA1//GRIA2//GRIA3//GRIA4//GRIK1//GRIK2//GRIK3//GRIK4//GRIK5//GRIN1//GRIN2A//GRIN2B//NEFL//PRKCG
Neurotransmitter Release Cycle	2.69E-08	1.44E-06	14	CHAT//GAD1//GAD2//RIMS1//SLC18A3//SLC1A6//SLC1A7//SLC32A1//SLC6A11//SLC6A13//SNAP25//SYN2//SYN3//SYT1
Developmental Biology	3.99E-07	2.03E-05	56	ANGPTL4//CACNA1G//CACNA1S//CDH15//CDH2//CDH4//CER1//CHL1//CNTN1//CNTN2//CNTN6//COL2A1//COL9A1//COL9A3//CTNNA2//DCC//DCX//DPYSL5//EGFR//GCK//GDF1//HNF4G//INSM1//KCNQ2//KCNQ3//L1CAM//LAMA1//LEFTY1//LEFTY2//MAFA//MYOG//NCAN//NEUROD1//NEUROG3//NKX2-2//NKX6-1//NRCAM//NTN3//ONECUT1//PAK3//PAK7//PLIN1//PPARGC1A//PSPN//ROBO2//SCN1A//SCN2A//SCN2B//SCN7A//SEMA3E//SH3GL2//ST8SIA2//TRPC5//TRPC7//UNC5A//UNC5D
VOLTAGE_GATED_CALCIUM_CHANNEL_ACTIVITY	6.54E-07	1.15E-05	9	CACNA1B//CACNA1E//CACNA1G//CACNA1S//CACNA2D1//CACNG1//CACNG2//CACNG4//CACNG5
REACTOME_ADHERENS_JUNCTIONS_INTERACTIONS	1.14E-06	4.38E-05	11	CADM2//CADM3//CDH10//CDH15//CDH18//CDH2//CDH4//CDH6//CDH7//CDH8//CDH9

Adherens junctions interactions	1.14E-06	5.30E-05	11	CADM2//CADM3//CDH10//CDH15//CDH18//CDH2//CDH4//CDH6//CDH7//CDH8//CDH9
REACTOME_TRAFFICKING_OF_AMPA_RECEPTORS	1.69E-06	5.42E-05	11	CACNG2//CACNG3//CACNG4//CACNG8//CAMK2A//CAMK2B//GRIA1//GRIA2//GRIA3//GRIA4//PRKCG
Glutamate Binding, Activation of AMPA Receptors and Synaptic Plasticit	1.69E-06	7.24E-05	11	CACNG2//CACNG3//CACNG4//CACNG8//CAMK2A//CAMK2B//GRIA1//GRIA2//GRIA3//GRIA4//PRKCG
Trafficking of AMPA receptors	1.69E-06	6.96E-05	11	CACNG2//CACNG3//CACNG4//CACNG8//CAMK2A//CAMK2B//GRIA1//GRIA2//GRIA3//GRIA4//PRKCG
REACTOME_ACTIVATION_OF_NMDA_RECEPTOR_UPON_GLUTAMATE_BINDING_AND_POSTSYNAPTIC_EVENTS	1.85E-06	5.08E-05	12	ACTN2//ADCY8//CAMK2A//CAMK2B//GRIA1//GRIA2//GRIA3//GRIA4//GRIN1//GRIN2A//GRIN2B//NEFL
Activation of NMDA receptor upon glutamate binding and postsynaptic ev	2.58E-06	9.84E-05	12	ACTN2//ADCY8//CAMK2A//CAMK2B//GRIA1//GRIA2//GRIA3//GRIA4//GRIN1//GRIN2A//GRIN2B//NEFL
ACETYLCHOLINE_BINDING	5.01E-06	8.13E-05	8	CHRM1//CHRM2//CHRM3//CHRNA1//CHRNA2//CHRNA4//CHRNA6//CHRNA7
REACTOME_NEUROTRANSMITTER_RELEASE_CYCLE	6.65E-06	0.00015978	10	CHAT//RIMS1//SLC18A3//SLC1A6//SLC1A7//SNAP25//SYN2//SYN3//SYT1//TPH1
GABA synthesis, release, reuptake and degradation	1.38E-05	0.00046215	8	GAD1//GAD2//RIMS1//SLC32A1//SLC6A11//SLC6A13//SNAP25//SYT1
Activation of AMPA receptors	1.89E-05	0.00061195	4	GRIA1//GRIA2//GRIA3//GRIA4
BRAIN_DEVELOPMENT	1.92E-05	0.00029252	13	ALK//DLX2//DMBX1//DSCAML1//FOXG1//MYO16//NKX2-2//OTX2//ROBO2//SIX3//TBR1//ZIC1//ZIC2
KEGG_ARRHYTHMOGENIC_RIGHT_VENTRICULAR_CARDIOMYOPATHY_ARVC	2.09E-05	0.00068333	16	ACTN2//CACNA1S//CACNA2D1//CACNG1//CACNG2//CACNG3//CACNG4//CACNG5//CACNG6//CACNG7//CACNG8//CDH2//CTNNA2//CTNNA3//DES//SGCG
VOLTAGE_GATED_CALCIUM_CHANNEL_COMPLEX	2.16E-05	0.00032165	7	CACNA1B//CACNA1E//CACNA1G//CACNA1S//CACNA2D1//CACNG1//CACNG2
CREB phosphorylation through the activation of CaMKII	2.16E-05	0.00067918	7	ACTN2//CAMK2A//CAMK2B//GRIN1//GRIN2A//GRIN2B//NEFL
CALCIUM_CHANNEL_ACTIVITY	3.46E-05	0.00048733	10	CACNA1B//CACNA1E//CACNA1G//CACNA1S//CACNA2D1//CACNG1//CACNG2//CACNG4//CACNG5//TRPC5
REACTOME_CREB_PHOSPHORYLATION_THROUGH_THE_ACTIVATION_OF_CAMKII	3.62E-05	0.00081816	7	ACTN2//CAMK2A//CAMK2B//GRIN1//GRIN2A//GRIN2B//NEFL
G_PROTEIN_SIGNALING__COUPLED_TO_IP3_SECOND_MESSENGER__PHOSPHOLIPASE_C_ACTIVATING	4.99E-05	0.00067977	11	CCKAR//CCKBR//CHRM1//CHRM2//DRD1//DRD2//EGFR//GAP43//GRM5//NMBR//NMUR2
NEUROTRANSMITTER_SECRETION	5.34E-05	0.00071296	6	CARTPT//HRH3//NLGN1//RIMS1//SYN3//SYT1
Ras activation uopn Ca2+ infux through NMDA receptor	5.80E-05	0.0015911	7	ACTN2//CAMK2A//CAMK2B//GRIN1//GRIN2A//GRIN2B//NEFL
cone_pathway	7.07E-05	0.015383	8	CNGA3//GRK1//GUCA1A//GUCY2F//LRAT//PDE6H//RPE65//SLC24A2
REACTOME_RAS_ACTIVATION_UOPN_CA2+_INFUX_THROUGH_NMDA_RECEPTOR	8.95E-05	0.0018109	7	ACTN2//CAMK2A//CAMK2B//GRIN1//GRIN2A//GRIN2B//NEFL

Pancancer cluster 5

**Geneset**	**Pvalue**	**FDR**	**NrGeneOverlap**	**GeneOverlap**
KEGG_OLFACTORY_TRANSDUCTION	9.68E-12	5.59E-10	50	CALML6//GUCY2D//OR10AD1//OR10G2//OR10H1//OR10H2//OR10H5//OR11H4//OR13A1//OR13D1//OR14I1//OR1G1//OR1J1//OR1J4//OR1K1//OR1L8//OR1N1//OR1Q1//OR2AE1//OR2AG2//OR2B11//OR2B2//OR2B6//OR2C1//OR2C3//OR2H2//OR2K2//OR2L13//OR2L2//OR2T33//OR2T8//OR2W3//OR3A1//OR3A2//OR51B4//OR51B5//OR51Q1//OR52B6//OR52K2//OR52N2//OR52N4//OR52W1//OR56B1//OR56B4//OR5C1//OR5K2//OR7D2//OR9A4//PDC//PRKACG
KEGG_TASTE_TRANSDUCTION	3.75E-10	1.62E-08	16	GNG13//PRKACG//TAS1R1//TAS1R2//TAS2R10//TAS2R13//TAS2R19//TAS2R3//TAS2R31//TAS2R4//TAS2R40//TAS2R42//TAS2R43//TAS2R46//TAS2R50//TAS2R60

REACTOME_OLFACTORY_SIGNALING_PATHWAY	2.19E-09	1.00E-07	44	OR10AD1//OR10G2//OR10H1//OR10H2//OR10H5//OR14I1//OR1F2P//OR1G1//OR1J1//OR1J4//OR1K1//OR1L8//OR1N1//OR1Q1//OR2AE1//OR2AG2//OR2B11//OR2B2//OR2B6//OR2C1//OR2C3//OR2H2//OR2K2//OR2L13//OR2L2//OR2T33//OR2T8//OR2W3//OR3A1//OR3A2//OR4F29//OR51B4//OR51B5//OR51Q1//OR52B6//OR52K2//OR52N2//OR52N4//OR52W1//OR56B1//OR56B4//OR5C1//OR5K2//OR9A4
Olfactory Signaling Pathway	1.08E-08	1.76E-06	43	OR10AD1//OR10G2//OR10H1//OR10H2//OR10H5//OR14I1//OR1G1//OR1J1//OR1J4//OR1K1//OR1L8//OR1N1//OR1Q1//OR2AE1//OR2AG2//OR2B11//OR2B2//OR2B6//OR2C1//OR2C3//OR2H2//OR2K2//OR2L13//OR2L2//OR2T33//OR2T8//OR2W3//OR3A1//OR3A2//OR51B4//OR51B5//OR51Q1//OR52B6//OR52K2//OR52N2//OR52N4//OR52W1//OR56B1//OR56B4//OR5C1//OR5K2//OR7D2//OR9A4
TASTE_RECEPTOR_ACTIVITY	1.41E-06	8.20E-05	7	TAS1R1//TAS1R2//TAS2R10//TAS2R13//TAS2R3//TAS2R4//TAS2R43
REACTOME_TRANSCRIPTION	2.60E-06	8.23E-05	24	H2AFB1//HIST1H2AD//HIST1H2BB//HIST1H2BF//HIST1H2BI//HIST1H2BL//HIST1H2BM//HIST1H2BO//HIST1H3A//HIST1H3B//HIST1H3C//HIST1H3F//HIST1H3I//HIST1H3J//HIST1H4A//HIST1H4B//HIST1H4C//HIST1H4D//HIST1H4F//HIST1H4I//HIST1H4K//HIST1H4L//HIST2H3C//HIST2H3D
Relaxin receptors	7.91E-06	0.00060333	5	INSL5//RLN3//RXFP1//RXFP2//RXFP4
Chromosome Maintenance	4.60E-05	0.0027681	15	FKBP6//H2AFB1//HIST1H2AD//HIST1H2AJ//HIST1H2BB//HIST1H2BL//HIST1H2BM//HIST1H2BO//HIST1H4A//HIST3H3//SMC1B//SYCP2//SYCP3//TERT//TEX12
SENSORY_PERCEPTION_OF_TASTE	5.85E-05	0.0020577	5	TAS1R1//TAS1R2//TAS2R3//TAS2R4//TAS2R43
REGULATION_OF_IMMUNE_RESPONSE	6.78E-05	0.0022662	8	CD79A//EREG//IFNK//IL29//IL4//KRT1//NCR1//TRAT1
REACTOME_RNA_POLYMERASE_I_CHAIN_ELONGATION	8.53E-05	0.0025026	8	HIST1H3A//HIST1H3B//HIST1H3C//HIST1H3F//HIST1H3I//HIST1H3J//HIST2H3C//HIST2H3D

Pancancer cluster 6

Geneset	Pvalue	FDR	NrGeneOverlap	GeneOverlap
Orexin and neuropeptides FF and QRFP bind to their respective receptor	4.19E-06	0.00020056	6	HCRT//HCRTR1//HCRTR2//NPFFR1//NPFFR2//QRFP
GAMETE_GENERATION	8.61E-06	0.00015299	23	BCL2L10//BMPR1B//CCNA1//DDX25//DNAH9//DNAJB13//FOXJ1//FSHR//HIST1H1A//HIST1H1T//LEFTY2//MORC1//NME5//PROK2//SERPINA5//SPAG11B//SPATA4//SYCP1//VCX//VCX3A//VCX3B//WFDC2//YBX2
SULFOTRANSFERASE_ACTIVITY	1.35E-05	0.00022751	10	CHST4//CHST5//CHST6//CHST8//CHST9//GAL3ST1//GAL3ST2//HS3ST5//HS3ST6//SULT1C2
TRANSFERASE_ACTIVITY__TRANSFERRING_SULFUR_CONTAINING_GROUPS	5.38E-05	0.00081247	10	CHST4//CHST5//CHST6//CHST8//CHST9//GAL3ST1//GAL3ST2//HS3ST5//HS3ST6//SULT1C2

Pancancer cluster 7

Geneset	Pvalue	FDR	NrGeneOverlap	GeneOverlap
MEMBRANE_FRACTION	2.05E-09	6.48E-08	52	A4GNT//ADAM18//ADCY8//APOB//CA4//CD70//CEACAM4//CYP2C9//CYP3A4//CYP3A5//CYP4A11//DCT//DSCAM//FMO3//FOLR1//GAL3ST1//GCNT3//GYPA//HSD17B3//HSD3B2//HTR4//MAL//MCF2//MLC1//NOS1//PDZD3//SIGLEC6//SLC12A1//SLC12A3//SLC13A2//SLC15A1//SLC17A1//SLC17A2//SLC17A3//SLC17A4//SLC18A3//SLC22A1//SLC22A13//SLC22A14//SLC22A2//SLC22A3//SLC22A7//SLC22A8//SLC23A1//SLC28A1//SLC2A2//SLC34A2//SLC3A1//SLC7A2//TM4SF4//TSPAN12//UGT2B7
SECONDARY_ACTIVE_TRANSMEMBRANE_TRANSPORTER_ACTIVITY	1.19E-07	2.87E-06	15	SLC10A2//SLC12A1//SLC12A3//SLC13A2//SLC15A1//SLC17A2//SLC17A3//SLC17A4//SLC22A11//SLC22A7//SLC4A4//SLC5A1//SLC5A2//SLC5A7//SLC9A3
SYMPORTER_ACTIVITY	1.98E-07	4.66E-06	12	SLC10A2//SLC12A1//SLC12A3//SLC13A2//SLC15A1//SLC17A2//SLC17A3//SLC17A4//SLC4A4//SLC5A1//SLC5A2//SLC5A7
KEGG_PORPHYRIN_AND_CHLOROPHYLL_METABOLISM	9.04E-07	1.05E-05	13	ALAS2//CP//UGT1A1//UGT1A10//UGT1A5//UGT1A6//UGT1A7//UGT1A8//UGT1A9//UGT2A3//UGT2B10//UGT2B11//UGT2B7

KEGG_PPAR_SIGNALING_PATHWAY	1.10E-06	1.18E-05	17	ACADL//ACSL6//ANGPTL4//APOC3//AQP7//CYP4A11//CYP4A22//CYP7A1//CYP8B1//FABP1//FABP2//FABP4//FABP6//FABP7//HMGCS2//PCK1//PLIN1
Organic cation/anion/zwitterion transport	5.18E-06	0.00020975	7	SLC22A1//SLC22A11//SLC22A12//SLC22A2//SLC22A3//SLC22A7//SLC22A8
BIOCARTA_INTRINSIC_PATHWAY	6.18E-06	0.0012989	9	COL4A3//F11//F2//FGA//FGB//FGG//KLKB1//KNG1//SERPINC1
REACTOME_COMMON_PATHWAY	9.79E-06	0.00018706	7	F2//FGA//FGB//FGG//PF4//PF4V1//SERPINC1
Common Pathway	9.79E-06	0.00038163	7	F2//FGA//FGB//FGG//PF4//PF4V1//SERPINC1
CARBOXYLIC_ACID_METABOLIC_PROCESS	1.31E-05	0.00020709	27	ACOT12//AGXT//AKR1C1//AKR1C2//AKR1D1//ALDH1L1//ASPA//BAAT//BBOX1//CYP4A11//CYP4F2//DCT//FTCD//GAD2//GLDC//GLYAT//HAO1//HAO2//HGD//HPD//NR1H4//PAH//PPARGC1A//SLC3A1//SLC7A2//SLC7A9//TYR
ORGANIC_ACID_METABOLIC_PROCESS	1.62E-05	0.00025336	27	ACOT12//AGXT//AKR1C1//AKR1C2//AKR1D1//ALDH1L1//ASPA//BAAT//BBOX1//CYP4A11//CYP4F2//DCT//FTCD//GAD2//GLDC//GLYAT//HAO1//HAO2//HGD//HPD//NR1H4//PAH//PPARGC1A//SLC3A1//SLC7A2//SLC7A9//TYR
BIOCARTA_AMI_PATHWAY	1.70E-05	0.0017872	8	AHSP//COL4A3//F2//FGA//FGB//FGG//PLG//SERPINC1
REACTOME_STEROID_HORMONES	2.59E-05	0.00040926	8	CUBN//CYP17A1//CYP21A2//CYP24A1//GC//HSD17B3//HSD3B2//LRP2
ANION_CATION_SYMPORTER_ACTIVITY	2.91E-05	0.00041196	7	SLC12A1//SLC12A3//SLC13A2//SLC17A2//SLC17A3//SLC17A4//SLC4A4
KEGG_RENIN_ANGIOTENSIN_SYSTEM	4.68E-05	0.00040635	7	ACE2//AGT//AGTR1//CMA1//CPA3//CTSG//REN
REACTOME_METABOLISM_OF_LIPIDS_AND_LIPOPROTEINS	5.25E-05	0.00073261	31	ABCG5//ACADL//AGT//AKR1C4//AKR1D1//ALB//AMN//ANGPTL4//APOB//APOC3//BAAT//CUBN//CYP17A1//CYP21A2//CYP4A11//CYP4A22//CYP7A1//CYP8B1//FABP4//FABP6//GPD1//HMGCS2//HSD17B3//HSD3B2//LIPC//MTTP//NPC1L1//PLIN1//SLC10A2//SLC2A2//UGT1A9
Metabolism of lipids and lipoproteins	5.50E-05	0.0018087	43	ABCG5//ACADL//ACSL6//AGT//AKR1C4//AKR1D1//ALB//AMN//ANGPTL4//ANKRD1//APOB//APOC3//BAAT//BCMO1//CUBN//CYP17A1//CYP21A2//CYP24A1//CYP4A11//CYP7A1//CYP8B1//ELOVL2//ENPP7//FABP1//FABP4//FABP6//GAL3ST1//GBA3//GC//GPD1//HMGCS2//HSD17B3//HSD3B2//LIPC//LRAT//LRP2//MTTP//NPC1L1//PLIN1//PPARGC1A//PRKAA2//SLC10A2//UGT1A9
BIOCARTA_ACE2_PATHWAY	7.76E-05	0.0054397	6	ACE2//AGT//AGTR1//CMA1//COL4A3//REN
PHOSPHATE_TRANSMEMBRANE_TRANSPORTER_ACTIVITY	7.76E-05	0.00092395	6	SLC17A1//SLC17A2//SLC17A3//SLC17A4//SLC34A1//SLC34A2
Glyoxylate metabolism	7.83E-05	0.0024252	4	AGXT//AGXT2//DAO//HAO1
Organic anion transport	7.83E-05	0.0023559	4	SLC22A11//SLC22A12//SLC22A7//SLC22A8
MONOCARBOXYLIC_ACID_METABOLIC_PROCESS	8.32E-05	0.00097113	16	ACOT12//AGXT//AKR1C1//AKR1C2//AKR1D1//ALDH1L1//BAAT//BBOX1//CYP4A11//CYP4F2//FTCD//GLYAT//HAO1//HAO2//NR1H4//PPARGC1A
REACTOME_PLATELET_DEGRANULATION	9.62E-05	0.001294	16	ACTN2//ALB//EGF//FGA//FGB//FGG//FIGF//GP5//GP9//HRG//KNG1//PF4//PLG//PPBP//SCG3//TF

Pancancer cluster 8

Geneset	Pvalue	FDR	NrGeneOverlap	GeneOverlap
KEGG_JAK_STAT_SIGNALING_PATHWAY	2.41E-09	1.19E-07	32	CBLC//CNTFR//CRLF2//CSF2//CSF3//EPO//IFNA1//IFNA13//IFNE//IFNG//IFNK//IL11//IL12B//IL13//IL13RA2//IL19//IL20//IL20RA//IL20RB//IL21//IL22RA1//IL22RA2//IL23R//IL24//IL26//IL28A//IL28B//IL29//IL6//LEP//TPO//TSLP
STRUCTURAL_CONSTITUENT_OF_MUSCLE	2.49E-09	5.18E-08	14	ACTC1//ACTN2//ACTN3//KRT19//MYBPC1//MYBPC2//MYBPC3//MYBPH//MYH6//MYH7//MYL2//MYL3//MYLPF//MYOT
RHYTHMIC_PROCESS	7.46E-08	1.26E-06	12	BMPR1B//EREG//FOXL2//MTNR1A//OPN4//SPRR2A//SPRR2B//SPRR2C//SPRR2D//SPRR2E//SPRR2F//SPRR2G
CONTRACTILE_FIBER	1.23E-07	2.01E-06	11	ABRA//ACTA1//DES//KRT19//MYBPC1//MYL3//MYLPF//MYOZ2//TNNC1//TNNI3//TNNT2
CONTRACTILE_FIBER_PART	5.34E-07	8.17E-06	10	ABRA//ACTA1//DES//KRT19//MYL3//MYLPF//MYOZ2//TNNC1//TNNI3//TNNT2
MYOFIBRIL	8.21E-07	1.21E-05	9	ABRA//ACTA1//DES//KRT19//MYBPC1//MYOZ2//TNNC1//TNNI3//TNNT2
REGULATION_OF_MUSCLE_CONTRACTION	8.21E-07	1.19E-05	9	KCNB2//MYBPC3//MYBPH//MYL2//NMU//PROK2//TNNC1//TNNC2//TNNT1
il23pathway	1.66E-06	0.00034762	12	ALOX12B//CXCL1//CXCL9//IFNG//IL12B//IL17A//IL17F//IL19//IL23R//IL24//IL6//NOS2

CALCIUM_ION_BINDING	3.83E-06	5.25E-05	20	ANXA10//CALB1//CALML3//COMP//DMP1//DNASE1L3//DUOX2//FLG//GPR98//HPCAL4//ITIH1//MYL2//PRSS3//S100A12//S100A7//S100A8//S100A9//S100P//TGM3//THBS4
HEART_DEVELOPMENT	8.64E-06	0.00011698	11	BMP2//CASQ2//FGF12//GATA4//HAND2//IRX4//MYBPC3//MYH6//MYH7//SHOX2//TBX1
SARCOMERE	9.25E-06	0.00012378	7	ABRA//ACTA1//DES//MYOZ2//TNNC1//TNNI3//TNNT2
REGULATION_OF_RESPONSE_TO_STIMULUS	1.40E-05	0.0001848	14	AHSG//CD79A//CHRNA7//EREG//IFNK//IL12B//IL20//IL29//IL8//KLK8//KRT1//LEP//NCR1//SPINK5
CYTOSKELETON	1.61E-05	0.00021003	44	ABRA//ACTA1//ACTC1//ACTL7B//ACTN2//ACTN3//BFSP2//CCNA1//CSTA//CTAG2//DNAI2//DSP//EPB41L4B//HAP1//IVL//KRT1//KRT15//KRT17//KRT19//KRT2//KRT3//KRT5//KRT6A//LOR//MYH6//MYH7//MYL2//MYL3//MYLPF//MYO18B//MYO3A//MYOT//NEFL//NEFM//PKP1//SCEL//SPRR1A//SPRR1B//SPRR3//TGM1//TNNC1//TNNI3//TNNT2//USH1G
KEGG_ECM_RECEPTOR_INTERACTION	1.70E-05	0.00016756	17	COL11A1//COL1A1//COL2A1//COL4A6//COL6A6//COMP//IBSP//ITGB6//LAMA1//LAMA3//LAMB3//LAMB4//LAMC2//RELN//SV2C//THBS4//TNN
DEVELOPMENTAL_GROWTH	2.22E-05	0.00028143	6	BMPR1B//DMBX1//EREG//KLK8//RTN4RL1//SPINK5
METALLOPEPTIDASE_ACTIVITY	2.59E-05	0.00030755	12	CPA2//CPA3//ECEL1//FAP//MEP1A//MMP11//MMP12//MMP13//MMP16//MMP3//MMP8//PAPPA2
Presynaptic nicotinic acetylcholine receptors	4.21E-05	0.0017042	6	CHRNA1//CHRNA2//CHRNA6//CHRNB4//CHRND//CHRNG
Binding of RNA by Insulin-like Growth Factor-2 mRNA Binding Proteins (	4.80E-05	0.0018079	5	H19//IGF2//IGF2BP1//IGF2BP2//IGF2BP3
INNATE_IMMUNE_RESPONSE	5.24E-05	0.00059786	8	DEFB1//EREG//IFNK//IL12B//KRT1//NCR1//PYDC1//SFTPD
CATION_BINDING	5.31E-05	0.00060016	29	ANXA10//CA12//CALB1//CALML3//CBLC//COMP//DMP1//DNASE1L3//DUOX2//FLG//GDA//GPR98//HPCAL4//ITIH1//MMP1//MMP10//MMP12//MMP13//MMP16//MMP8//MYL2//PRSS3//S100A12//S100A7//S100A8//S100A9//S100P//TGM3//THBS4
MUSCLE_DEVELOPMENT	5.80E-05	0.00064882	17	ACTA1//CHRNA1//CSRP3//FOXL2//GYLTL1B//KRT19//MUSK//MYBPC3//MYF6//MYH6//MYH7//MYOD1//MYOZ1//SGCA//SGCG//SIX1//TNNI3
TRANSCRIPTION_FACTOR_ACTIVITY	6.16E-05	0.00068291	41	ALX1//BNC1//DLX2//DLX3//DLX6//DMBX1//DMRT1//DMRT2//EHF//FOXA1//FOXD1//FOXD3//FOXE1//FOXE3//FOXL1//FOXL2//GATA1//GBX2//HAND2//HOXC5//LMO1//LMX1B//MAFA//MYF6//MYOD1//MYOG//NKX2-5//PAX3//PAX7//PITX2//POU3F1//POU3F2//POU4F3//POU6F2//RORB//SHOX//TBX1//TBX4//TFAP2C//TRIM29//ZIC1

Pancancer cluster 9

**Geneset**	**Pvalue**	**FDR**	**NrGeneOverlap**	**GeneOverlap**
FGFR2 ligand binding and activation	1.02E-05	0.0006119	8	FGF17//FGF18//FGF20//FGF22//FGF23//FGF3//FGF8//FGF9
FGFR4 ligand binding and activation	1.22E-05	0.00065929	7	FGF17//FGF18//FGF19//FGF20//FGF23//FGF8//FGF9
REACTOME_PHOSPHOLIPASE_CMEDIATED_CASCADE	1.76E-05	0.00036998	9	FGF17//FGF18//FGF19//FGF20//FGF22//FGF23//FGF3//FGF8//FGF9
FGFR ligand binding and activation	1.76E-05	0.00090615	9	FGF17//FGF18//FGF19//FGF20//FGF22//FGF23//FGF3//FGF8//FGF9
Steroid hormones	2.20E-05	0.0010837	10	CGA//CUBN//CYP11A1//CYP17A1//CYP21A2//CYP24A1//HSD17B3//LHB//SRD5A2//STARD6
PROTEASE_INHIBITOR_ACTIVITY	2.24E-05	0.00039332	12	AMBP//CST1//CST2//CST6//LPA//PI3//SERPINA4//SERPINA5//SERPINA6//SERPINB3//SERPINI2//SPINK5
FGFR1 ligand binding and activation	2.28E-05	0.0010297	7	FGF17//FGF20//FGF22//FGF23//FGF3//FGF8//FGF9
FGFR3b ligand binding and activation	3.72E-05	0.0016085	5	FGF17//FGF18//FGF20//FGF8//FGF9
REGULATION_OF_CELL_DIFFERENTIATION	6.39E-05	0.00097145	14	BMPR1B//CALCA//CARTPT//CNTN4//GPR98//IL20//IL4//INHA//NANOG//PF4//SHH//SPINK5//TWIST2//VWC2
REACTOME_SHCMEDIATED_CASCADE	6.55E-05	0.0012399	8	FGF17//FGF18//FGF20//FGF22//FGF23//FGF3//FGF8//FGF9
REACTOME_FGFR_LIGAND_BINDING_AND_ACTIVATION	7.73E-05	0.0013941	9	FGF17//FGF18//FGF19//FGF20//FGF22//FGF23//FGF3//FGF8//FGF9
REACTOME_FRS2MEDIATED_CASCADE	7.73E-05	0.0013307	9	FGF17//FGF18//FGF19//FGF20//FGF22//FGF23//FGF3//FGF8//FGF9

FGFR3 ligand binding and activation	9.14E-05	0.0031911	6	FGF17//FGF18//FGF20//FGF23//FGF8//FGF9
FGFR3c ligand binding and activation	9.14E-05	0.0030914	6	FGF17//FGF18//FGF20//FGF23//FGF8//FGF9
ENZYME_INHIBITOR_ACTIVITY	9.72E-05	0.0014446	21	AMBP//APOA2//CABP1//CST1//CST2//CST6//LPA//PI3//PPP1R1A//PPP1R1B//PTN//SAG//SERPINA4//SERPINA5//SERPINA6//SERPINB3//SERPINI2//SFN//SNCB//SPINK5//TESC

Pancancer cluster 10

Geneset	Pvalue	FDR	NrGeneOverlap	GeneOverlap
SODIUM_ION_TRANSPORT	4.22E-06	8.33E-05	8	ATP1A4//SCN10A//SCN2A//SCN2B//SCN4A//SCN7A//SCNN1A//SCNN1G
HORMONE_METABOLIC_PROCESS	1.82E-05	0.00031927	9	AKR1D1//CHST8//CHST9//CYP11A1//DHRS2//DIO1//DIO2//UGT2B11//UGT2B4
DETECTION_OF_ABIOTIC_STIMULUS	2.22E-05	0.00038461	7	ABCA4//GRM6//NPFFR2//OPN4//RP1//SAG//TAC1
G_PROTEIN_SIGNALING__ADENYLATE_CYCLASE_INHIBITING_PATHWAY	6.22E-05	0.00099318	5	DRD2//GRM4//MCHR1//NPY1R//NPY2R
REGULATION_OF_NEUROTRANSMITTER_LEVELS	9.08E-05	0.0013482	7	CARTPT//GRM4//RIMS1//SLC5A7//SLC6A4//SYN3//SYT1
